# Supplementary material for: PARP1 condensates differentially partition DNA repair proteins and enhance DNA ligation
Source: EMBO Rep. 2024 Nov 4;25(12):5635–66. doi: 10.1038/s44319-024-00285-5 (PMC11624282; doi:10.1038/s44319-024-00285-5)
Supplement: Supplementary file 1 — Appendix [file 44319_2024_285_MOESM1_ESM.pdf]

PARP1 condensates differentially partition DNA repair proteins and enhance DNA ligation

Christopher Chin Sang[a,#], Gaelen Moore[a,#], Maria Tereshchenko[a,^], Hongshan Zhang[c,d,^], Michael L. Nosella[a,b,^], Morgan Dasovich[f], T. Reid Alderson[e], Anthony K. L. Leung[f,g], Ilya J. Finkelstein[c,d], Julie D. Forman-Kay[a,b], Hyun O. Lee[a, *]

Table of Contents

Appendix Table S1 Page 2

Appendix Table S2 Page 3

Appendix Table S1. Phase separation predictions for proteins investigated in this study using PScore, CatGRANULE, and PLAAC prediction algorithms. PScore (Vernon *et al*, 2018) uses pi-pi interactions, PLAAC (Lancaster *et al*, 2014) uses prion-like domain features, and catGRANULE (Bolognesi *et al*, 2016) uses similarities to sequence features found in RNA granule components. Values reflect percentiles of scores within the human proteome, with 100 percent indicating the highest likelihood for phase separation (Vernon & Forman-Kay, 2019).

**Name**	**UniProt ID**	**PScore**	**CatGRANULE**	**PLAAC**
**PARP1**	P09874_HUMAN	62.36	96.31	54.59
**XRCC1**	P18887_HUMAN	65.47	86.39	73.13
**LigIII**	P49916_HUMAN	66.35	86.10	58.98
**Polβ**	P06746_HUMAN	18.36	82.30	9.743

Appendix Table S2. Sequences of oligonucleotides used in this study.

Nicked dumbbell DNA	5’-P-GCTGGCTTCGTAAGAAGCCAGCTCGCGGTCAGCTTGCTGACCGCG-3’	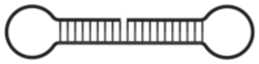
Cy5-triplex	5’-GCTTAGCTTGGAATCGTATCATGTACACTCGTGTGCCGTGTAGACCGTGCC-3’<br>5’-P-TGTACATGATACGATTCCAAGCTAAGC-3’<br>5’-Cy5-GGCACGGTCTACACGGCACACGAG-3’	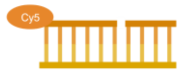
20 nt DNA	5’-ATCTTGCTGATGGTCTAGGC-3’	
40 nt DNA	5’-ACCGCCAAGTCGGTTTCGACTCATACCGCAGCCCTCTATA-3’	
60 nt DNA	5’-GATGGTGGGGTCCGTTGATGTCAAGCATAGTCGAAAAAGGGTGCGGGACGACAACCCATG-3’	
20 nt DNA RC	5’-GCCTAGACCATCAGCAAGAT-3’	
40 nt DNA RC	5’-TATAGAGGGCTGCGGTATGAGTCGAAACCGACTTGGCGGT-3’	
60 nt DNA RC	5’-CATGGGTTGTCGTCCCGCACCCTTTTTCGACTATGCTTGACATCAACGGACCCCACCATC-3’	
Lab07	5’-P-AGGTCGCCGCCC-BioTEG-3’	
Lab06	5’-Phos/GGGCGGCGACCT-BioTEG-3’	
Oligo1	5’-ACGAAGTCTTATGGCAAAACCGATGGACTATGTTTCGGGTAGCACCAGAAGTCTATAACA-3’	
Oligo2	5’-TGTTATAGACTTCTGGTGCTACCCGAAACATAGTCCATCGGTTTTGCCATAAGACTTCGT-ATTO647N-3’	
