# Supplementary material for: PARP1 condensates differentially partition DNA repair proteins and enhance DNA ligation
Source: EMBO Rep. 2024 Nov 4;25(12):5635–66. doi: 10.1038/s44319-024-00285-5 (PMC11624282; doi:10.1038/s44319-024-00285-5)
Supplement: Supplementary file 3 — Movie EV1 [file 44319_2024_285_MOESM3_ESM.zip › MovieEV1/Movie EV1_legend.docx]

Movie EV1: A representative movie showing the end-to-end bridging of two DNA molecules following the injection of 100 μL of 10 nM PARP1. The bridging event can be reversed by continuously injecting 500 μM NAD+ for 400 s. Related to Fig. 4G.
