# Supplementary material for: PARP1 condensates differentially partition DNA repair proteins and enhance DNA ligation
Source: EMBO Rep. 2024 Nov 4;25(12):5635–66. doi: 10.1038/s44319-024-00285-5 (PMC11624282; doi:10.1038/s44319-024-00285-5)
Supplement: Supplementary file 4 — Movie EV2 [file 44319_2024_285_MOESM4_ESM.zip › MovieEV2/MovieEV2_legend.docx]

Movie EV2: A representative movie showing the end-to-end bridging of two DNA molecules following the injection of 100 μL of 10 nM PARP1-E988Q. This bridge is not reversed by continuously injecting 500 μM NAD+ for 825 s. Related to Fig. EV 4G.
