# Supplementary figures and images for: PARP1 condensates differentially partition DNA repair proteins and enhance DNA ligation
Source: EMBO Rep. 2024 Nov 4;25(12):5635–66. doi: 10.1038/s44319-024-00285-5 (PMC11624282; doi:10.1038/s44319-024-00285-5)

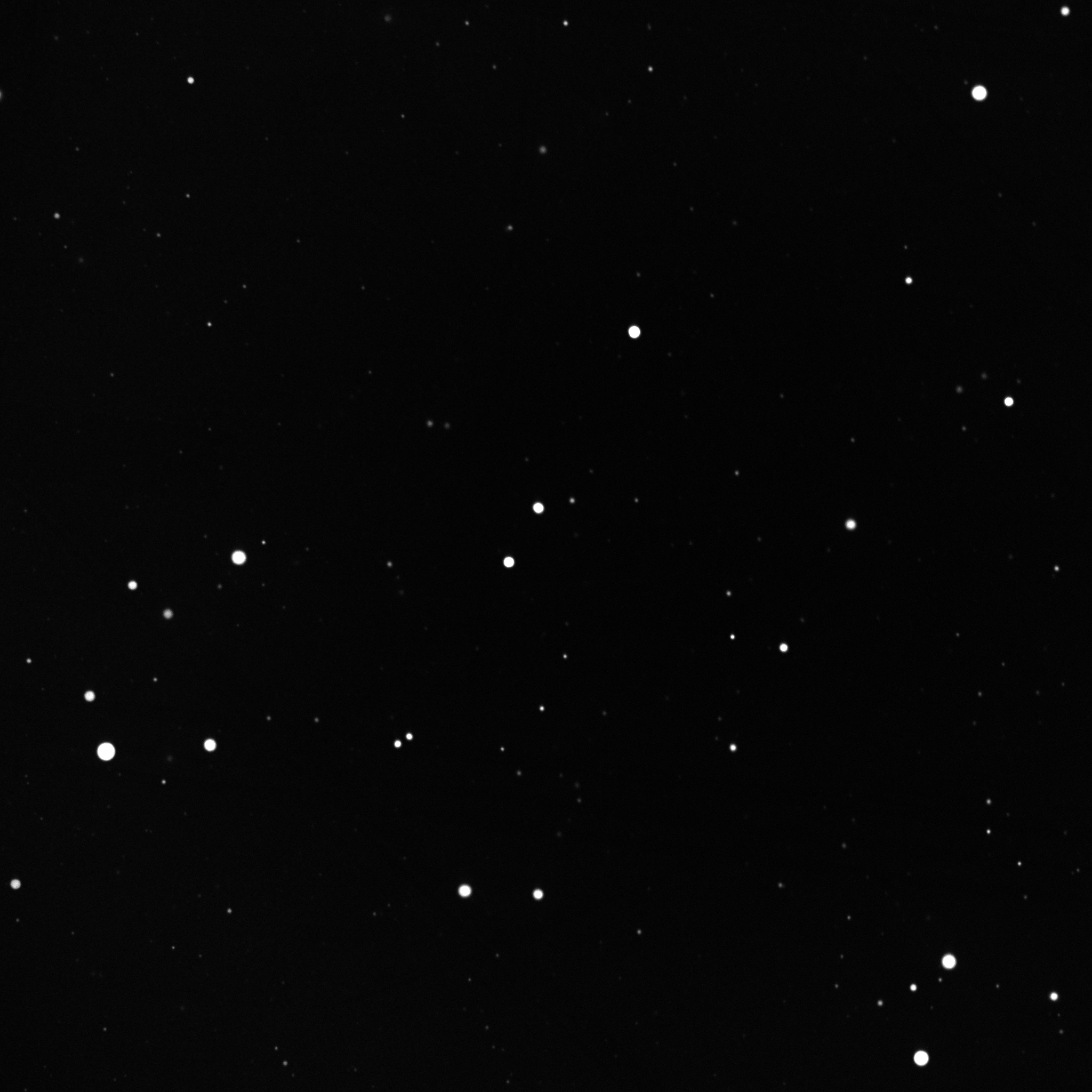

Supplement: Supplementary file 6 — Source data Fig. 1 [file 44319_2024_285_MOESM6_ESM.zip › Fig1/1C/0.5uM mCh-PARP1 2uM Triplex DNA.tif]

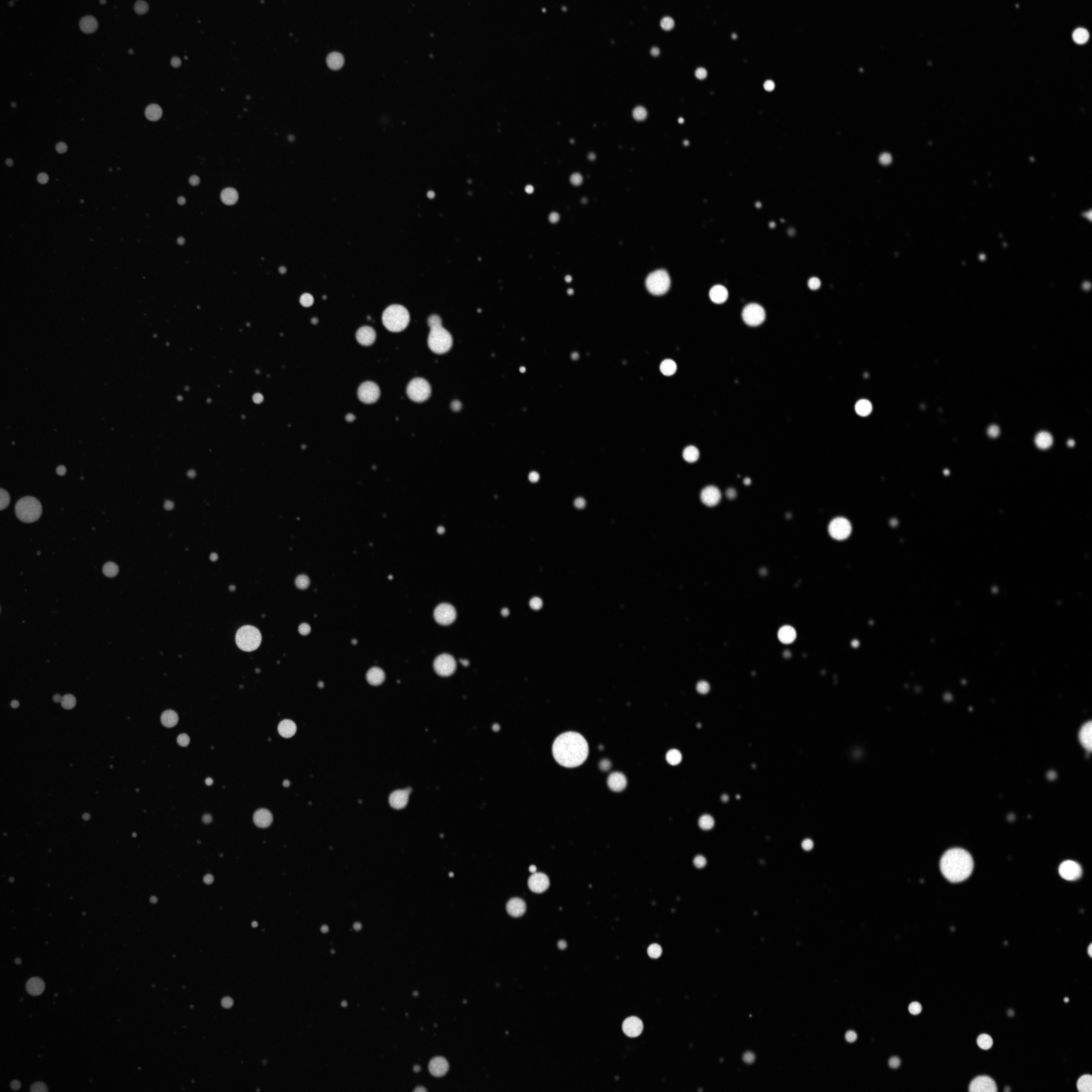

Supplement: Supplementary file 6 — Source data Fig. 1 [file 44319_2024_285_MOESM6_ESM.zip › Fig1/1C/1uM mCh-PARP1 2uM Triplex DNA.tif]

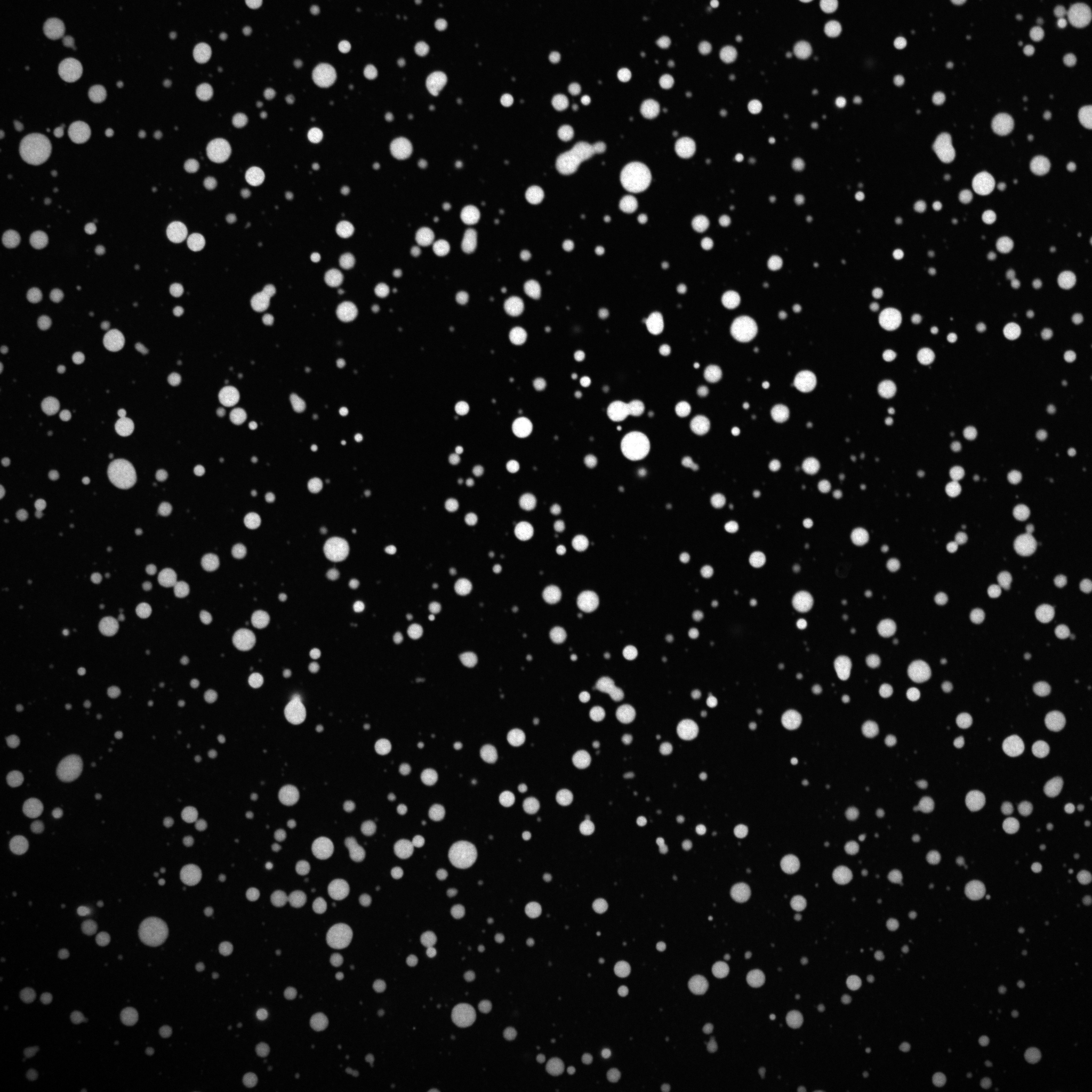

Supplement: Supplementary file 6 — Source data Fig. 1 [file 44319_2024_285_MOESM6_ESM.zip › Fig1/1C/2uM mCh-PARP1 2uM Triplex DNA.tif]

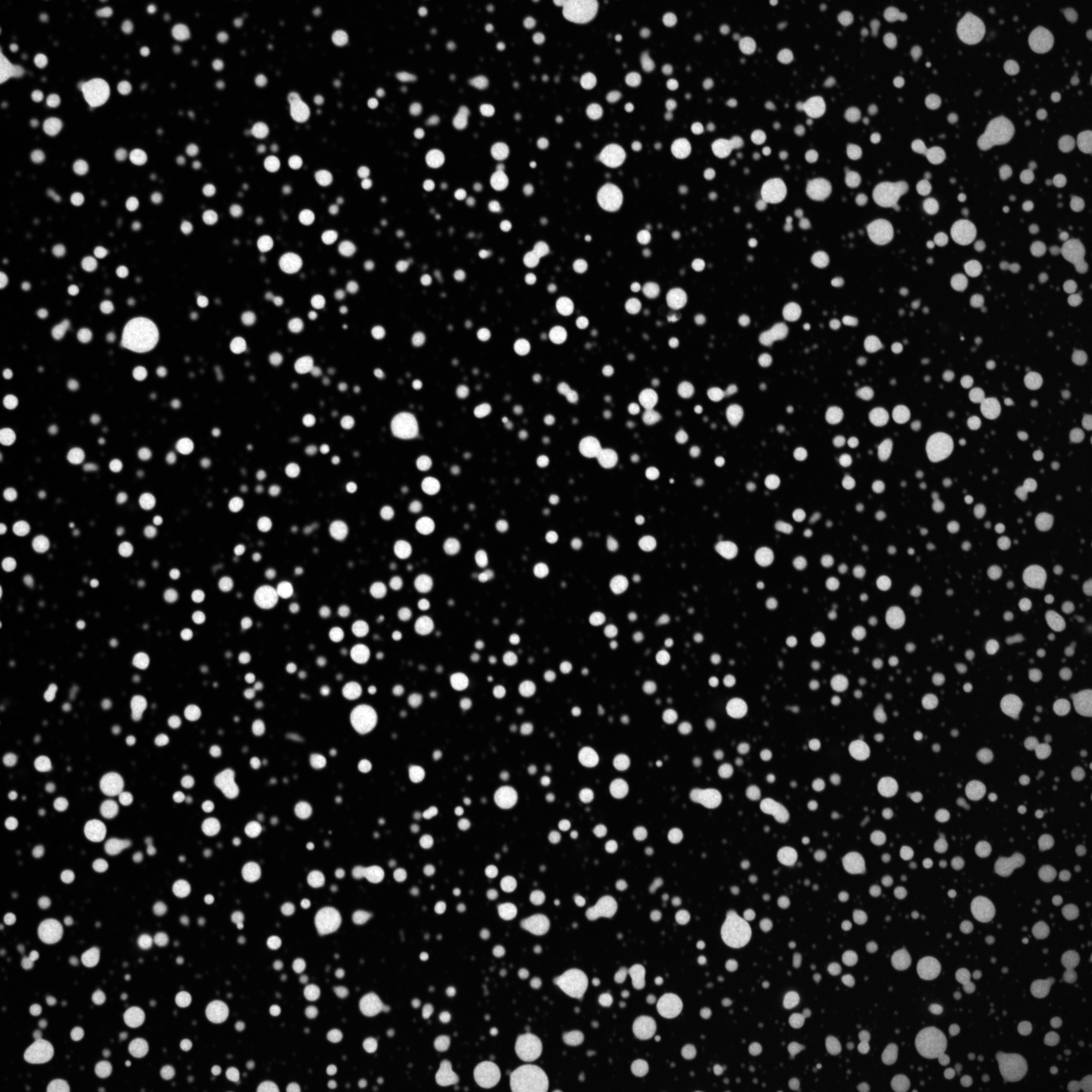

Supplement: Supplementary file 6 — Source data Fig. 1 [file 44319_2024_285_MOESM6_ESM.zip › Fig1/1C/4uM mCh-PARP1 2uM Triplex DNA.tif]

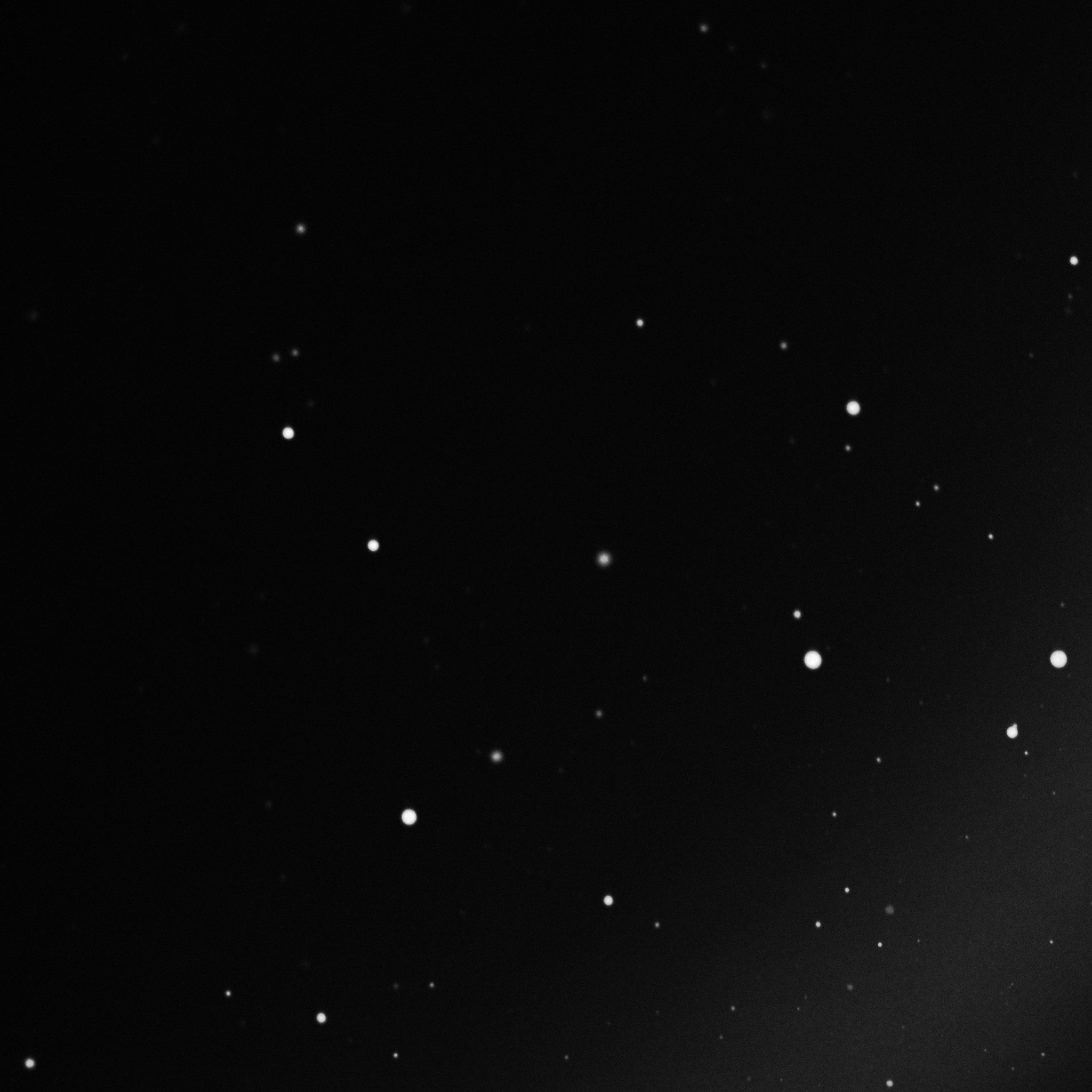

Supplement: Supplementary file 6 — Source data Fig. 1 [file 44319_2024_285_MOESM6_ESM.zip › Fig1/1C/8uM mCh-PARP1 2uM Triplex DNA.tif]

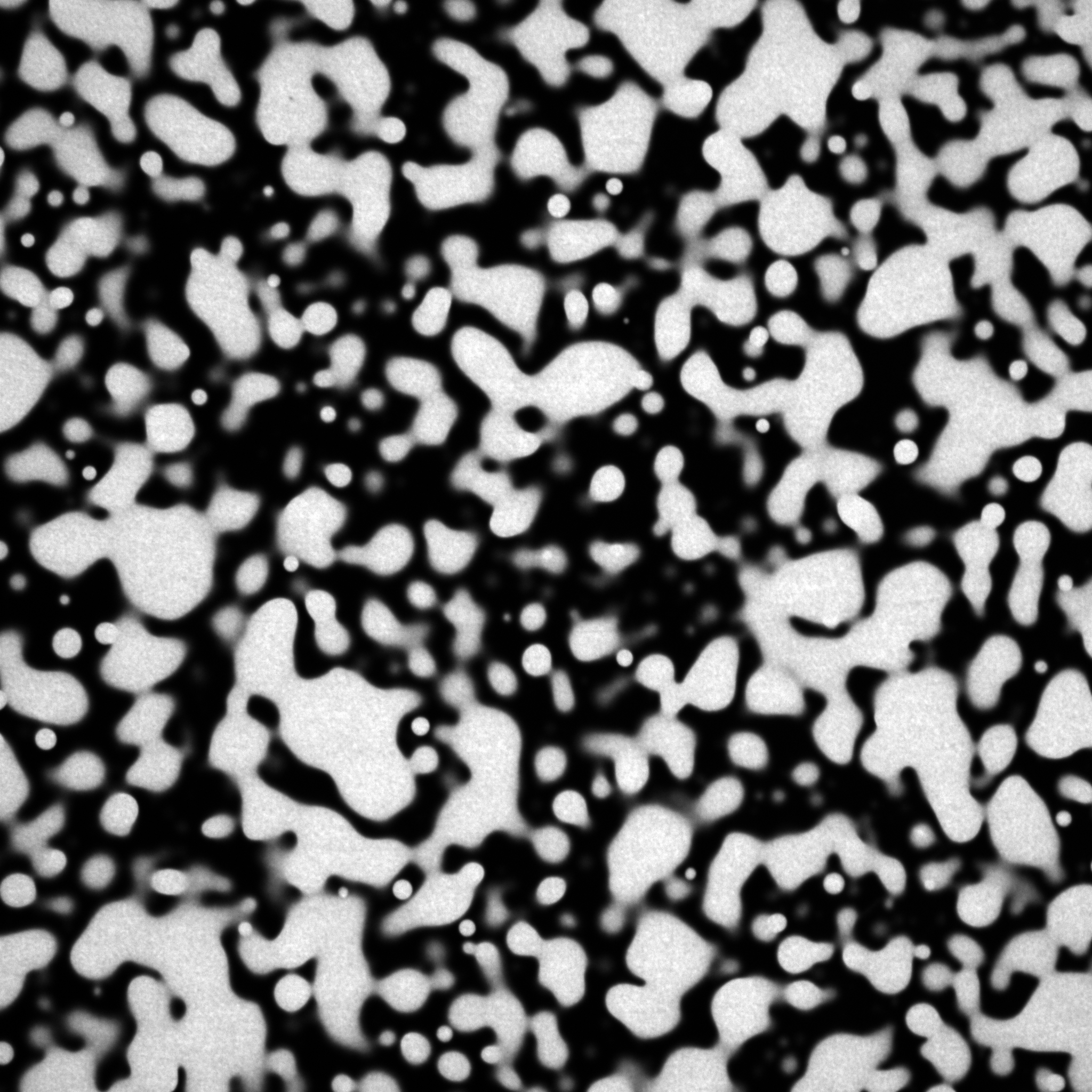

Supplement: Supplementary file 6 — Source data Fig. 1 [file 44319_2024_285_MOESM6_ESM.zip › Fig1/1E/4uM mCh-PARP1 16uM Triplex DNA.tif]

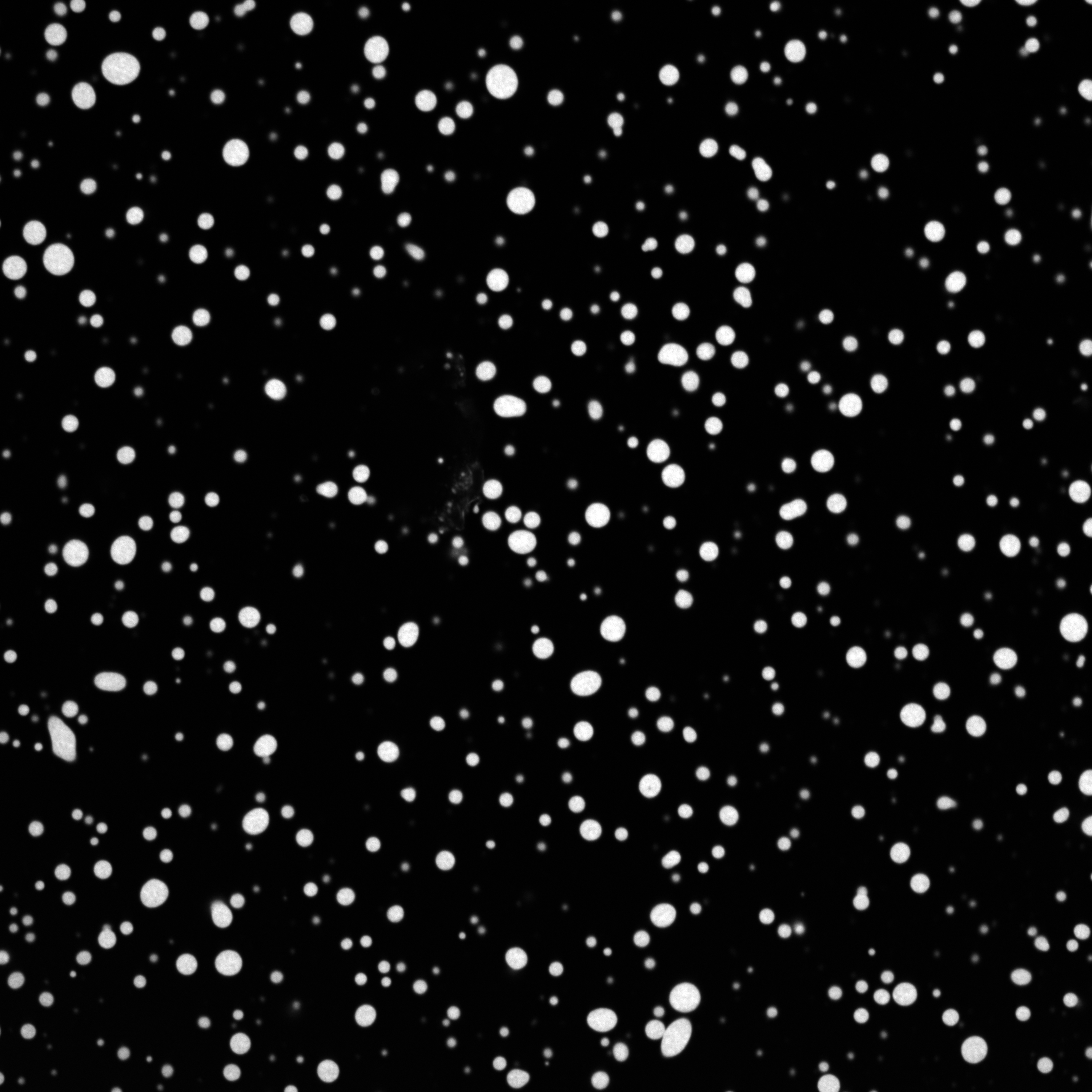

Supplement: Supplementary file 6 — Source data Fig. 1 [file 44319_2024_285_MOESM6_ESM.zip › Fig1/1E/4uM mCh-PARP1 1uM Triplex DNA.tif]

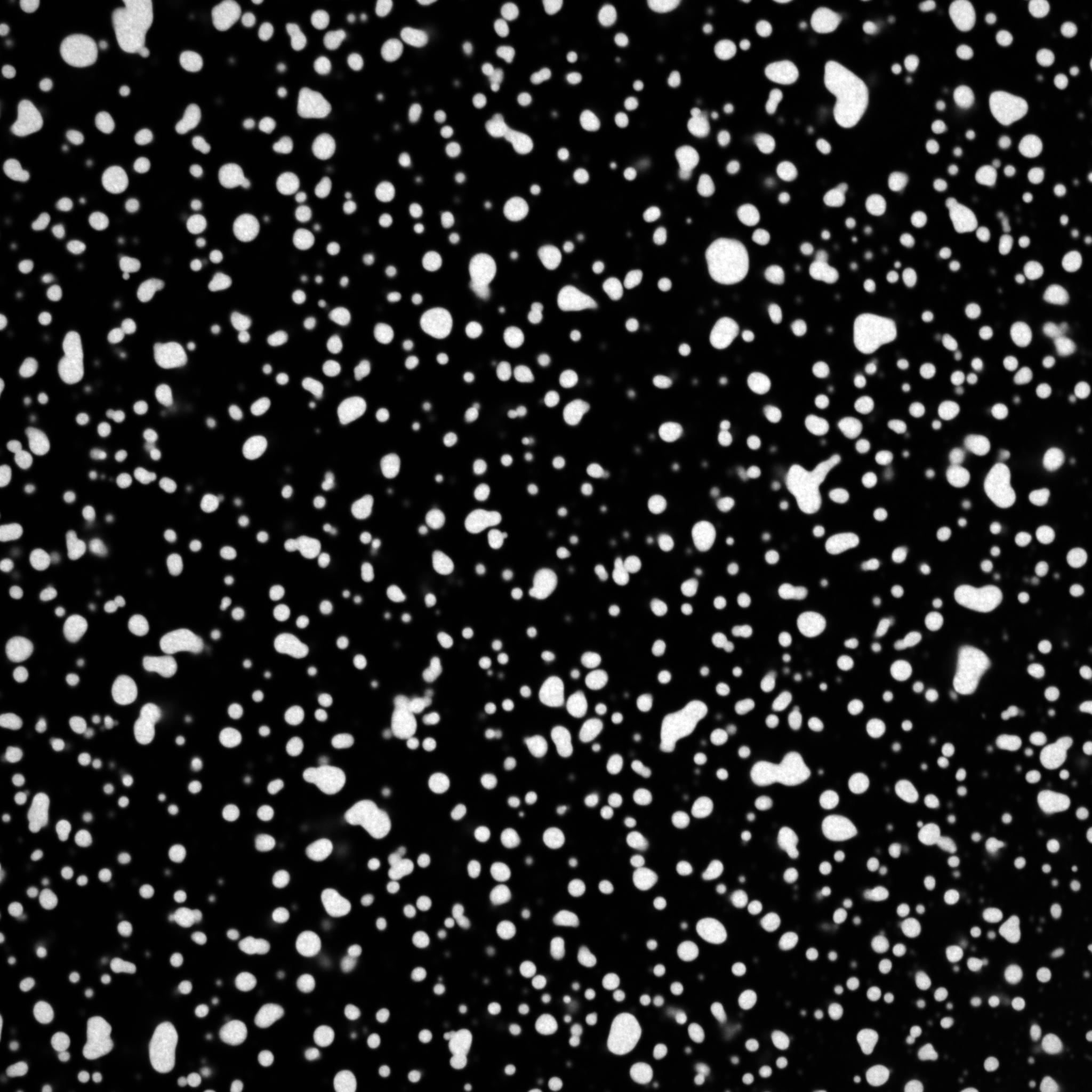

Supplement: Supplementary file 6 — Source data Fig. 1 [file 44319_2024_285_MOESM6_ESM.zip › Fig1/1E/4uM mCh-PARP1 2uM Triplex DNA.tif]

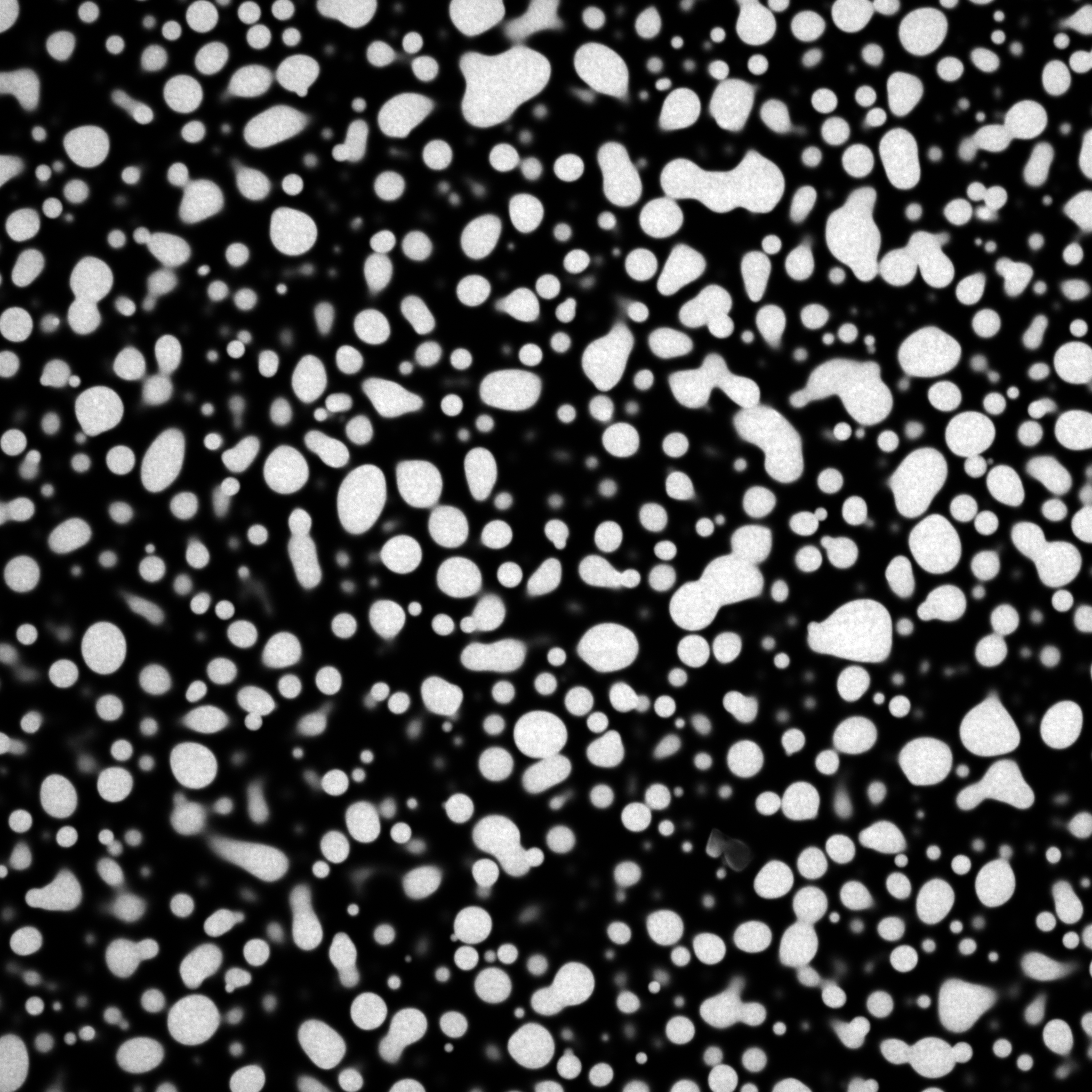

Supplement: Supplementary file 6 — Source data Fig. 1 [file 44319_2024_285_MOESM6_ESM.zip › Fig1/1E/4uM mCh-PARP1 4uM Triplex DNA.tif]

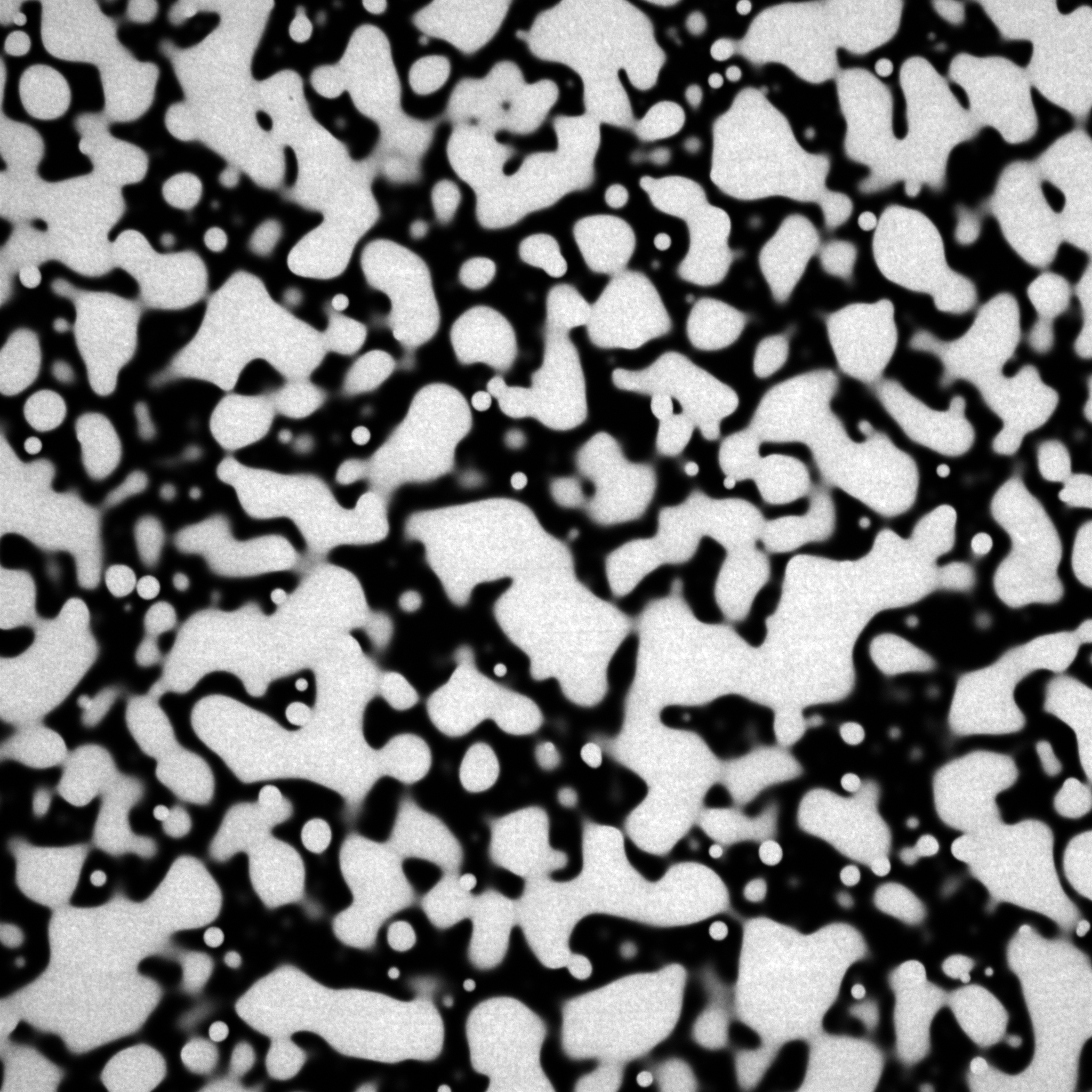

Supplement: Supplementary file 6 — Source data Fig. 1 [file 44319_2024_285_MOESM6_ESM.zip › Fig1/1E/4uM mCh-PARP1 8uM Triplex DNA.tif]

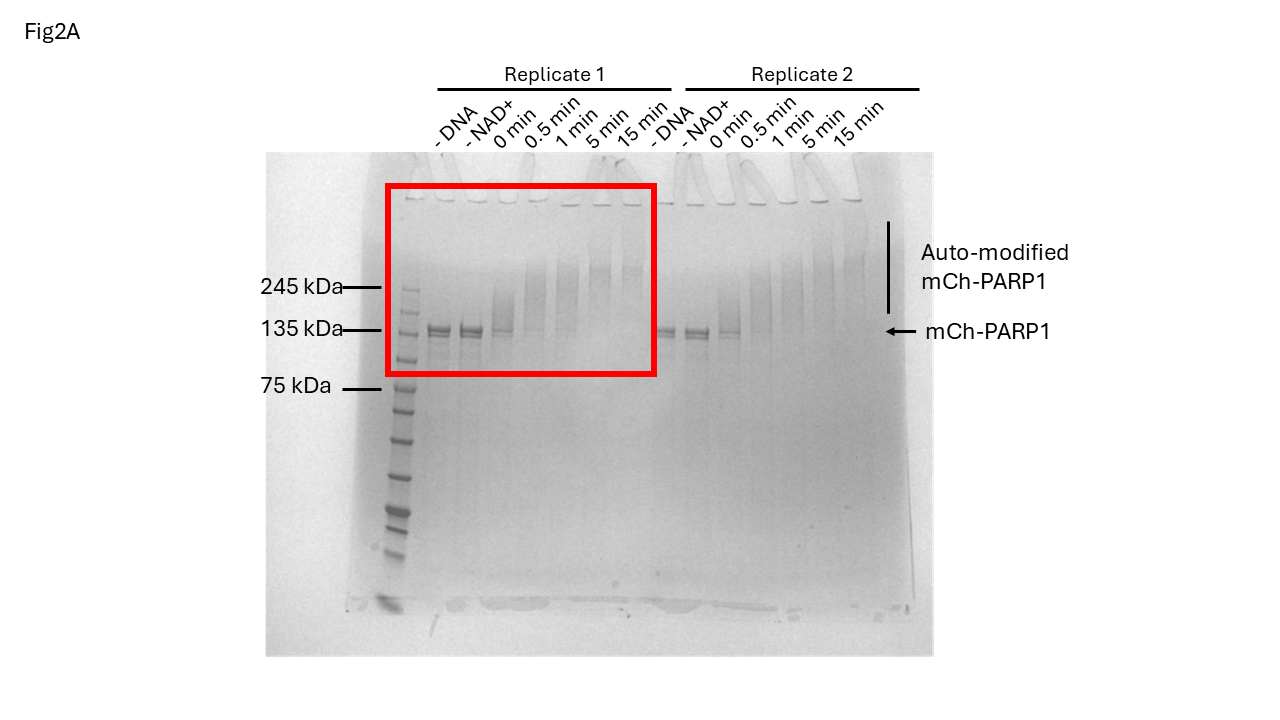

Supplement: Supplementary file 7 — Source data Fig. 2 [file 44319_2024_285_MOESM7_ESM.zip › Fig2/Fig2A/PARylation_Gel.tif]

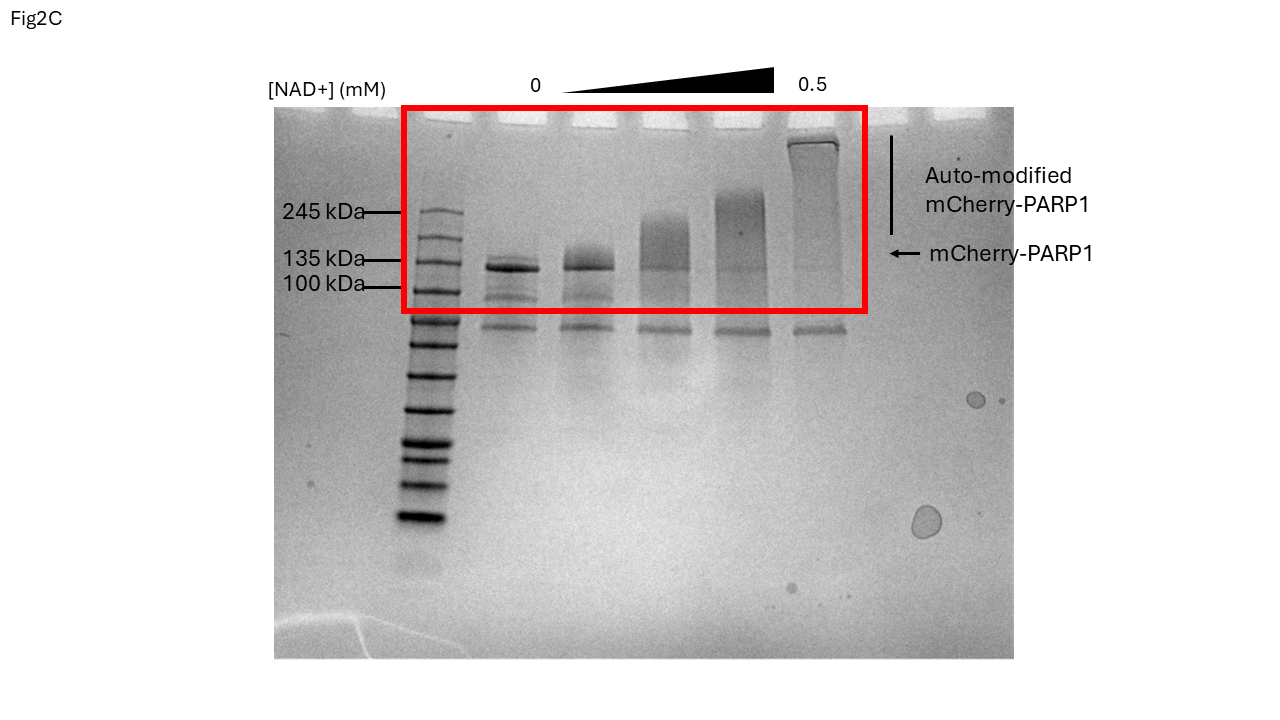

Supplement: Supplementary file 7 — Source data Fig. 2 [file 44319_2024_285_MOESM7_ESM.zip › Fig2/Fig2C/NAD_Titration_Gel.tif]

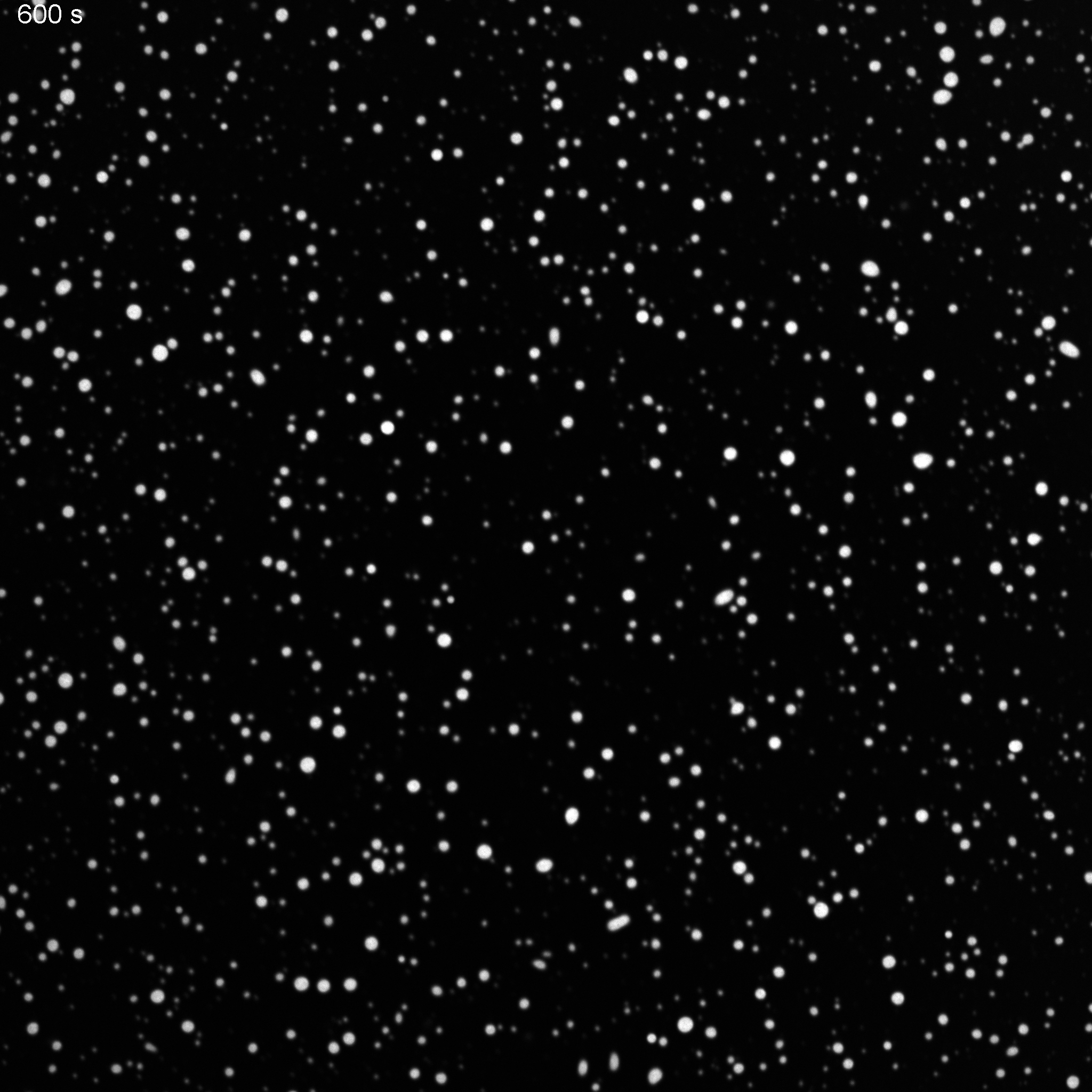

Supplement: Supplementary file 7 — Source data Fig. 2 [file 44319_2024_285_MOESM7_ESM.zip › Fig2/Fig2E/Buffer +600s.tif]

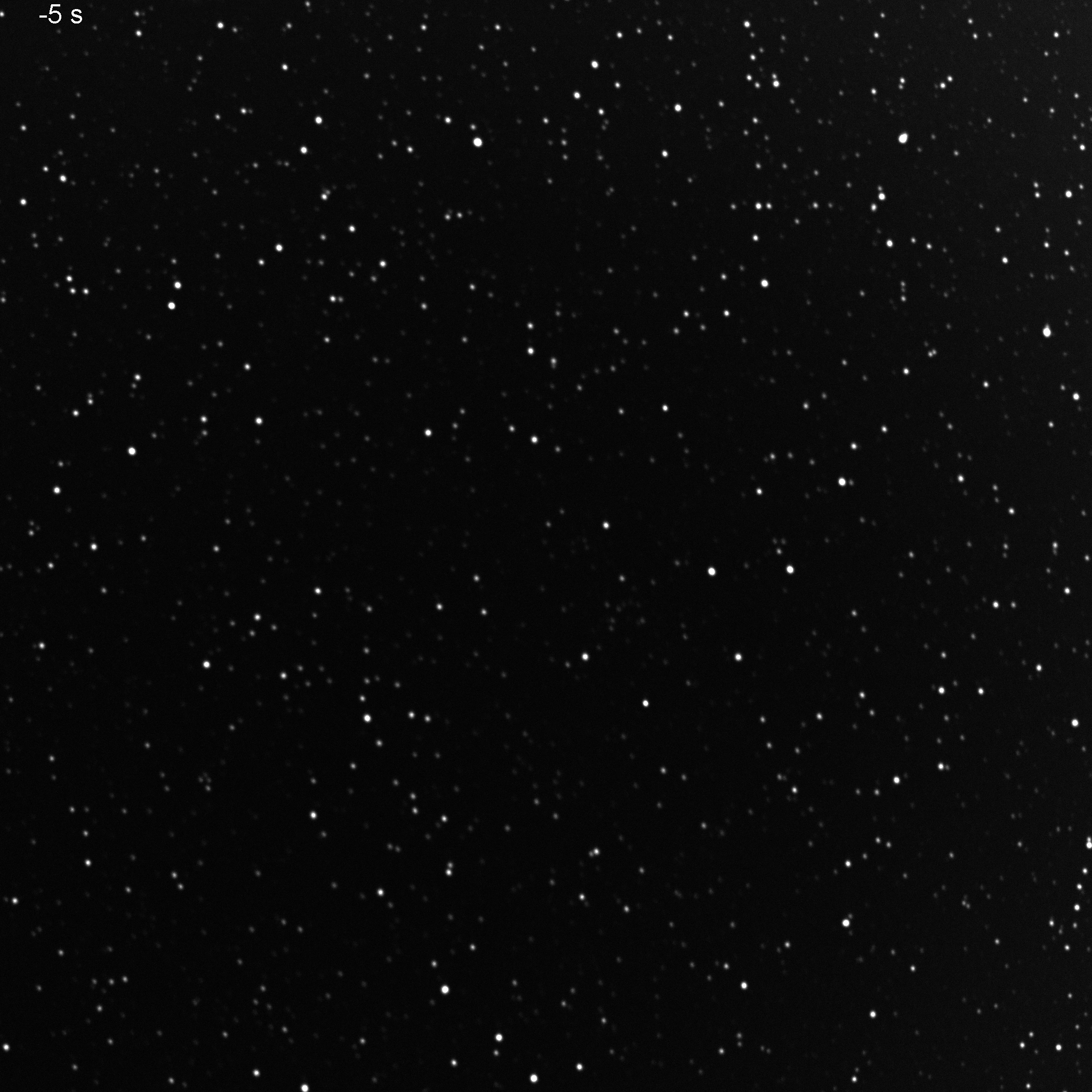

Supplement: Supplementary file 7 — Source data Fig. 2 [file 44319_2024_285_MOESM7_ESM.zip › Fig2/Fig2E/Buffer -5s.tif]

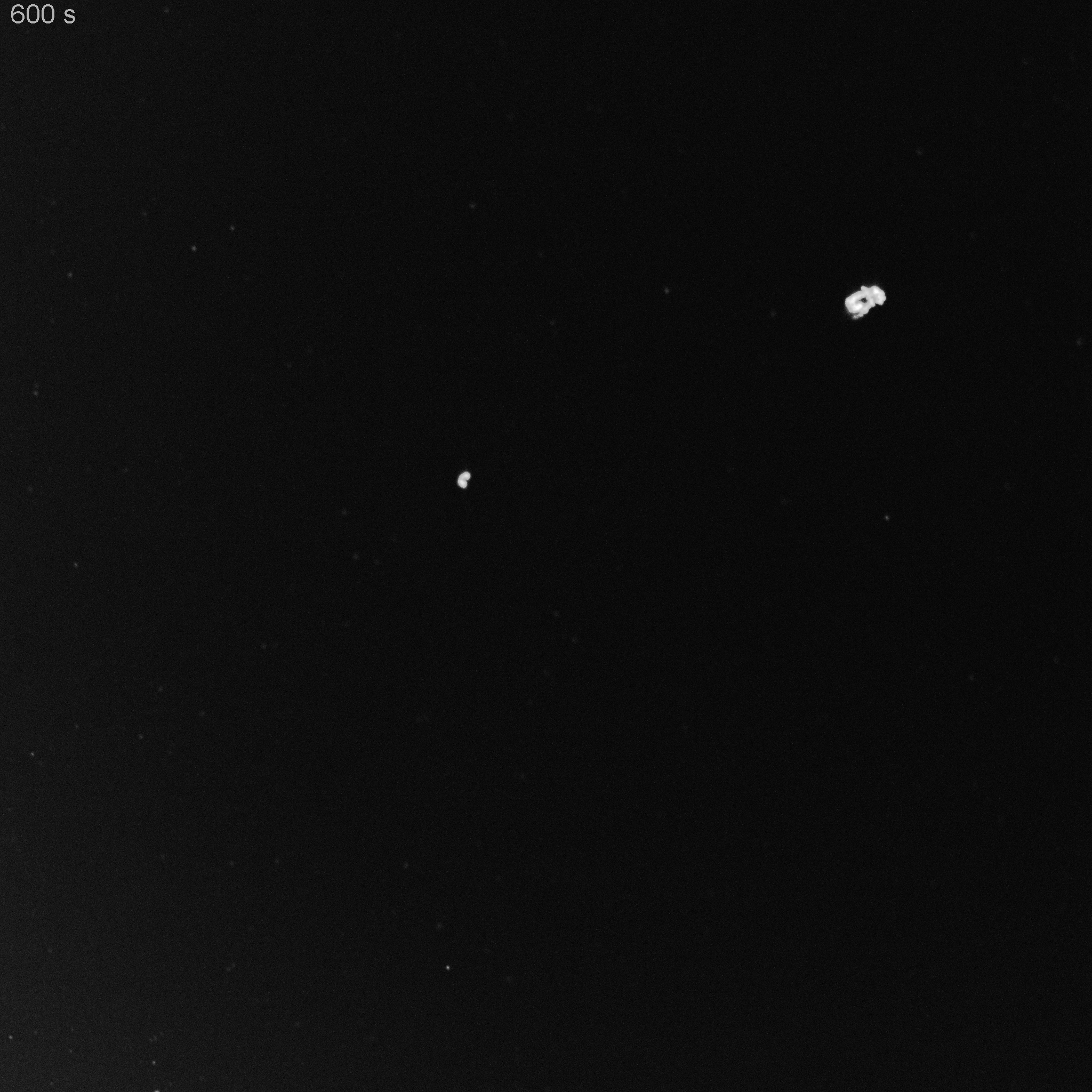

Supplement: Supplementary file 7 — Source data Fig. 2 [file 44319_2024_285_MOESM7_ESM.zip › Fig2/Fig2E/PARG +600s.tif]

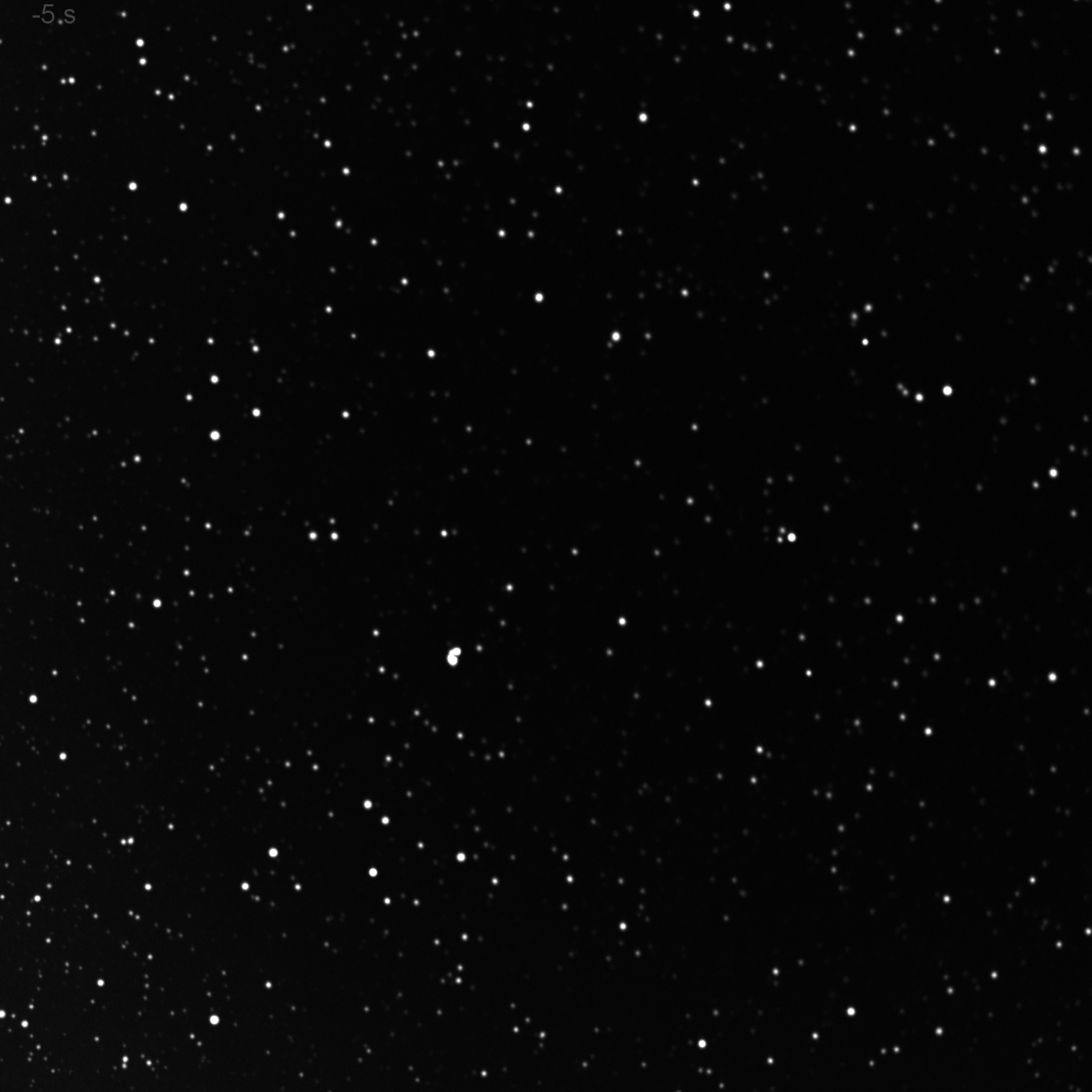

Supplement: Supplementary file 7 — Source data Fig. 2 [file 44319_2024_285_MOESM7_ESM.zip › Fig2/Fig2E/PARG -5s.tif]

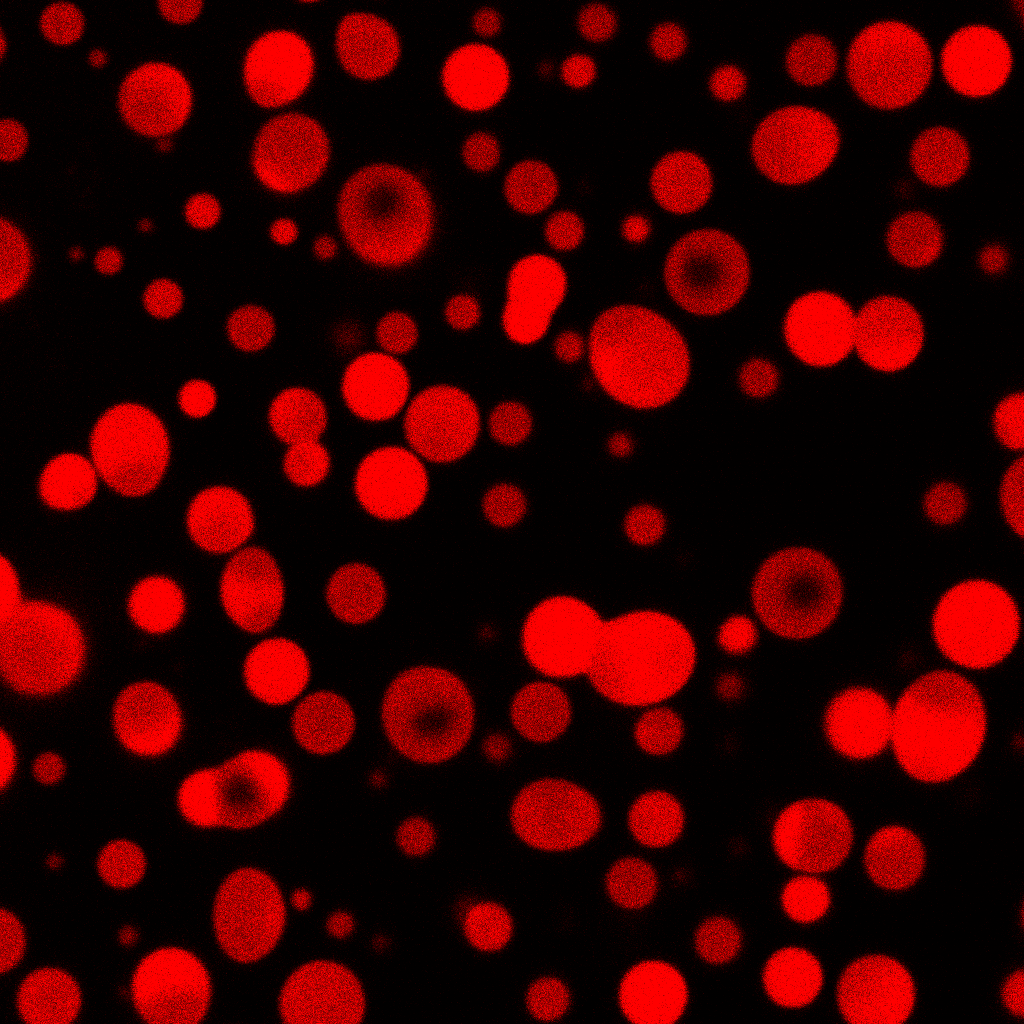

Supplement: Supplementary file 7 — Source data Fig. 2 [file 44319_2024_285_MOESM7_ESM.zip › Fig2/Fig2I/mChPARP1_NAD_fusion_t30.tif]

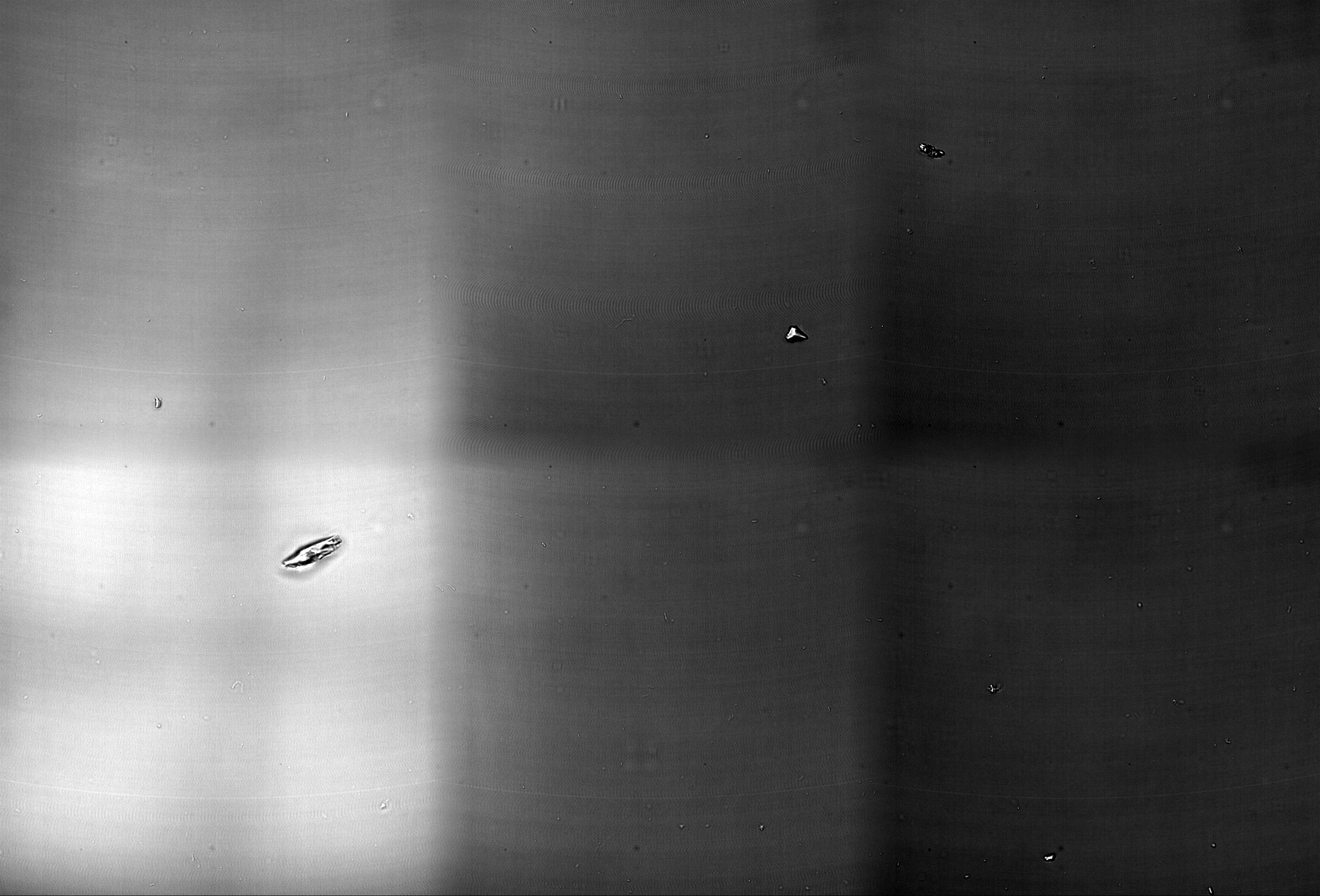

Supplement: Supplementary file 8 — Source data Fig. 3 [file 44319_2024_285_MOESM8_ESM.zip › Fig3/Fig3C/4uMZnF_NAD.tif]

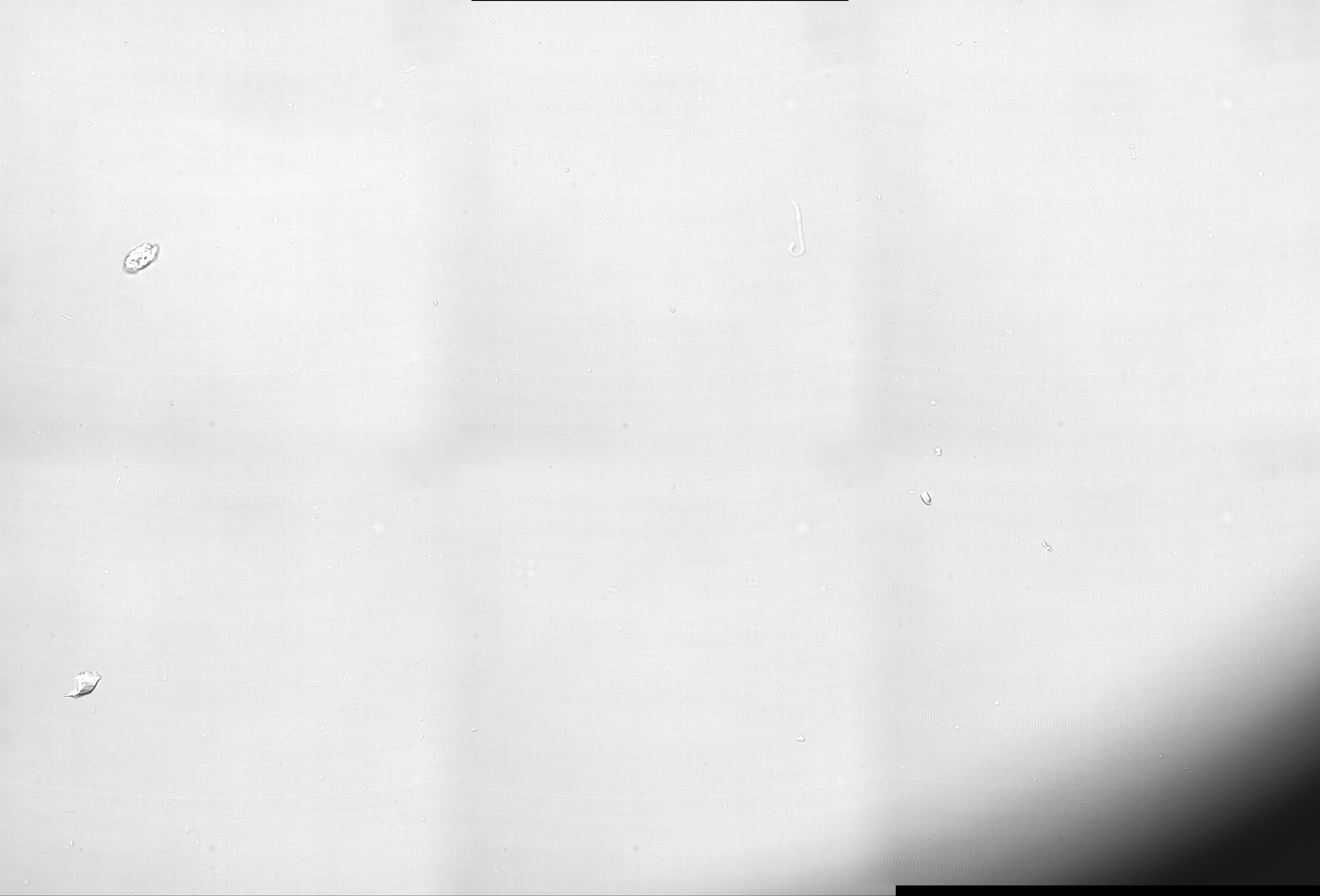

Supplement: Supplementary file 8 — Source data Fig. 3 [file 44319_2024_285_MOESM8_ESM.zip › Fig3/Fig3C/4uMZnF_noNAD.tif]

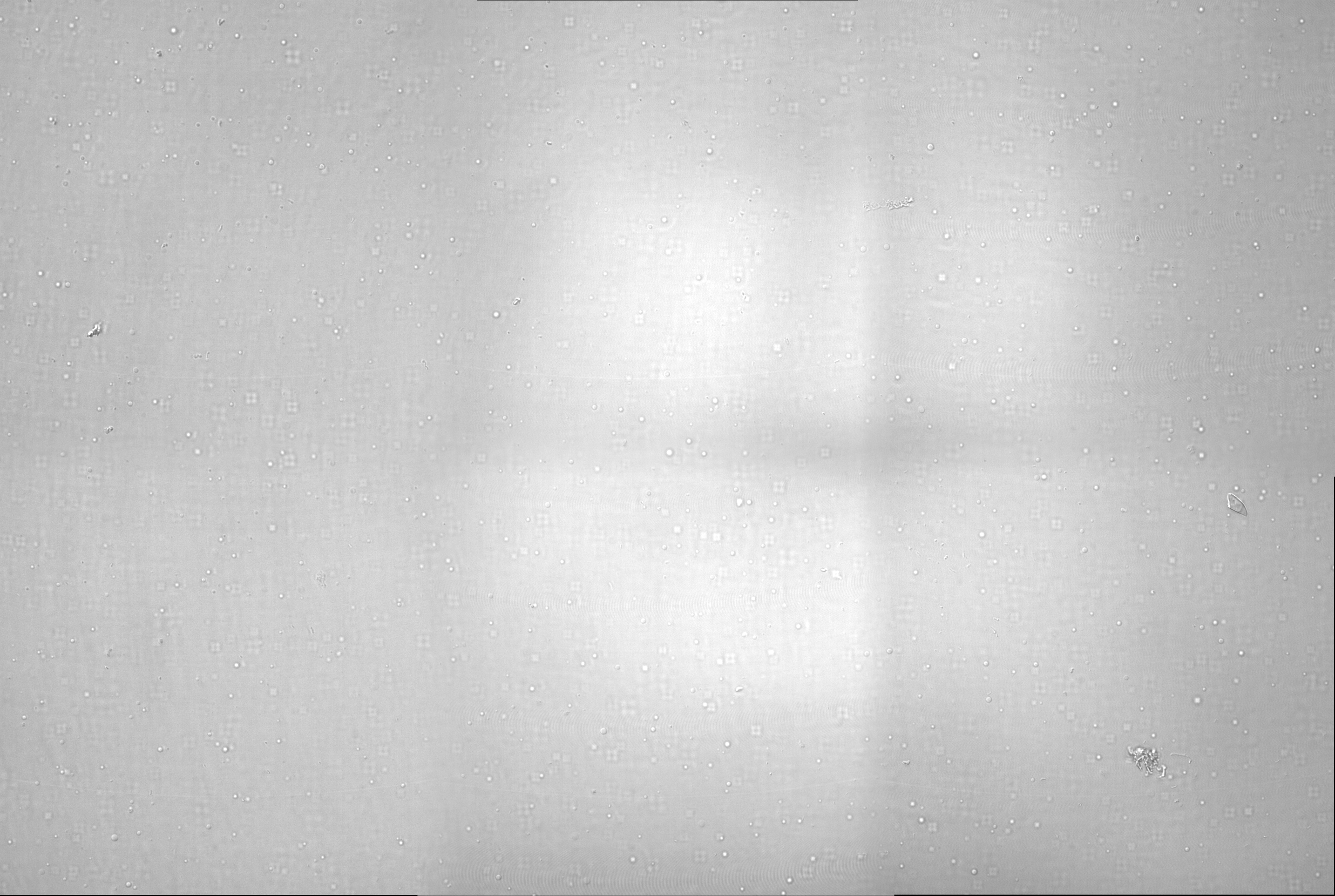

Supplement: Supplementary file 8 — Source data Fig. 3 [file 44319_2024_285_MOESM8_ESM.zip › Fig3/Fig3C/8uMZnF_NAD.tif]

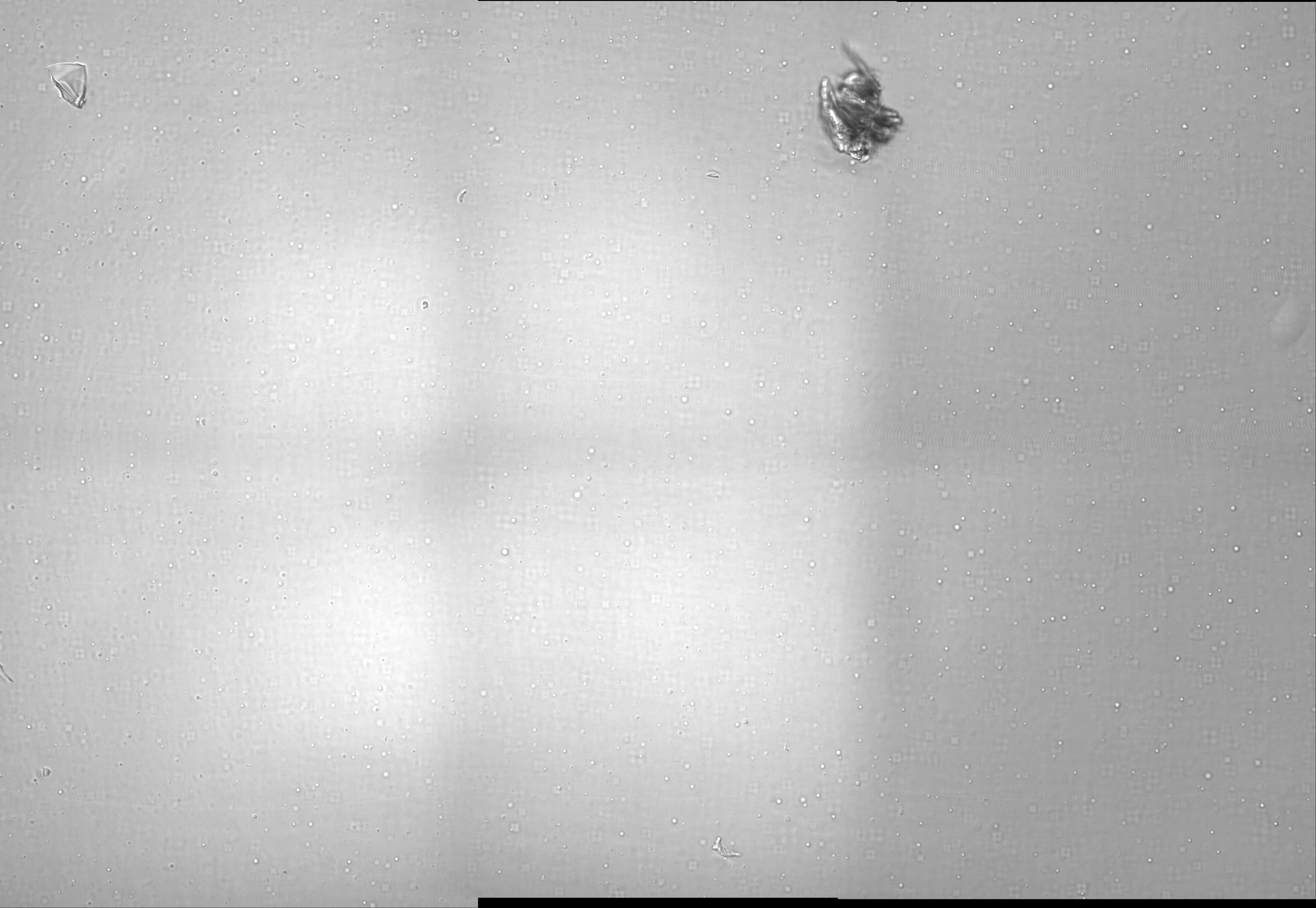

Supplement: Supplementary file 8 — Source data Fig. 3 [file 44319_2024_285_MOESM8_ESM.zip › Fig3/Fig3C/8uMZnF_noNAD.tif]

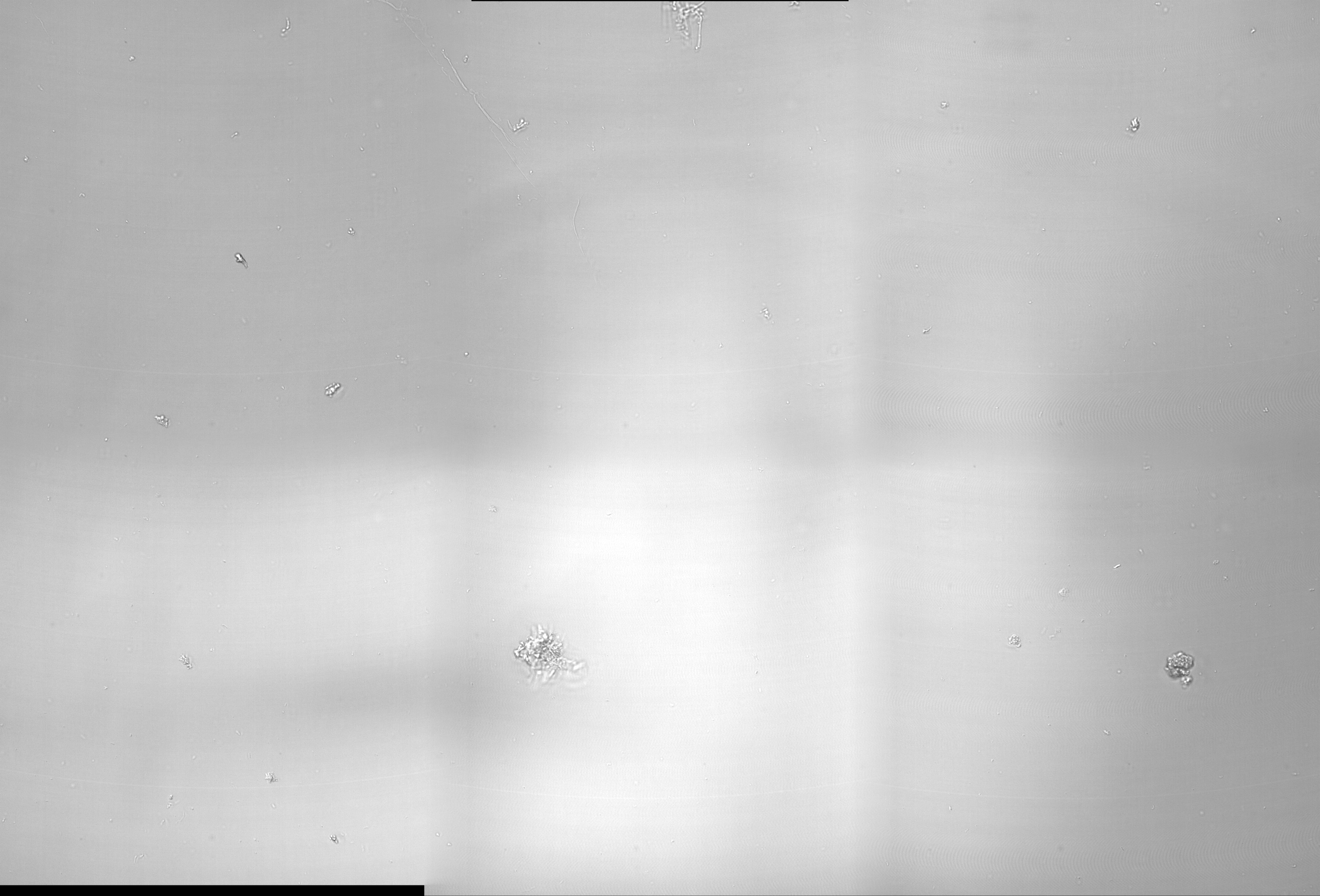

Supplement: Supplementary file 8 — Source data Fig. 3 [file 44319_2024_285_MOESM8_ESM.zip › Fig3/Fig3D/2uMZnF_2uMdZnF_NAD.tif]

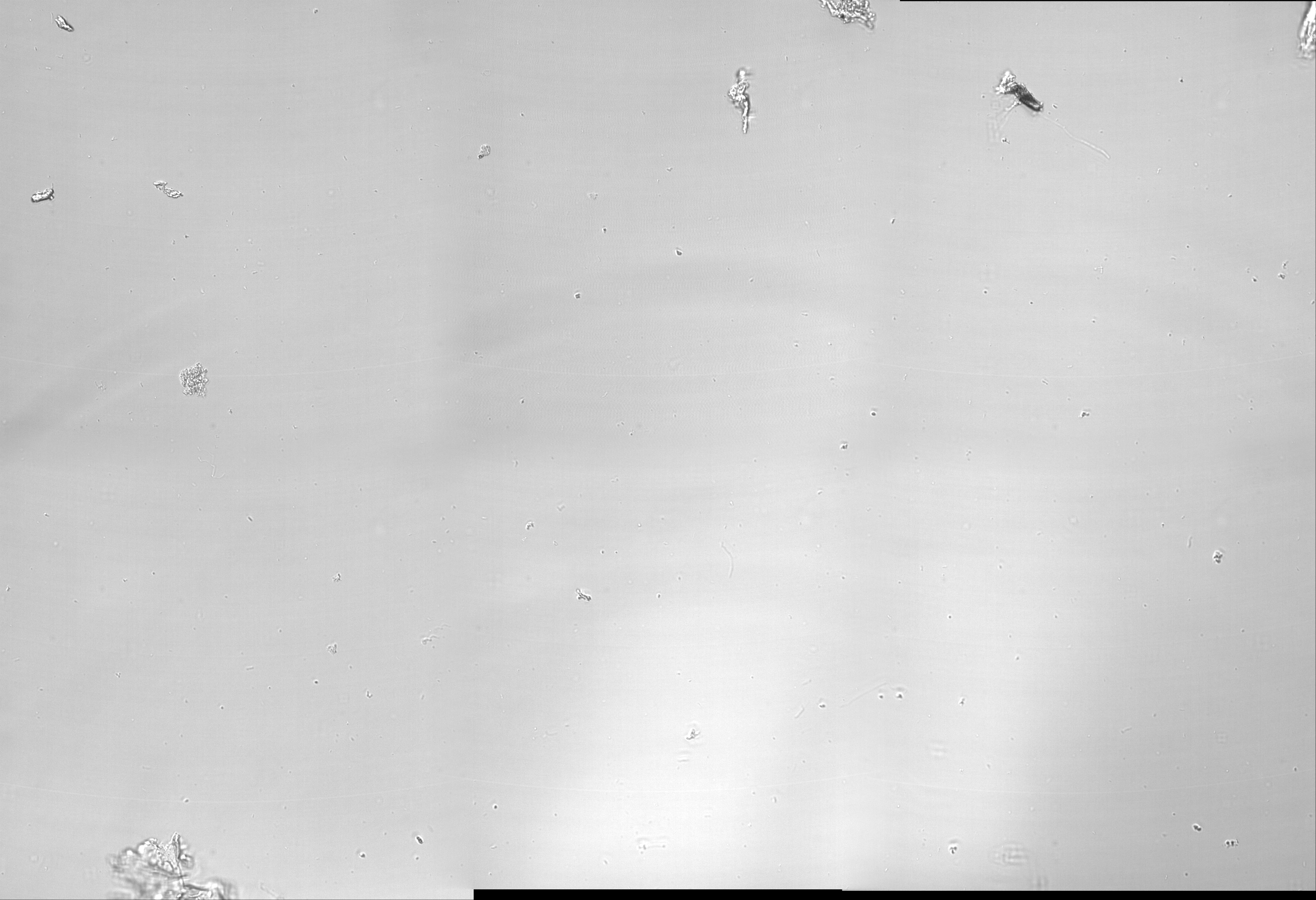

Supplement: Supplementary file 8 — Source data Fig. 3 [file 44319_2024_285_MOESM8_ESM.zip › Fig3/Fig3D/2uMZnF_2uMdZnF_noNAD.tif]

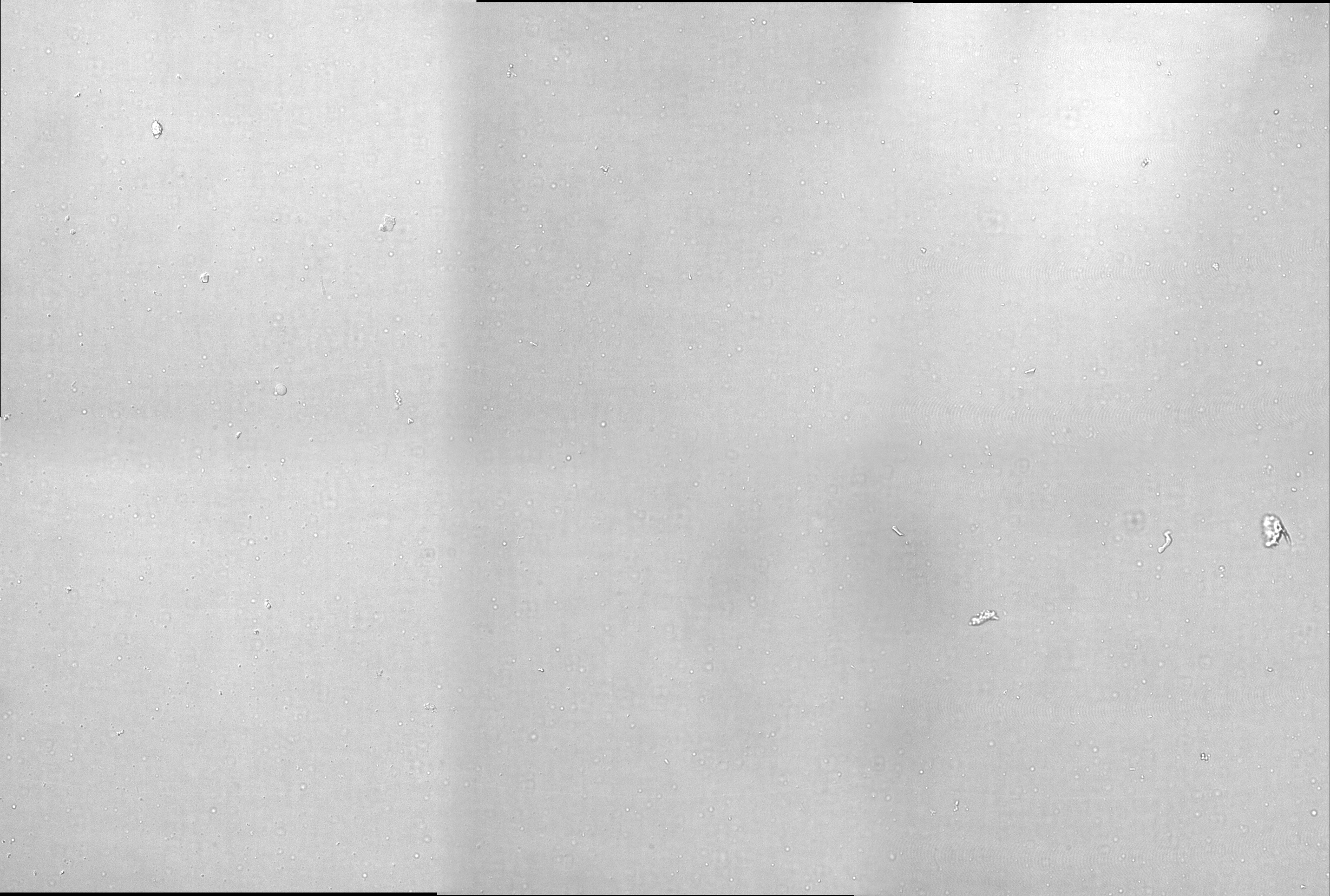

Supplement: Supplementary file 8 — Source data Fig. 3 [file 44319_2024_285_MOESM8_ESM.zip › Fig3/Fig3D/4uMZnF_4uMdZnF_NAD.tif]

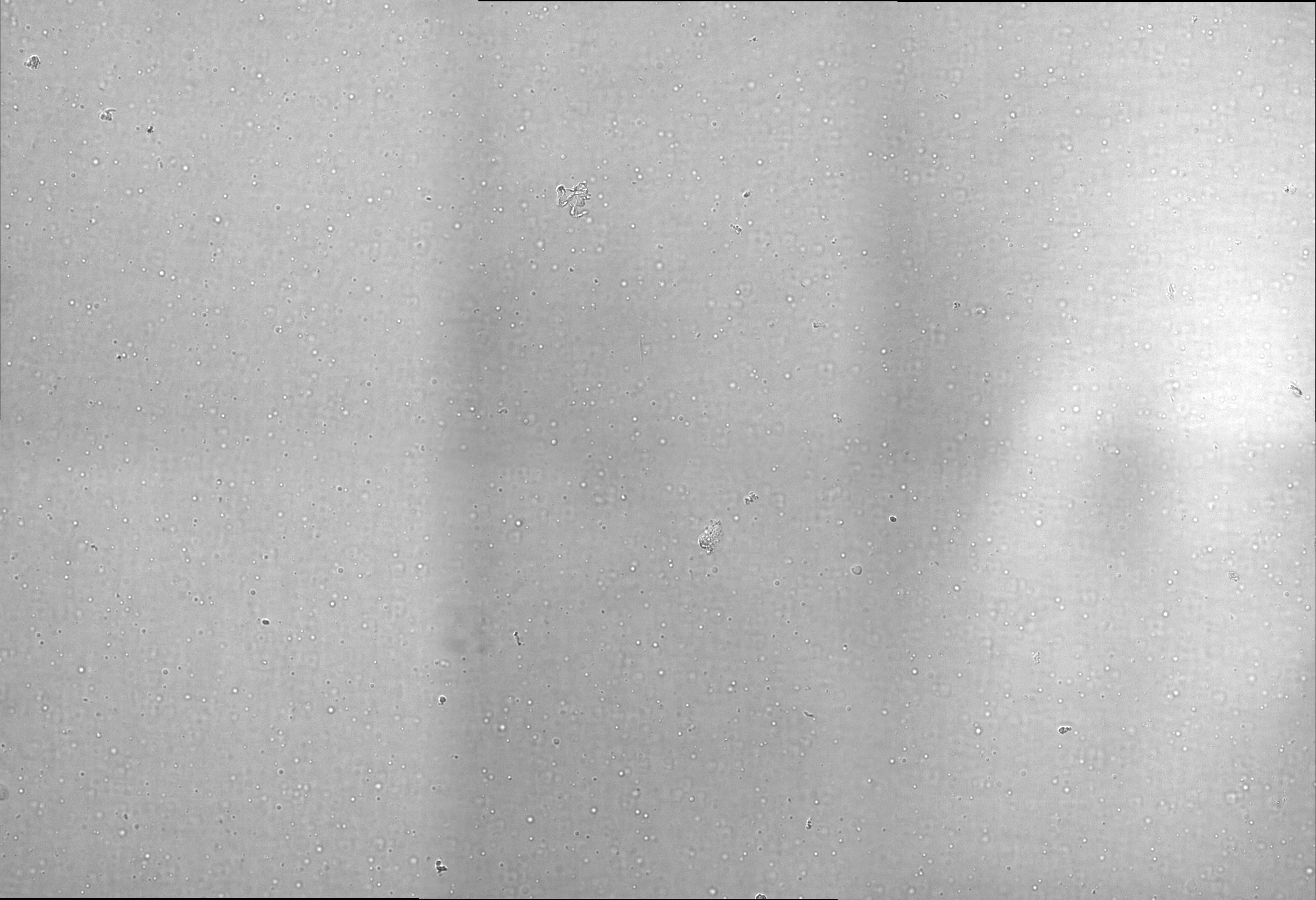

Supplement: Supplementary file 8 — Source data Fig. 3 [file 44319_2024_285_MOESM8_ESM.zip › Fig3/Fig3D/4uMZnF_4uMdZnF_noNAD.tif]

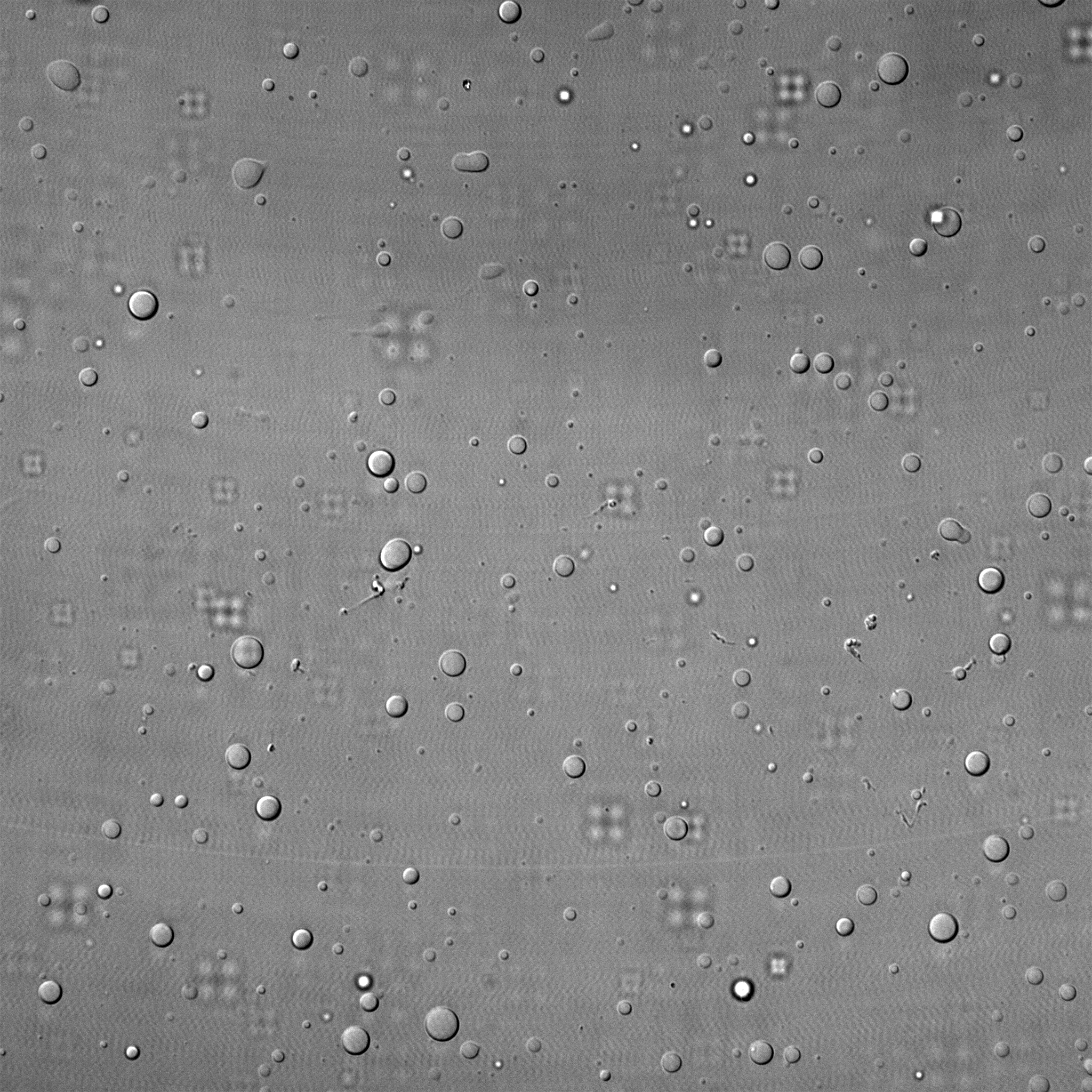

Supplement: Supplementary file 8 — Source data Fig. 3 [file 44319_2024_285_MOESM8_ESM.zip › Fig3/Fig3E/1uM PARP1 with NAD.tif]

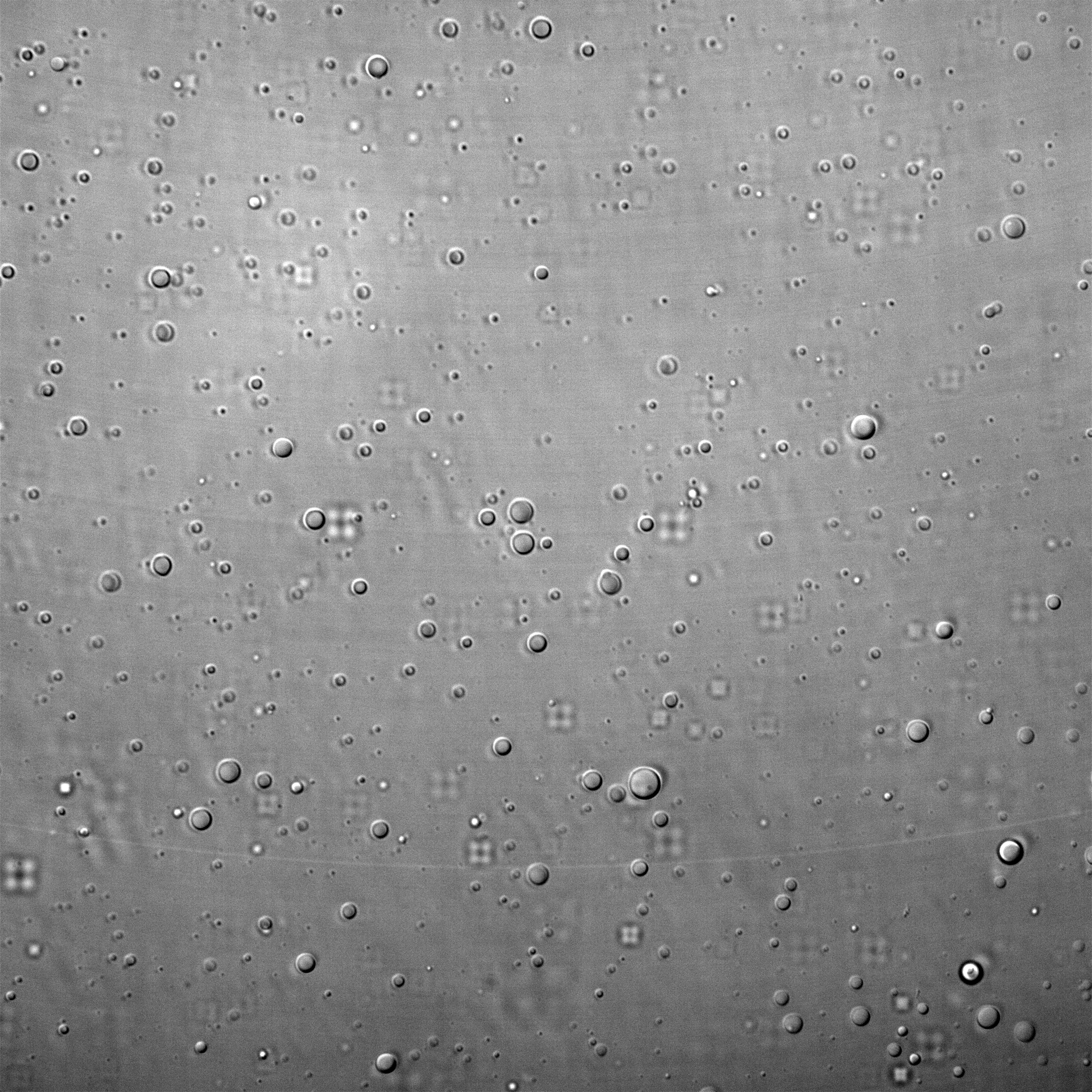

Supplement: Supplementary file 8 — Source data Fig. 3 [file 44319_2024_285_MOESM8_ESM.zip › Fig3/Fig3E/1uM PARP1 without NAD.tif]

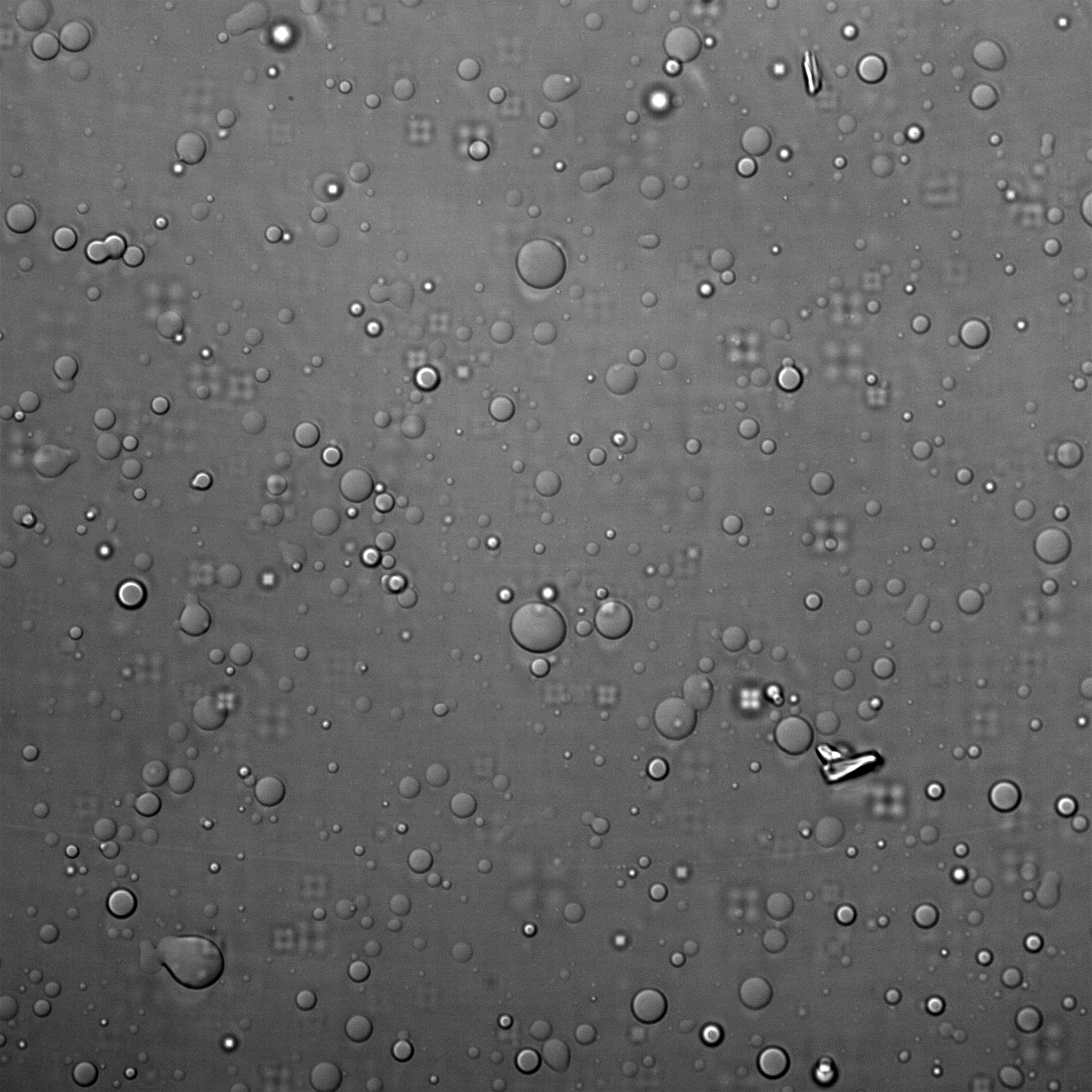

Supplement: Supplementary file 8 — Source data Fig. 3 [file 44319_2024_285_MOESM8_ESM.zip › Fig3/Fig3E/2uM PARP1 with NAD.tif]

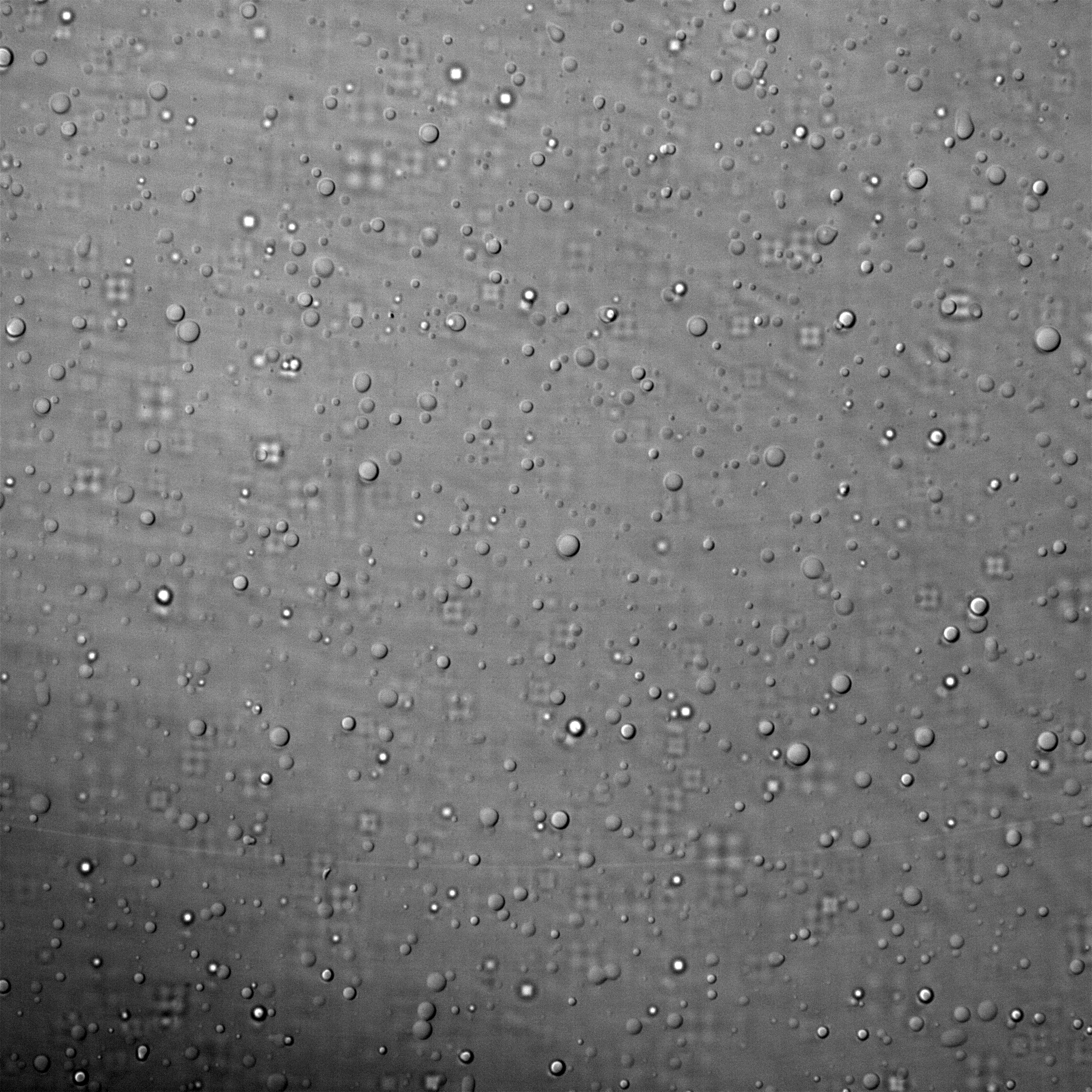

Supplement: Supplementary file 8 — Source data Fig. 3 [file 44319_2024_285_MOESM8_ESM.zip › Fig3/Fig3E/2uM PARP1 without NAD.tif]

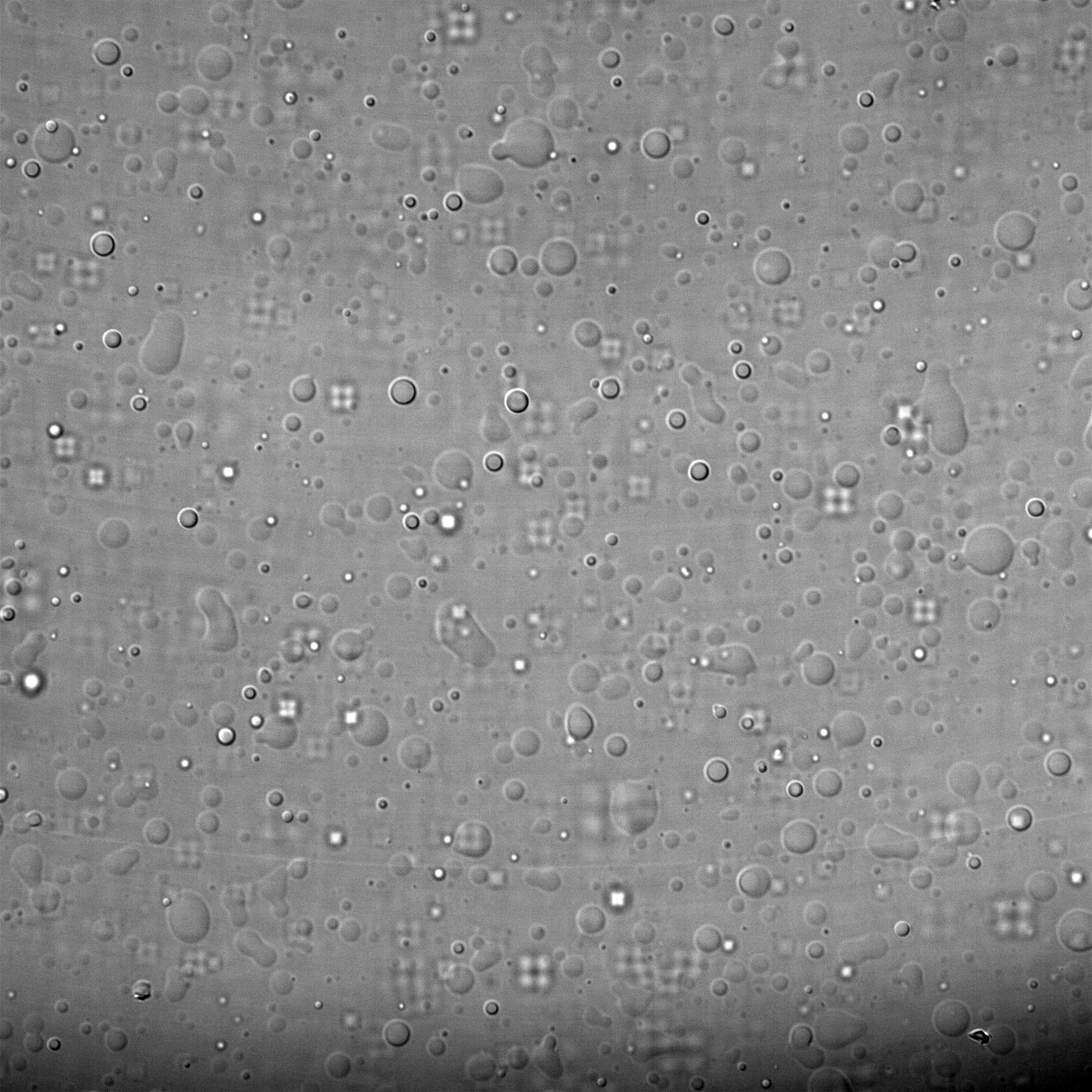

Supplement: Supplementary file 8 — Source data Fig. 3 [file 44319_2024_285_MOESM8_ESM.zip › Fig3/Fig3E/4uM PARP1 with NAD.tif]

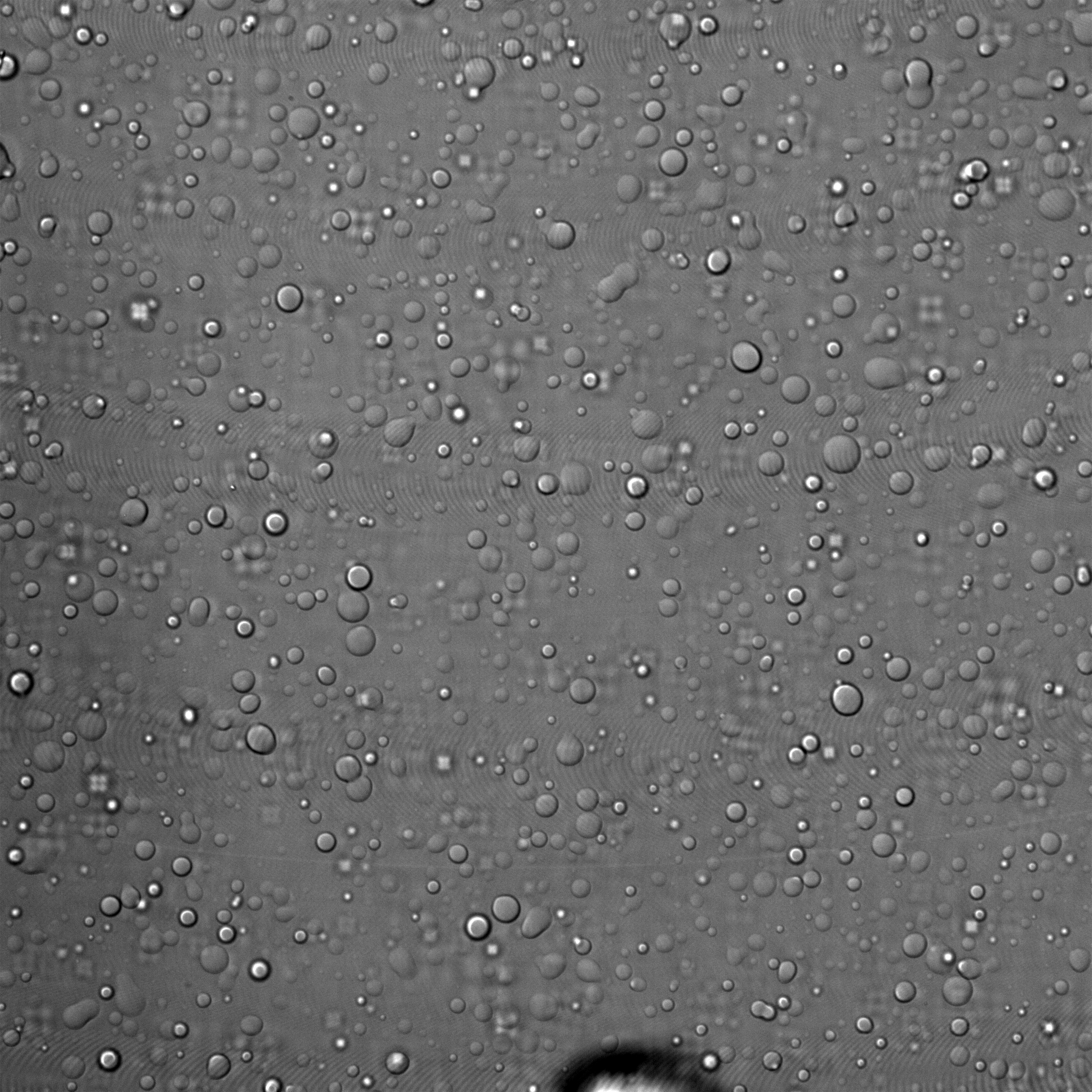

Supplement: Supplementary file 8 — Source data Fig. 3 [file 44319_2024_285_MOESM8_ESM.zip › Fig3/Fig3E/4uM PARP1 without NAD.tif]

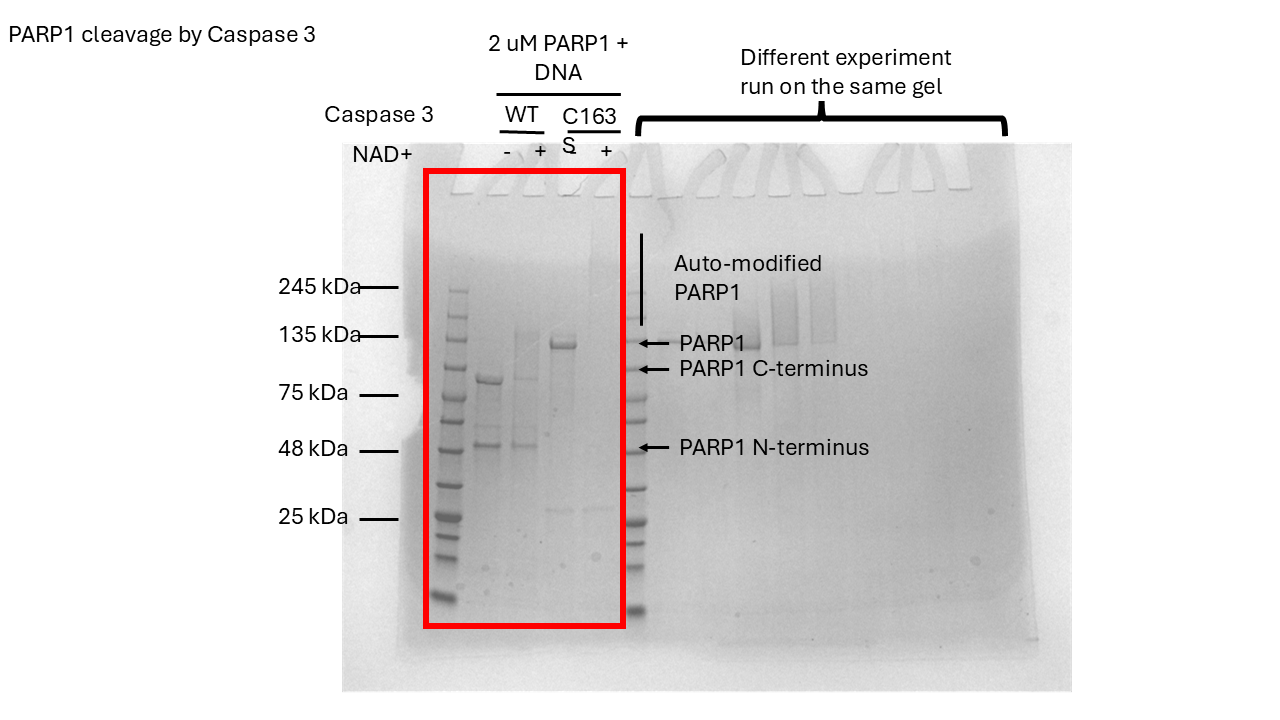

Supplement: Supplementary file 8 — Source data Fig. 3 [file 44319_2024_285_MOESM8_ESM.zip › Fig3/Fig3G/Caspase_Cleavage_Gel.tif]

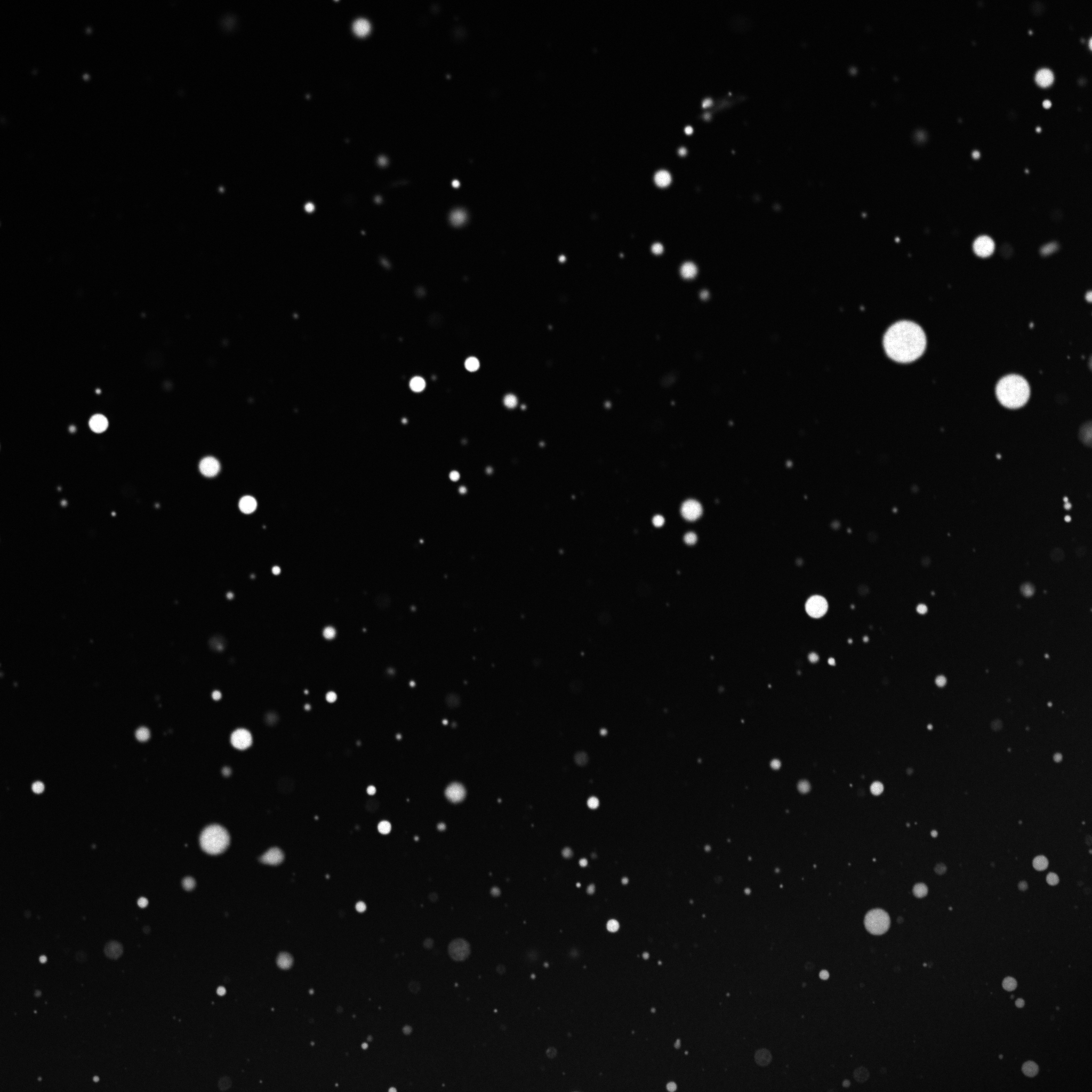

Supplement: Supplementary file 8 — Source data Fig. 3 [file 44319_2024_285_MOESM8_ESM.zip › Fig3/Fig3H/Caspase-3 C165S with NAD.tif]

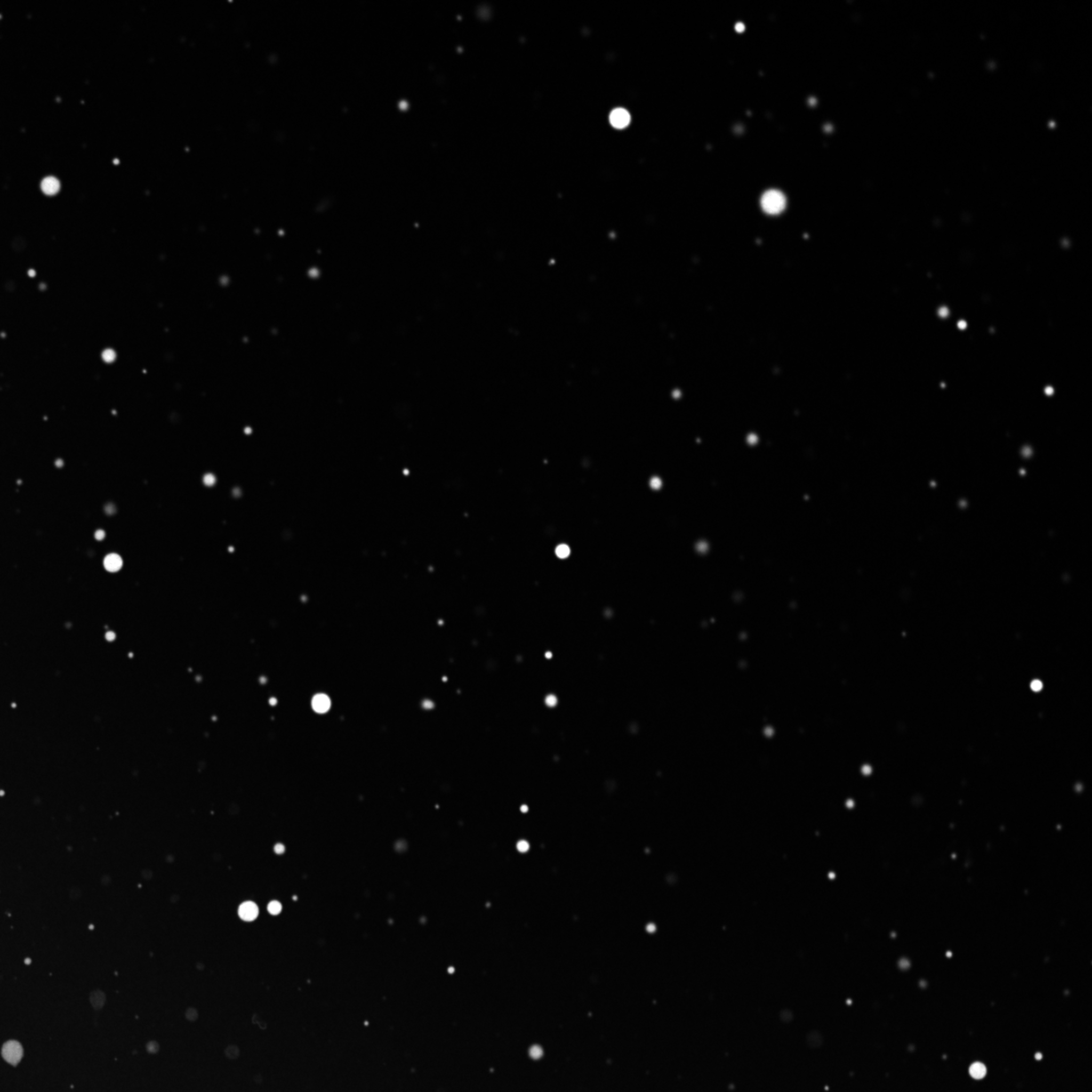

Supplement: Supplementary file 8 — Source data Fig. 3 [file 44319_2024_285_MOESM8_ESM.zip › Fig3/Fig3H/Caspase-3 C165S without NAD.tif]

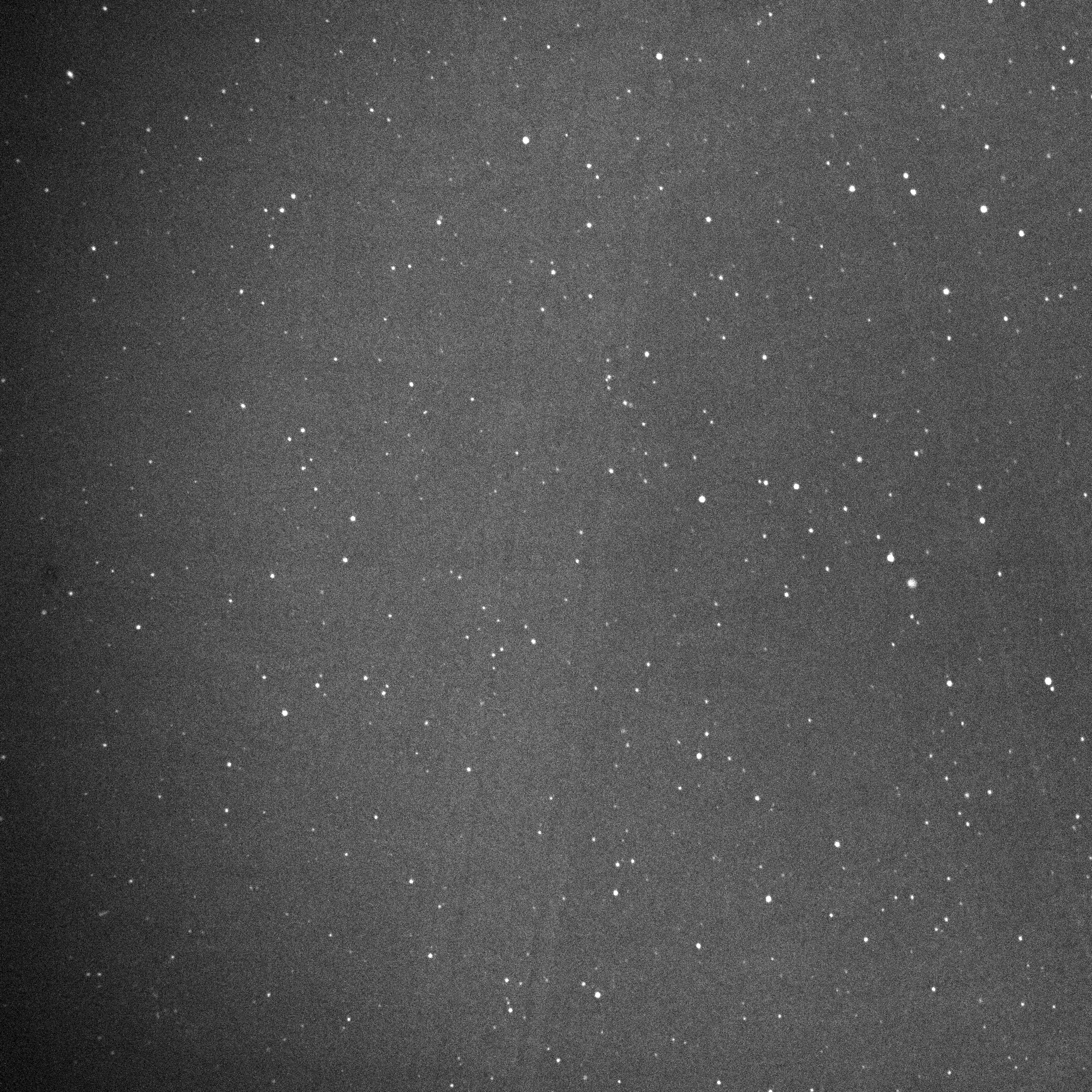

Supplement: Supplementary file 8 — Source data Fig. 3 [file 44319_2024_285_MOESM8_ESM.zip › Fig3/Fig3H/Caspase-3 WT with NAD.tif]

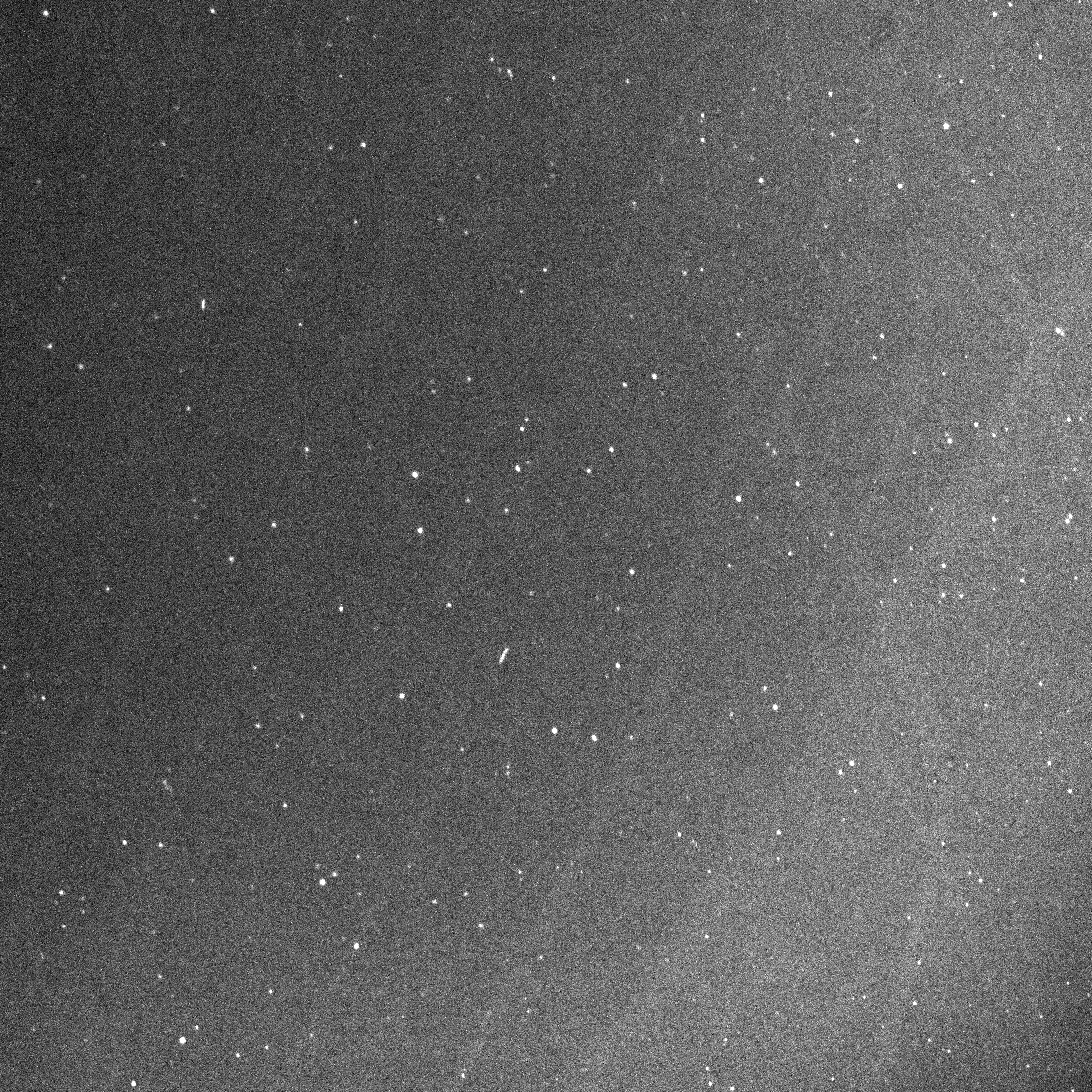

Supplement: Supplementary file 8 — Source data Fig. 3 [file 44319_2024_285_MOESM8_ESM.zip › Fig3/Fig3H/Caspase-3 WT without NAD.tif]

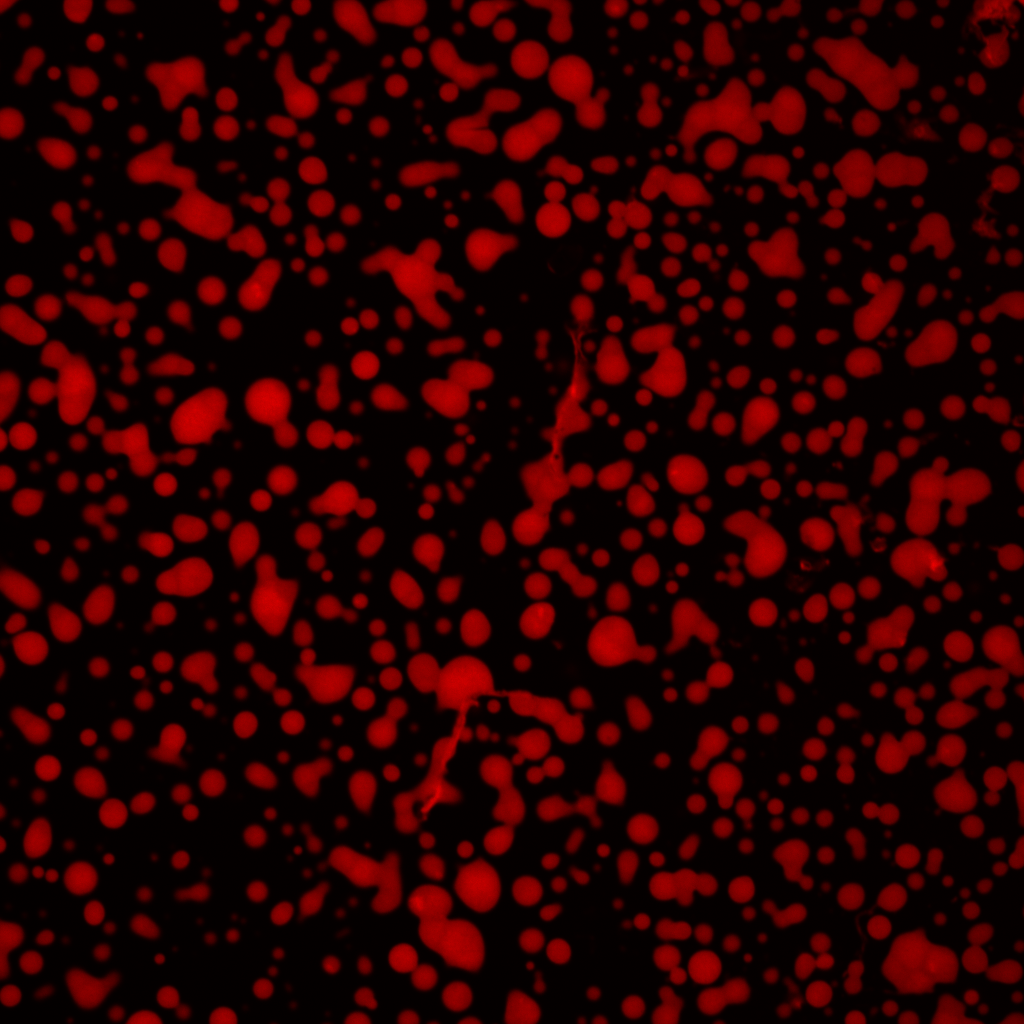

Supplement: Supplementary file 9 — Source data Fig. 4 [file 44319_2024_285_MOESM9_ESM.zip › Fig4/Fig4A/DNA_Partitioning_NAD_Cy5TriplexDNA.tif]

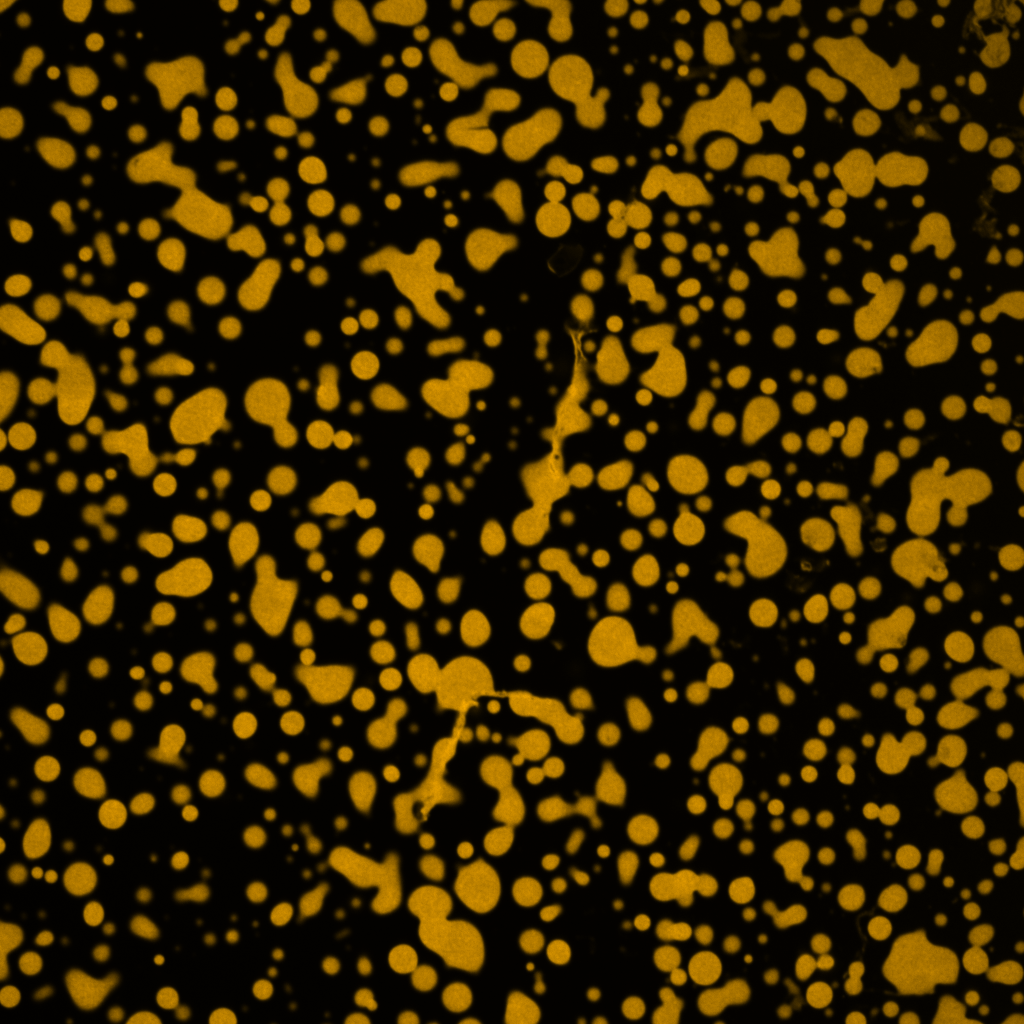

Supplement: Supplementary file 9 — Source data Fig. 4 [file 44319_2024_285_MOESM9_ESM.zip › Fig4/Fig4A/DNA_Partitioning_NAD_mChPARP1.tif]

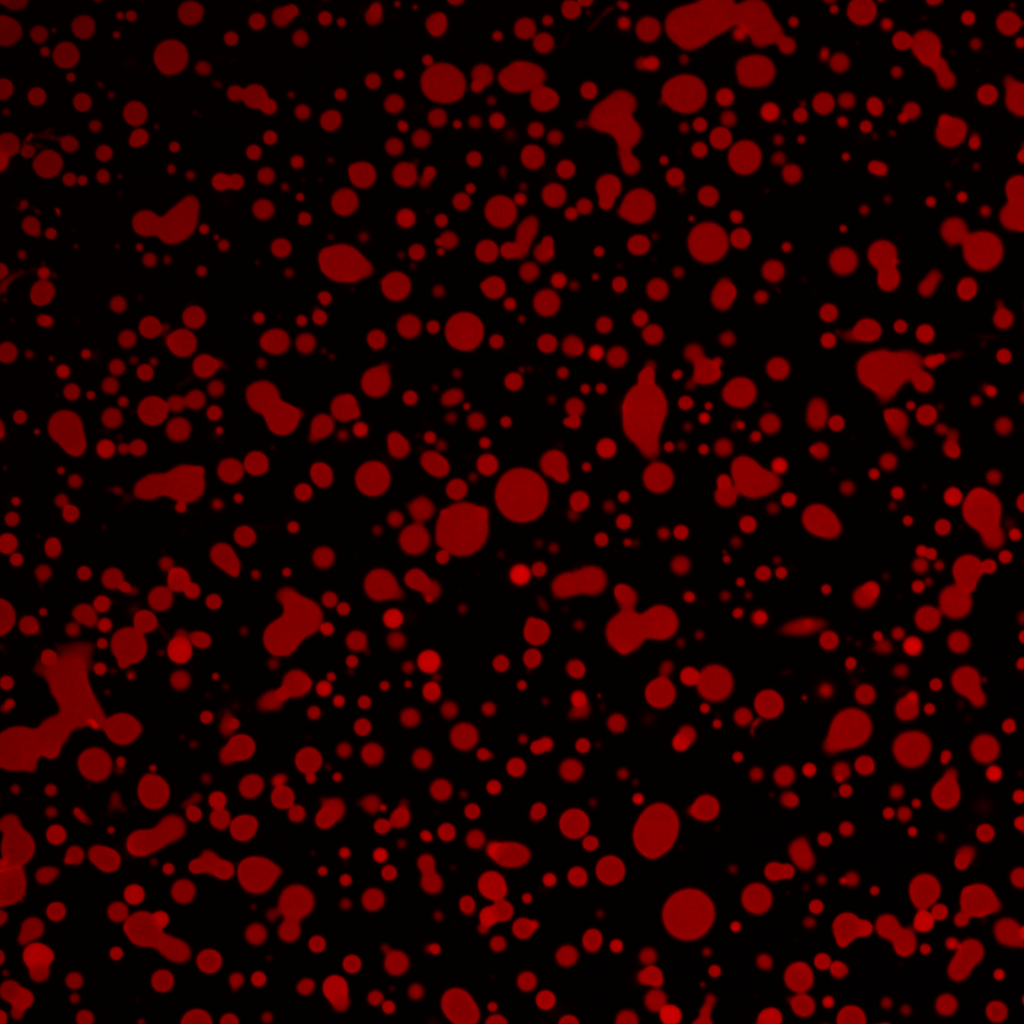

Supplement: Supplementary file 9 — Source data Fig. 4 [file 44319_2024_285_MOESM9_ESM.zip › Fig4/Fig4A/DNA_Partitioning_noNAD_Cy5TriplexDNA.tif]

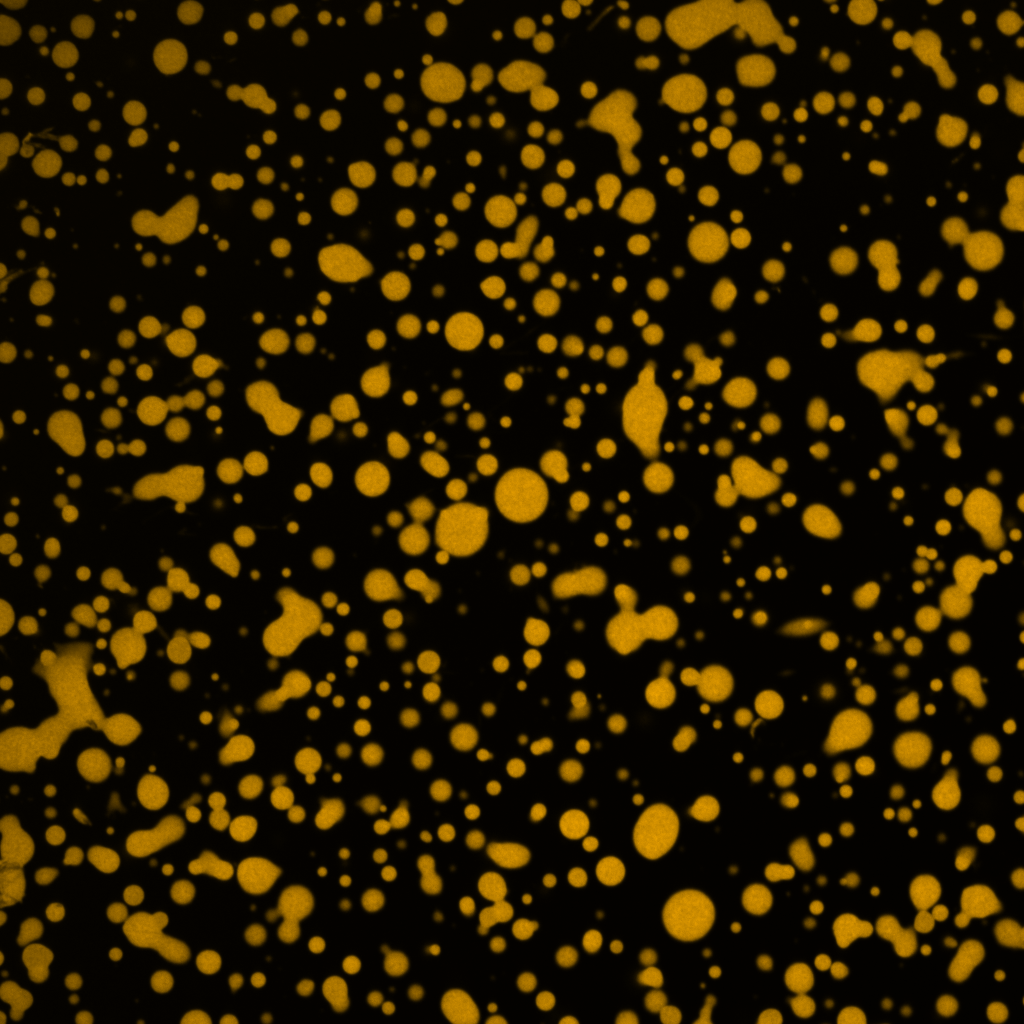

Supplement: Supplementary file 9 — Source data Fig. 4 [file 44319_2024_285_MOESM9_ESM.zip › Fig4/Fig4A/DNA_Partitioning_noNAD_mChPARP1.tif]

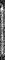

Supplement: Supplementary file 9 — Source data Fig. 4 [file 44319_2024_285_MOESM9_ESM.zip › Fig4/Fig4C/PARP1.tif]

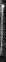

Supplement: Supplementary file 9 — Source data Fig. 4 [file 44319_2024_285_MOESM9_ESM.zip › Fig4/Fig4C/PARP1-E998Q.tif]

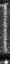

Supplement: Supplementary file 9 — Source data Fig. 4 [file 44319_2024_285_MOESM9_ESM.zip › Fig4/Fig4E/1 mM NAD+.tif]

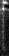

Supplement: Supplementary file 9 — Source data Fig. 4 [file 44319_2024_285_MOESM9_ESM.zip › Fig4/Fig4E/100 uM NAD+.tif]

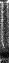

Supplement: Supplementary file 9 — Source data Fig. 4 [file 44319_2024_285_MOESM9_ESM.zip › Fig4/Fig4E/500 uM NAD+.tif]

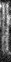

Supplement: Supplementary file 9 — Source data Fig. 4 [file 44319_2024_285_MOESM9_ESM.zip › Fig4/Fig4G/end_bridging_resolving.tif]

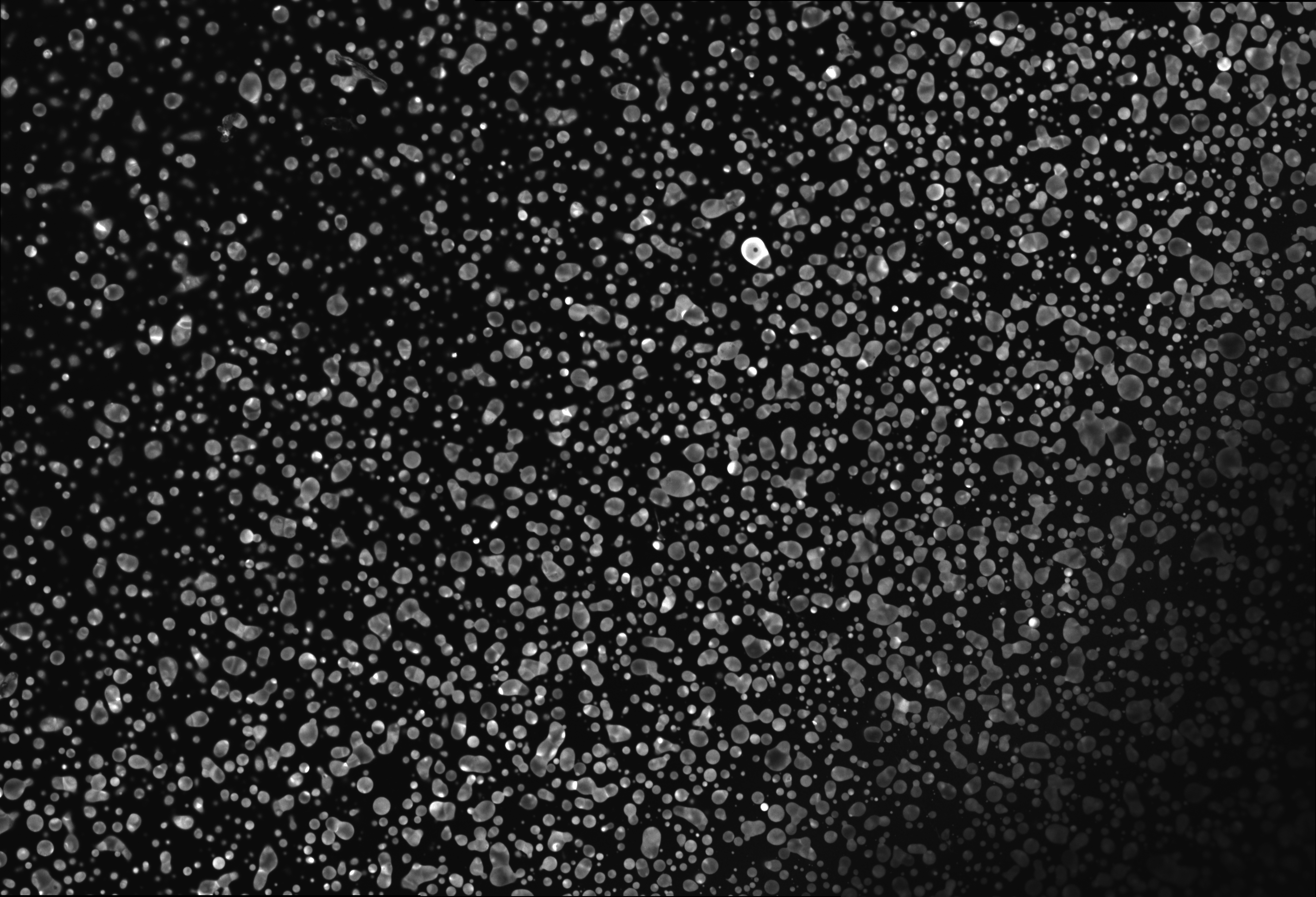

Supplement: Supplementary file 10 — Source data Fig. 5 [file 44319_2024_285_MOESM10_ESM.zip › Fig5/Fig5A/LIG3_Partitioning_NAD.tif]

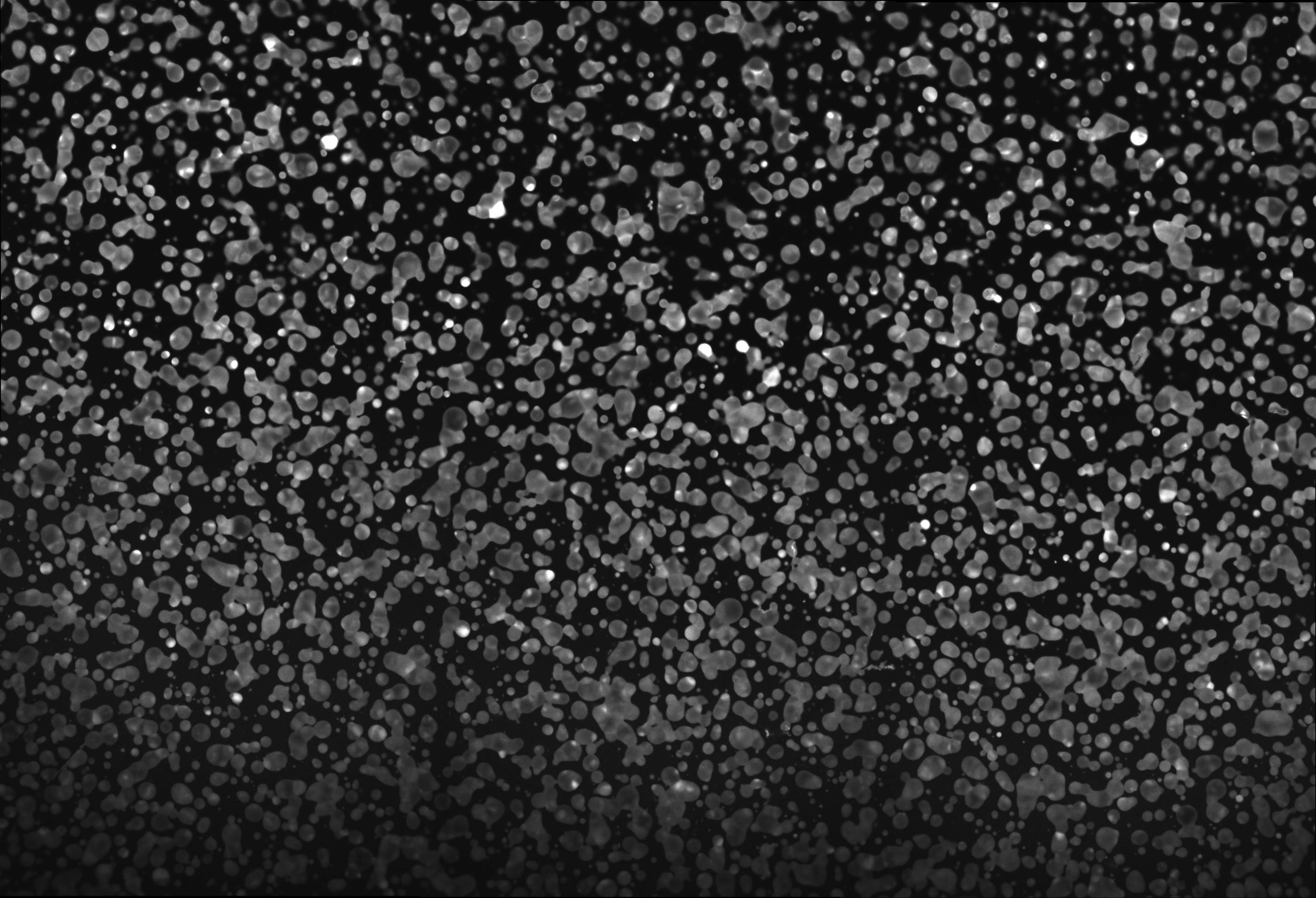

Supplement: Supplementary file 10 — Source data Fig. 5 [file 44319_2024_285_MOESM10_ESM.zip › Fig5/Fig5A/LIG3_Partitioning_noNAD.tif]

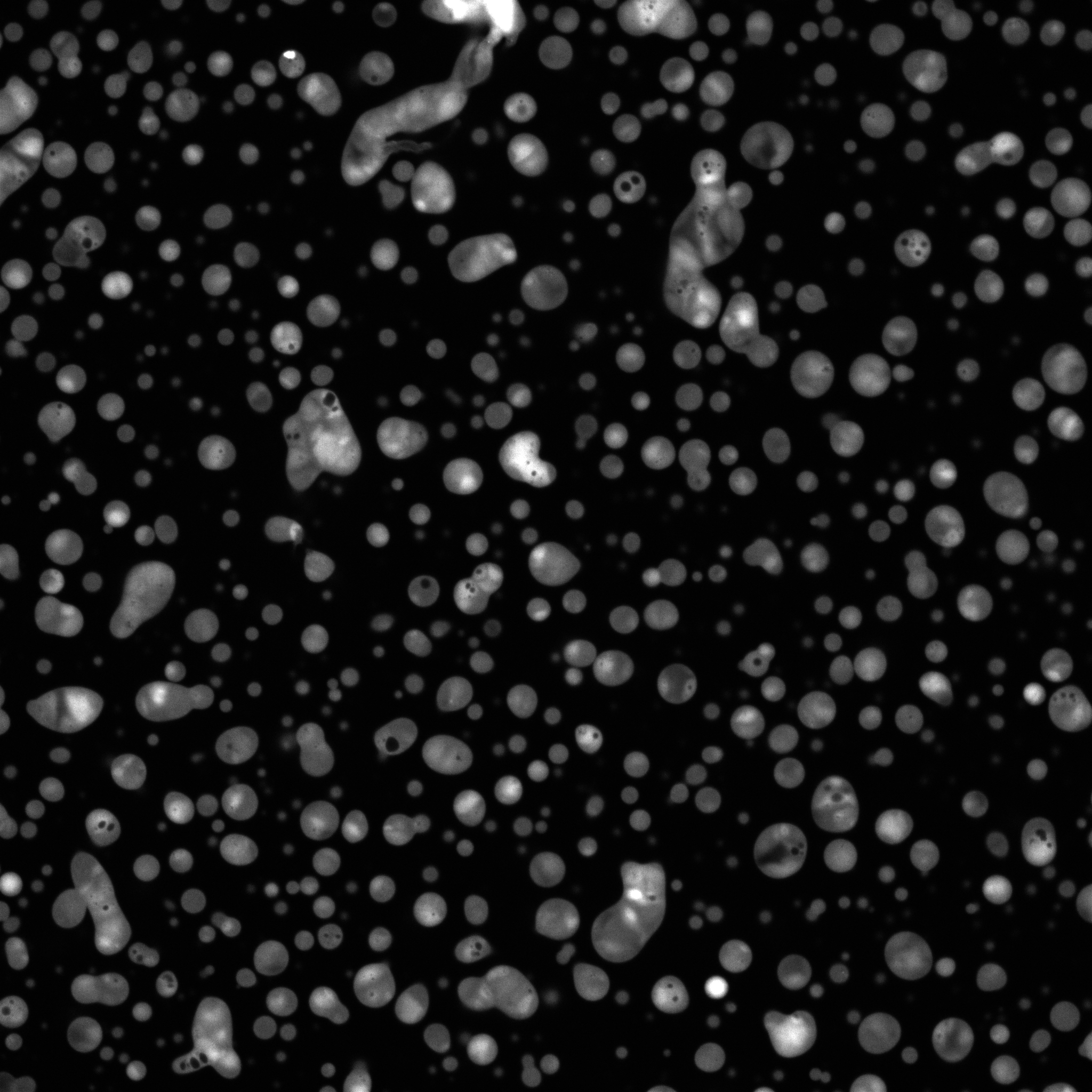

Supplement: Supplementary file 10 — Source data Fig. 5 [file 44319_2024_285_MOESM10_ESM.zip › Fig5/Fig5D/With NAD all channels.tif]

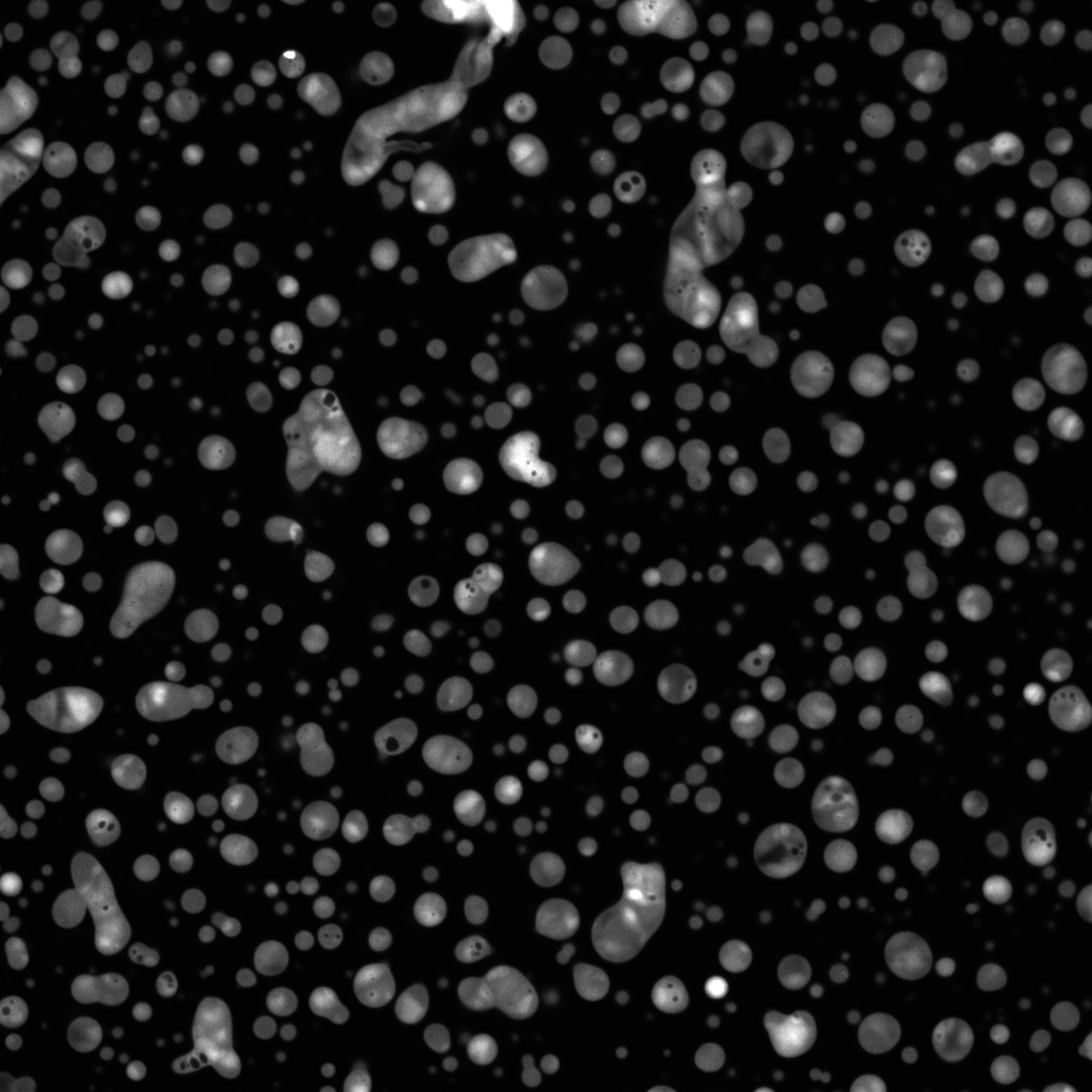

Supplement: Supplementary file 10 — Source data Fig. 5 [file 44319_2024_285_MOESM10_ESM.zip › Fig5/Fig5D/With NAD Lig3 channel.tif]

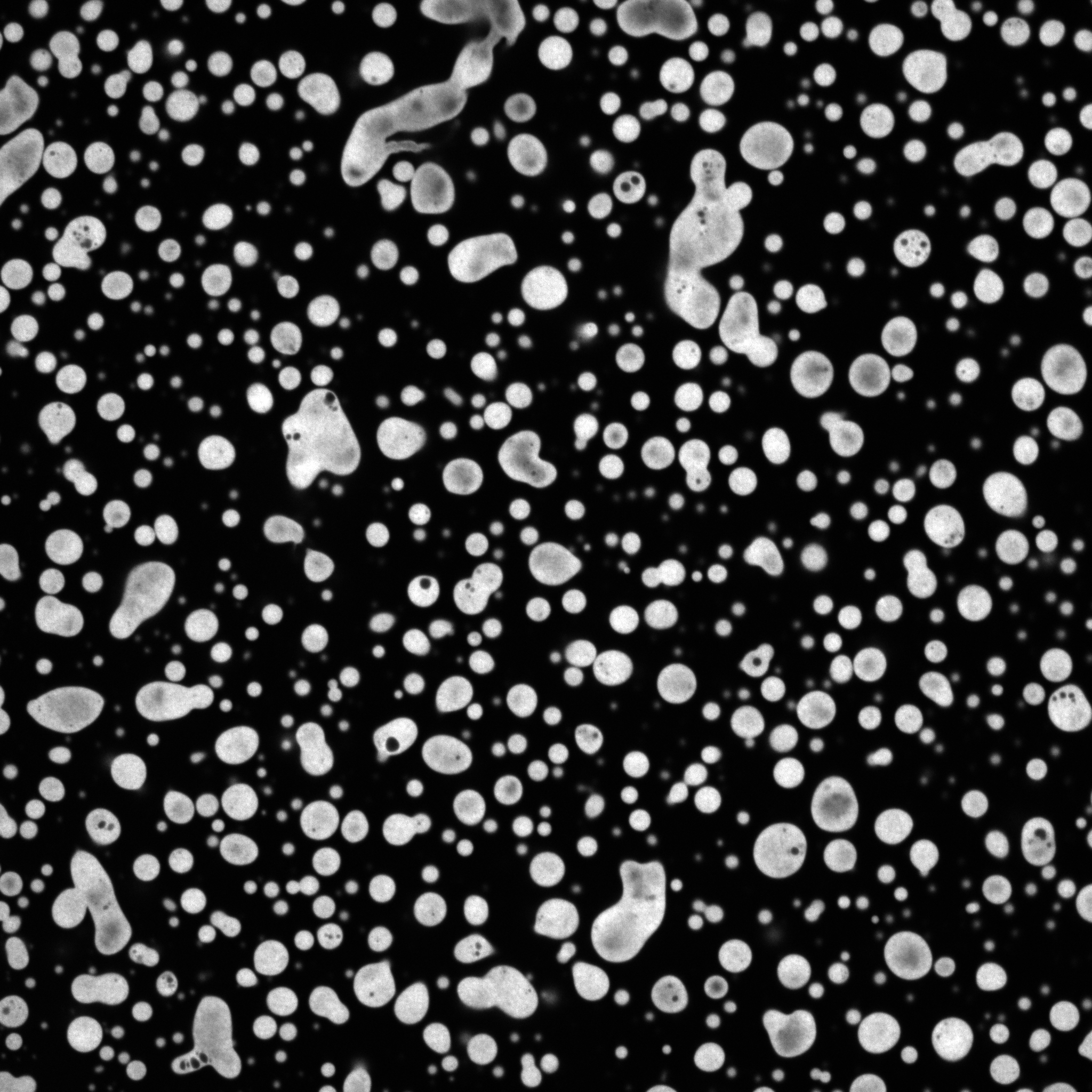

Supplement: Supplementary file 10 — Source data Fig. 5 [file 44319_2024_285_MOESM10_ESM.zip › Fig5/Fig5D/With NAD mCh-PARP1 channel.tif]

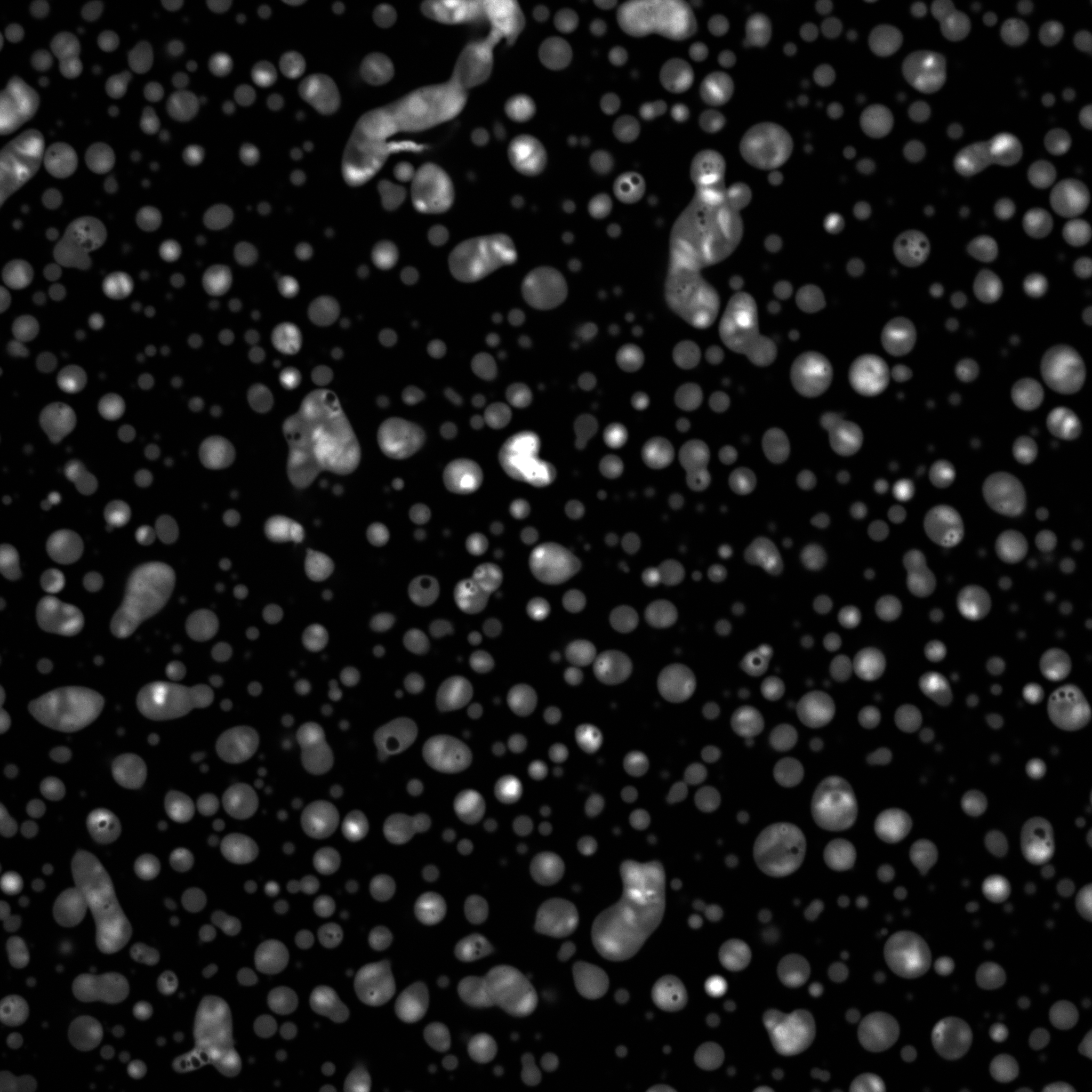

Supplement: Supplementary file 10 — Source data Fig. 5 [file 44319_2024_285_MOESM10_ESM.zip › Fig5/Fig5D/With NAD Triplex DNA channel.tif]

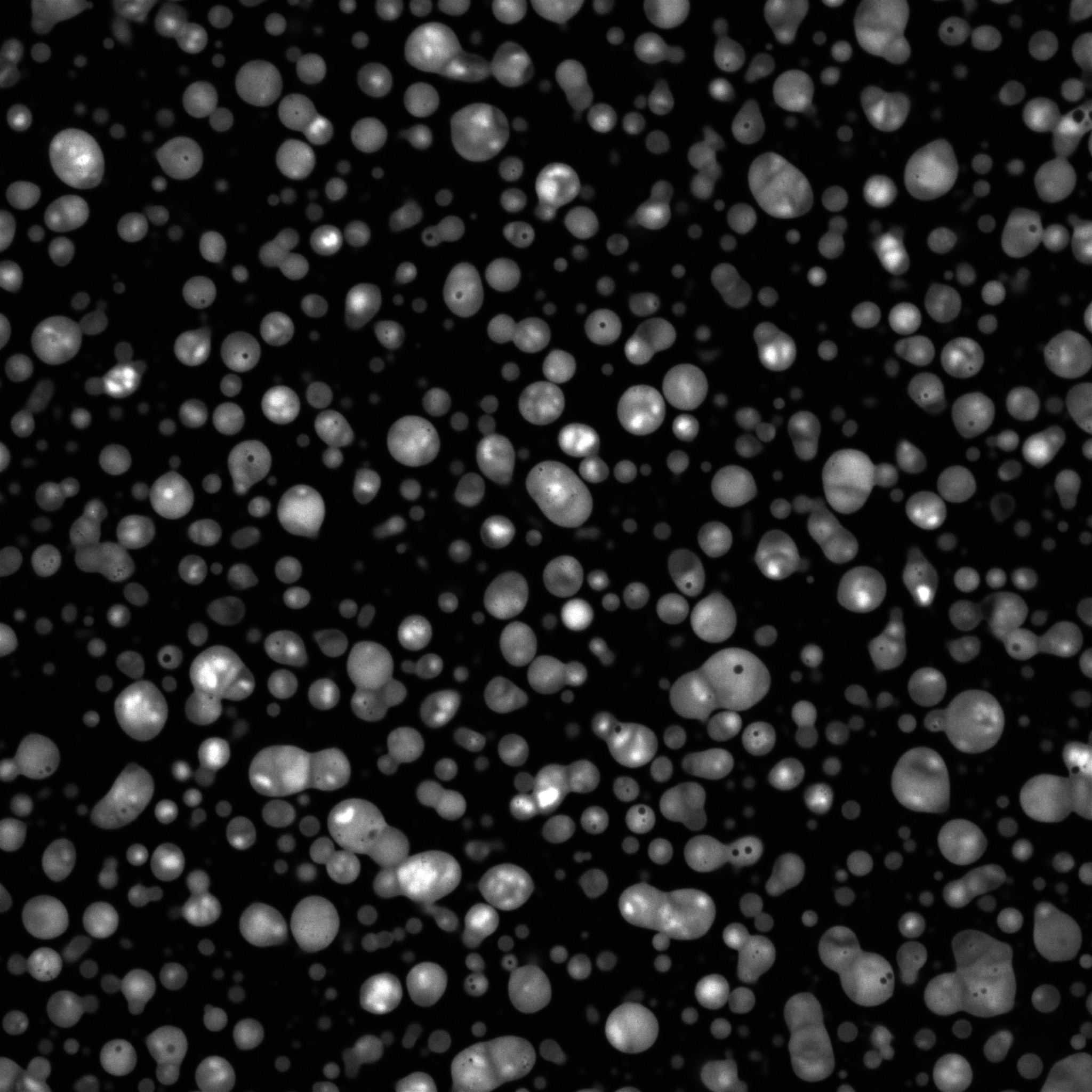

Supplement: Supplementary file 10 — Source data Fig. 5 [file 44319_2024_285_MOESM10_ESM.zip › Fig5/Fig5D/Without NAD all channels.tif]

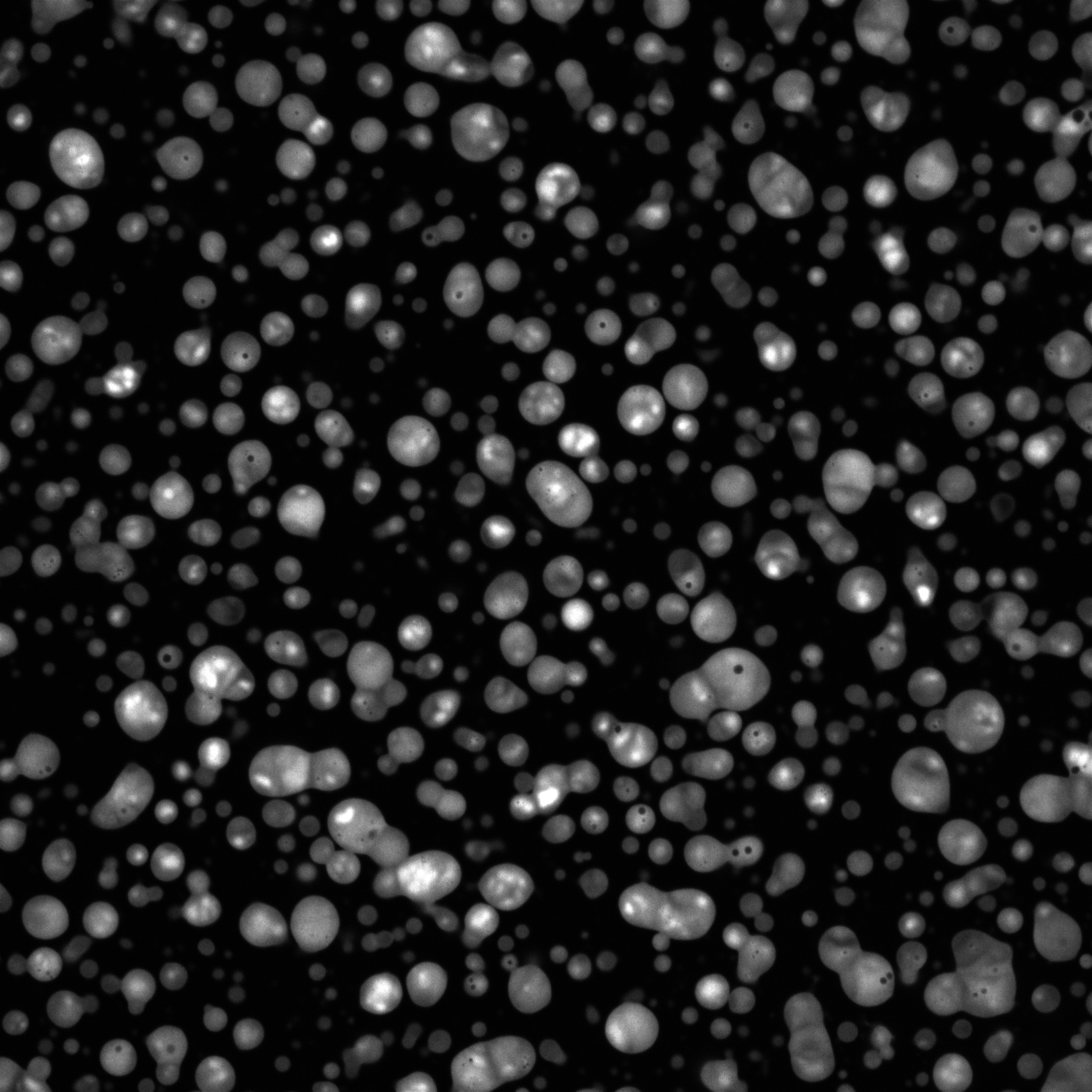

Supplement: Supplementary file 10 — Source data Fig. 5 [file 44319_2024_285_MOESM10_ESM.zip › Fig5/Fig5D/Without NAD Lig3 channel.tif]

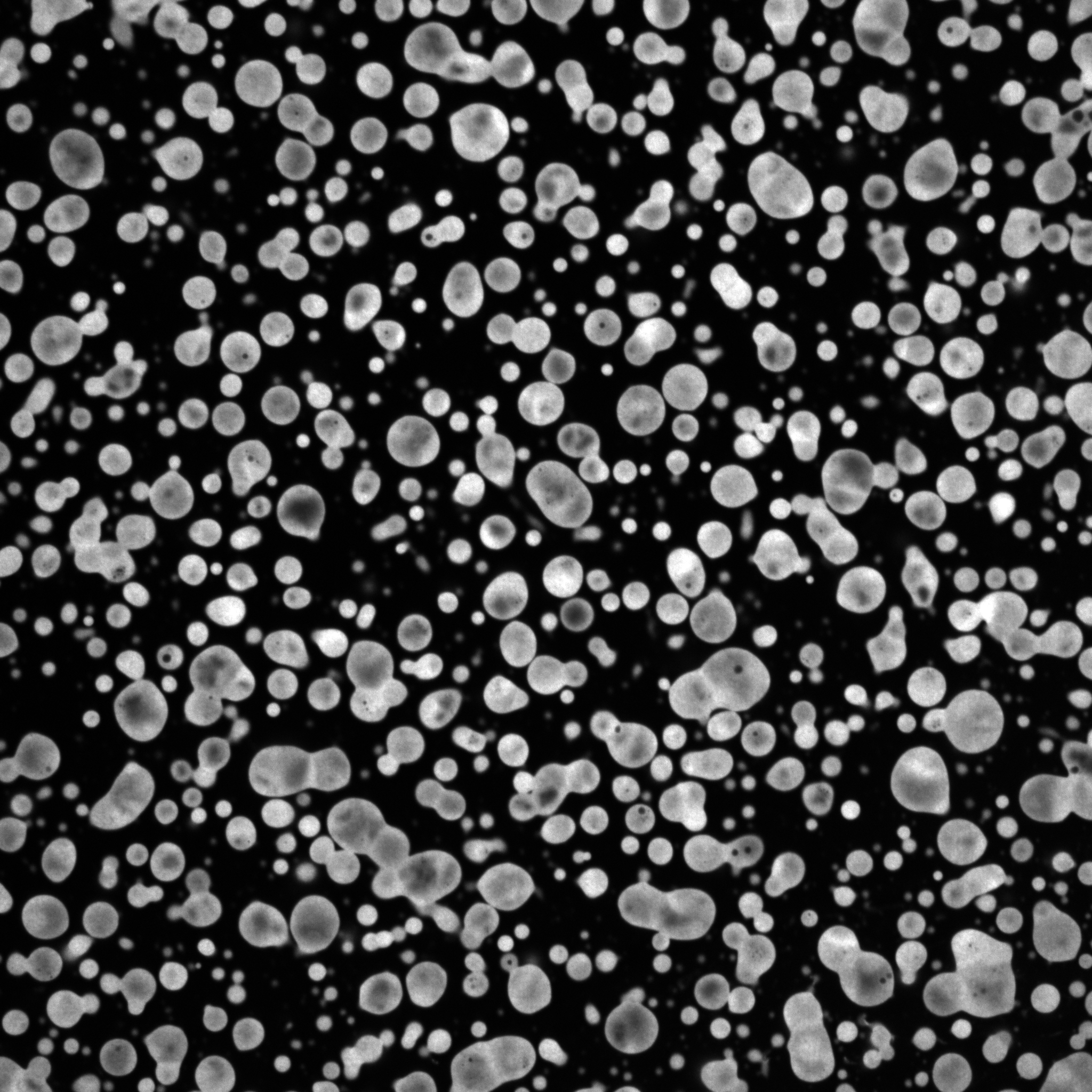

Supplement: Supplementary file 10 — Source data Fig. 5 [file 44319_2024_285_MOESM10_ESM.zip › Fig5/Fig5D/Without NAD mCh-PARP1 channel.tif]

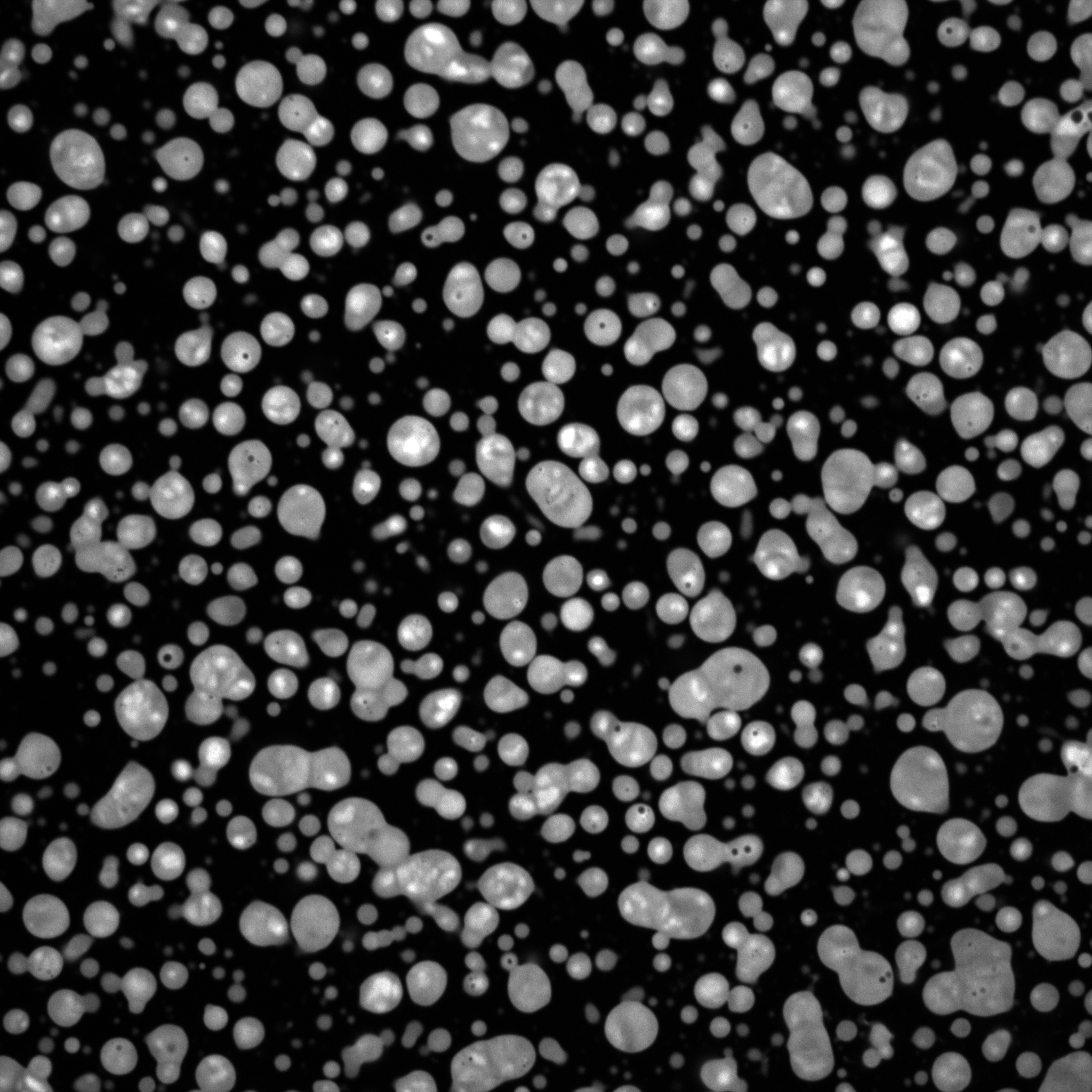

Supplement: Supplementary file 10 — Source data Fig. 5 [file 44319_2024_285_MOESM10_ESM.zip › Fig5/Fig5D/Without NAD Triplex DNA channel.tif]

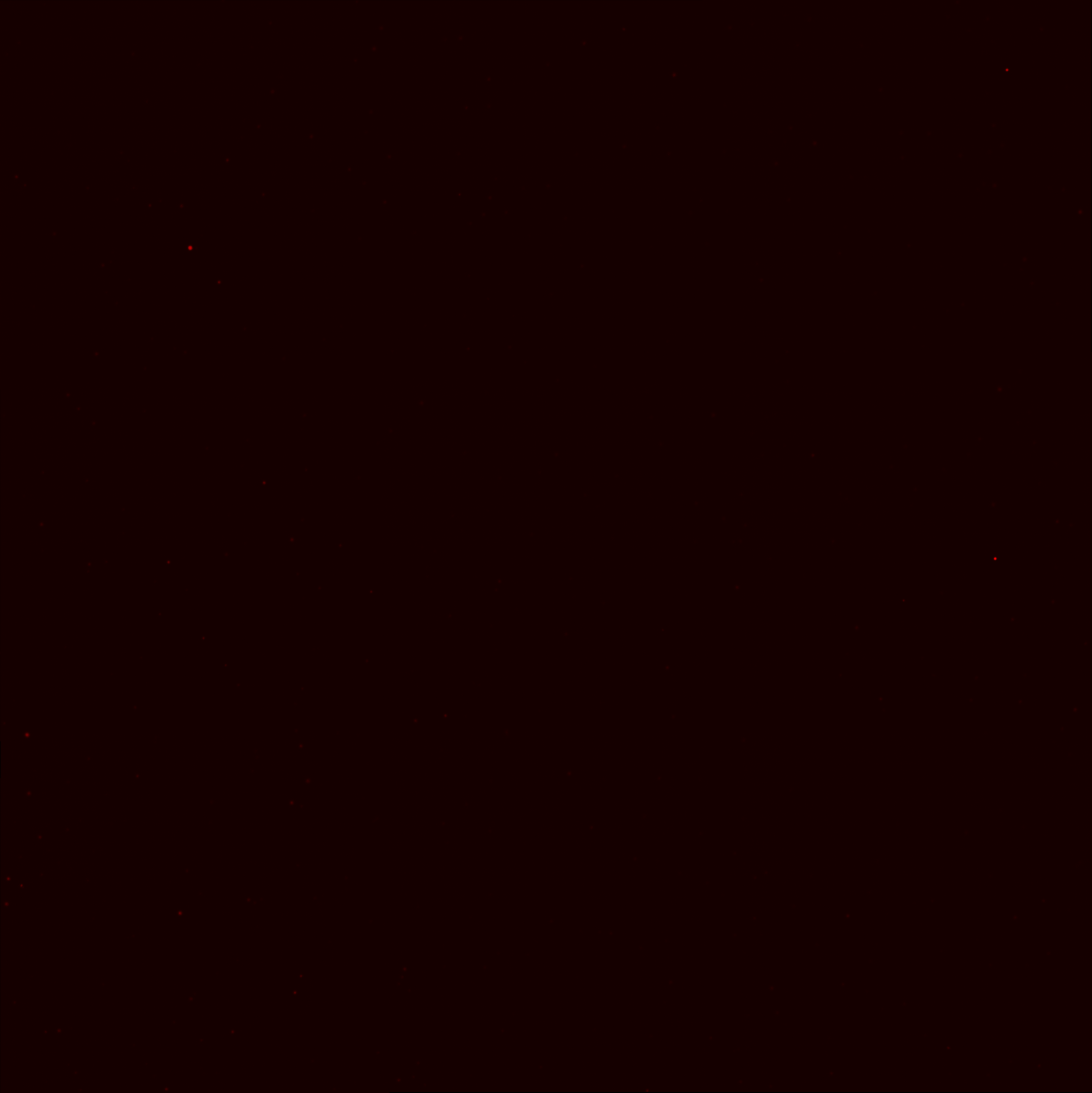

Supplement: Supplementary file 10 — Source data Fig. 5 [file 44319_2024_285_MOESM10_ESM.zip › Fig5/Fig5E/mChPARP1_NAD_Cy5TriplexDNA.tif]

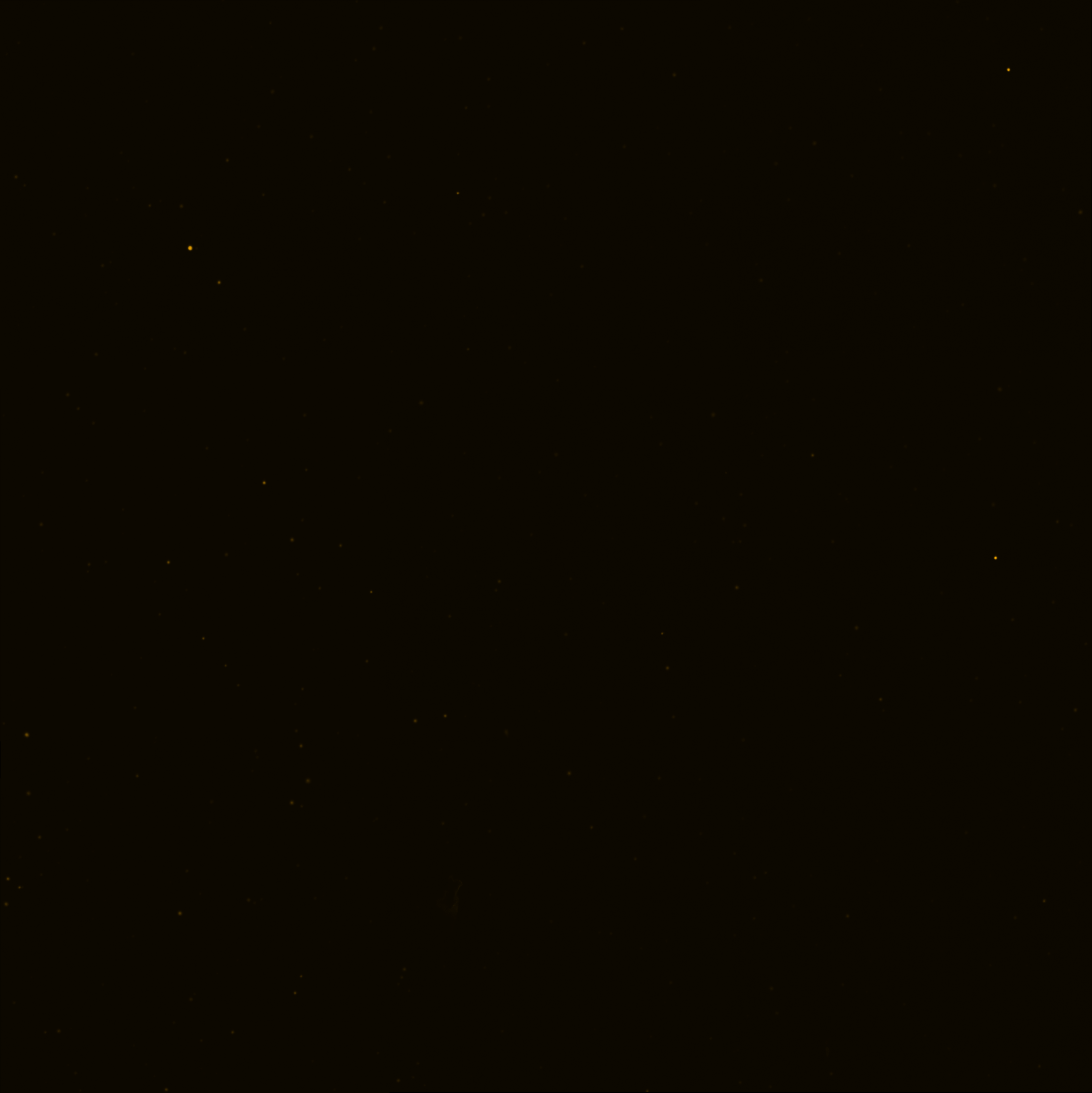

Supplement: Supplementary file 10 — Source data Fig. 5 [file 44319_2024_285_MOESM10_ESM.zip › Fig5/Fig5E/mChPARP1_NAD_mChPARP1.tif]

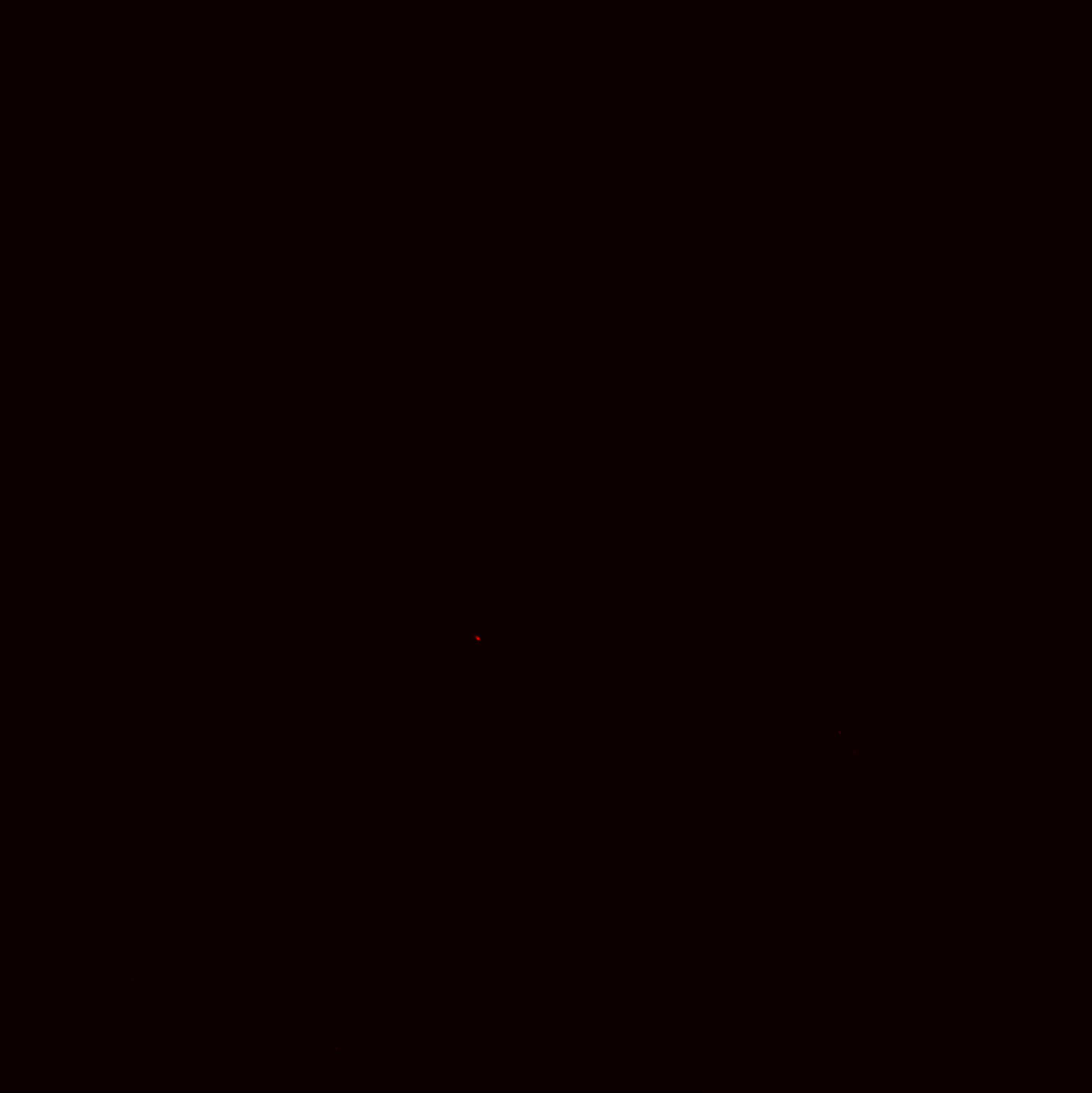

Supplement: Supplementary file 10 — Source data Fig. 5 [file 44319_2024_285_MOESM10_ESM.zip › Fig5/Fig5E/mChPARP1_noNAD_Cy5TriplexDNA.tif]

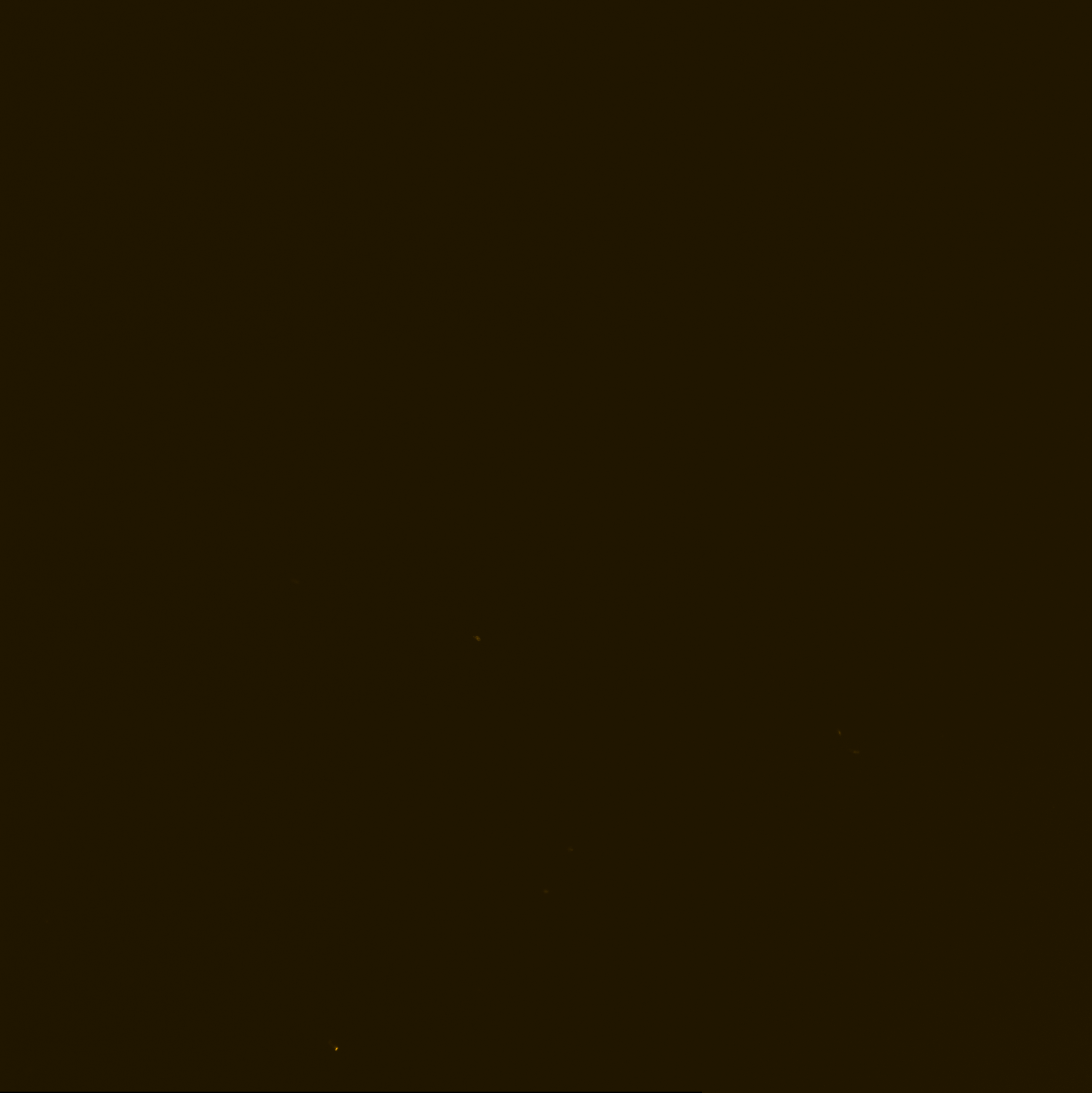

Supplement: Supplementary file 10 — Source data Fig. 5 [file 44319_2024_285_MOESM10_ESM.zip › Fig5/Fig5E/mChPARP1_noNAD_mChPARP1.tif]

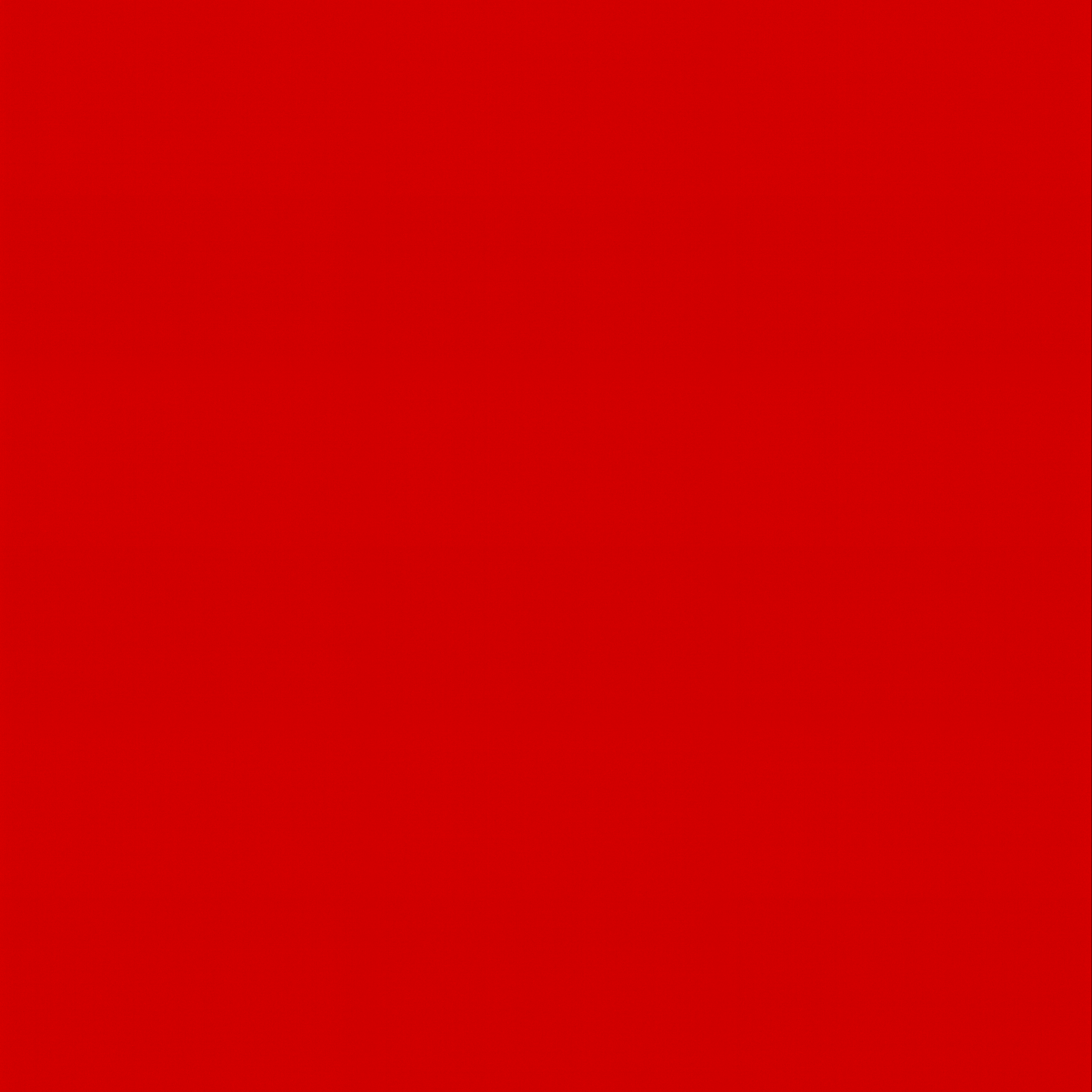

Supplement: Supplementary file 10 — Source data Fig. 5 [file 44319_2024_285_MOESM10_ESM.zip › Fig5/Fig5E/NomChPARP1_NAD_Cy5Triplex.tif]

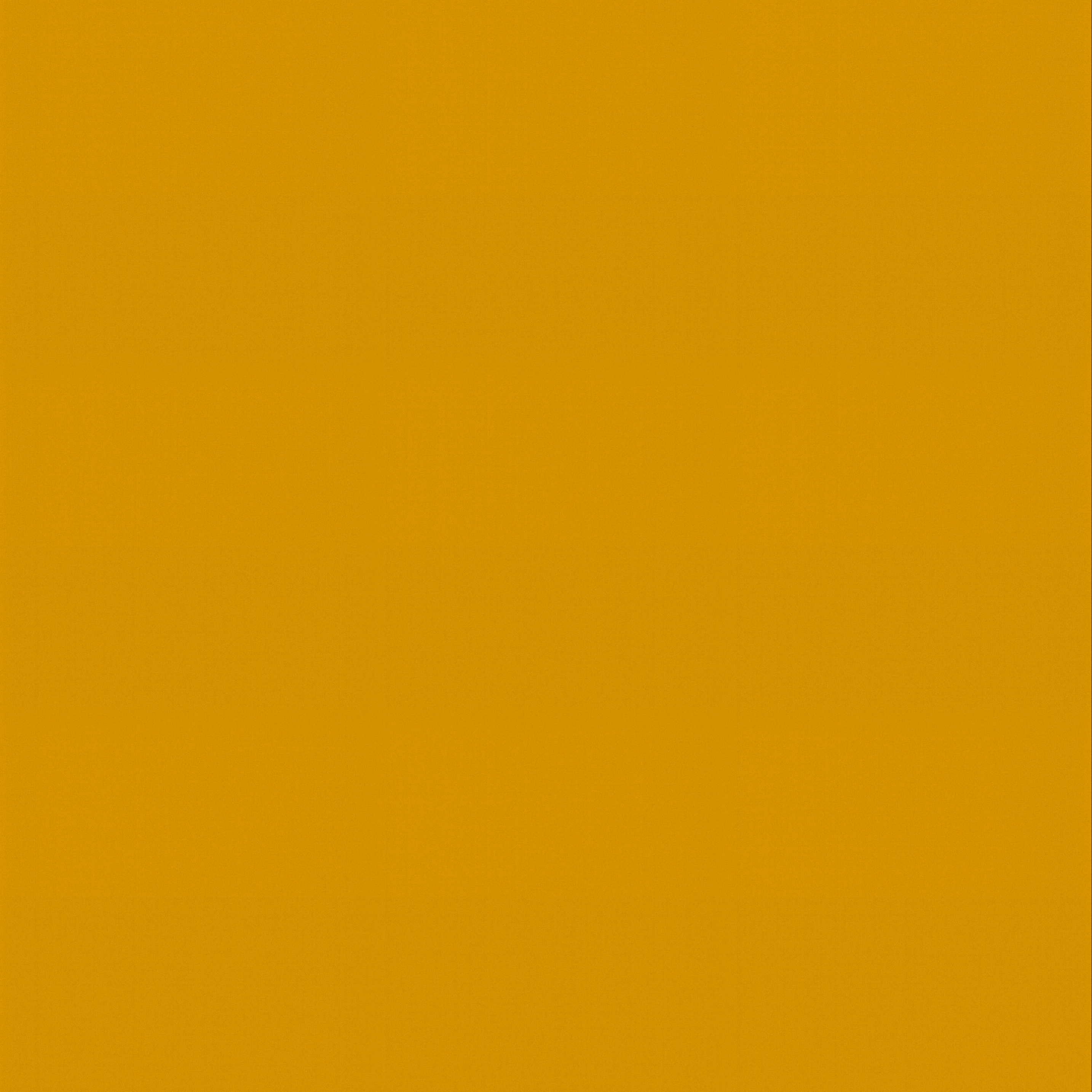

Supplement: Supplementary file 10 — Source data Fig. 5 [file 44319_2024_285_MOESM10_ESM.zip › Fig5/Fig5E/NomChPARP1_NAD_mChPARP1.tif]

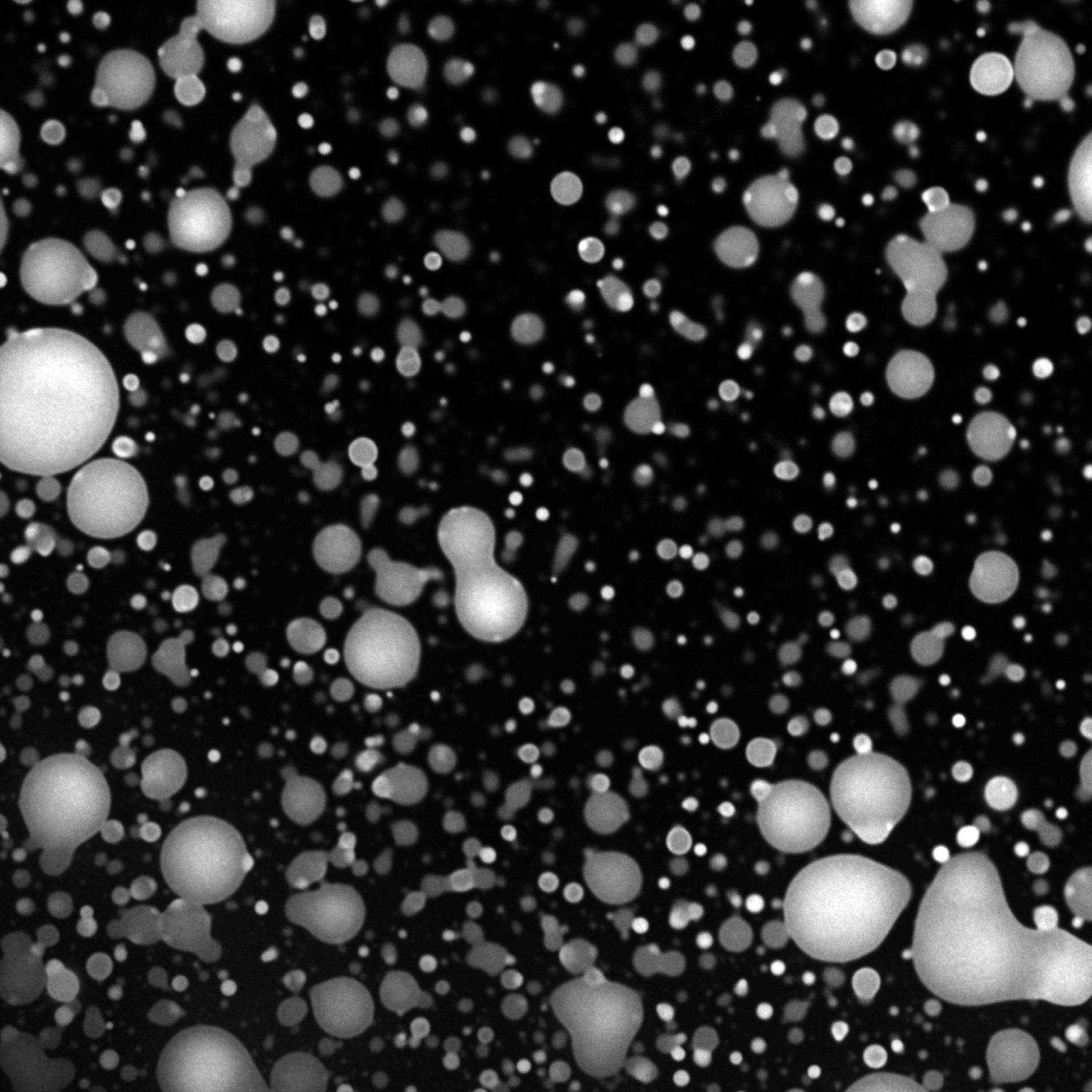

Supplement: Supplementary file 10 — Source data Fig. 5 [file 44319_2024_285_MOESM10_ESM.zip › Fig5/Fig5F/DNA Triplex 11min.tif]

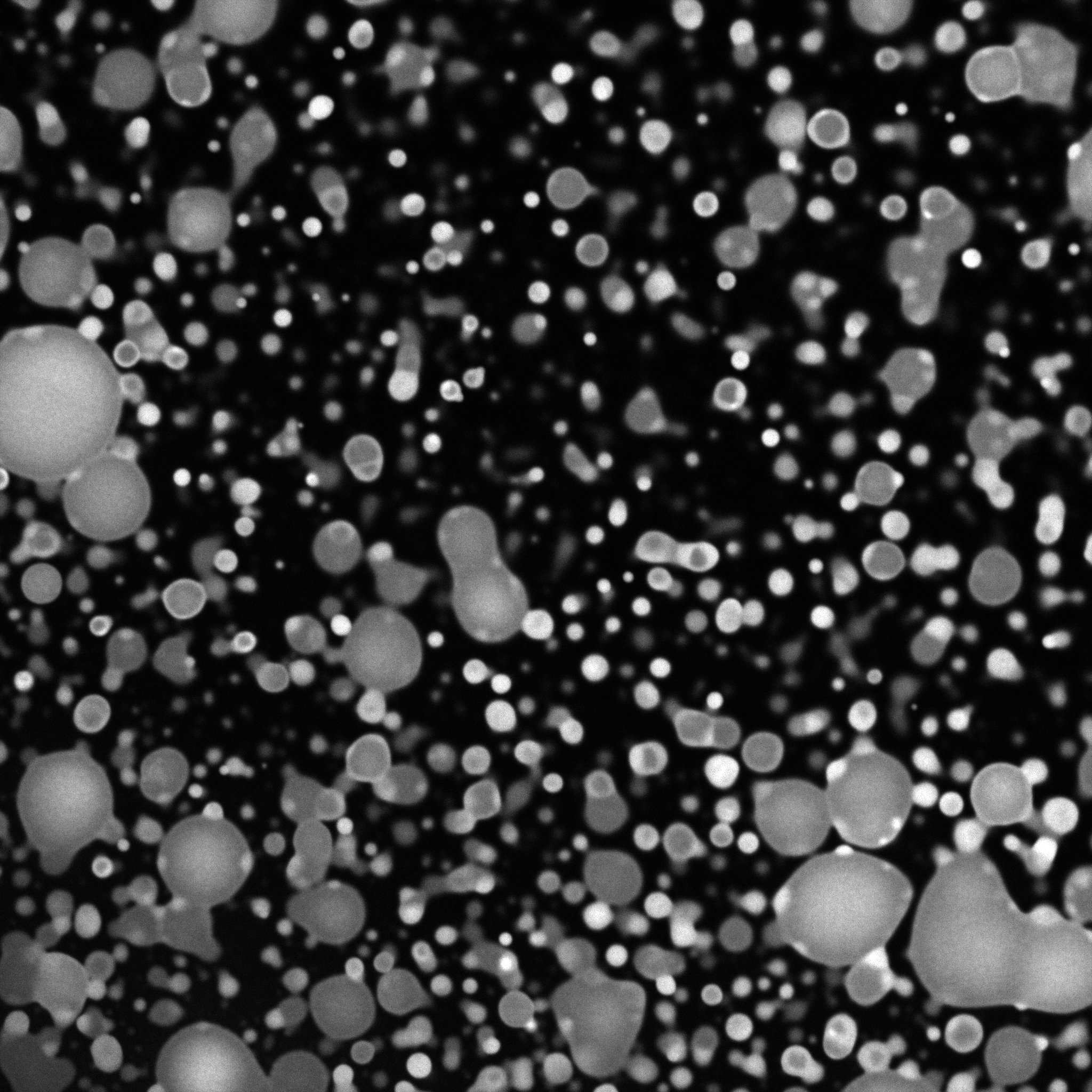

Supplement: Supplementary file 10 — Source data Fig. 5 [file 44319_2024_285_MOESM10_ESM.zip › Fig5/Fig5F/DNA Triplex 14min.tif]

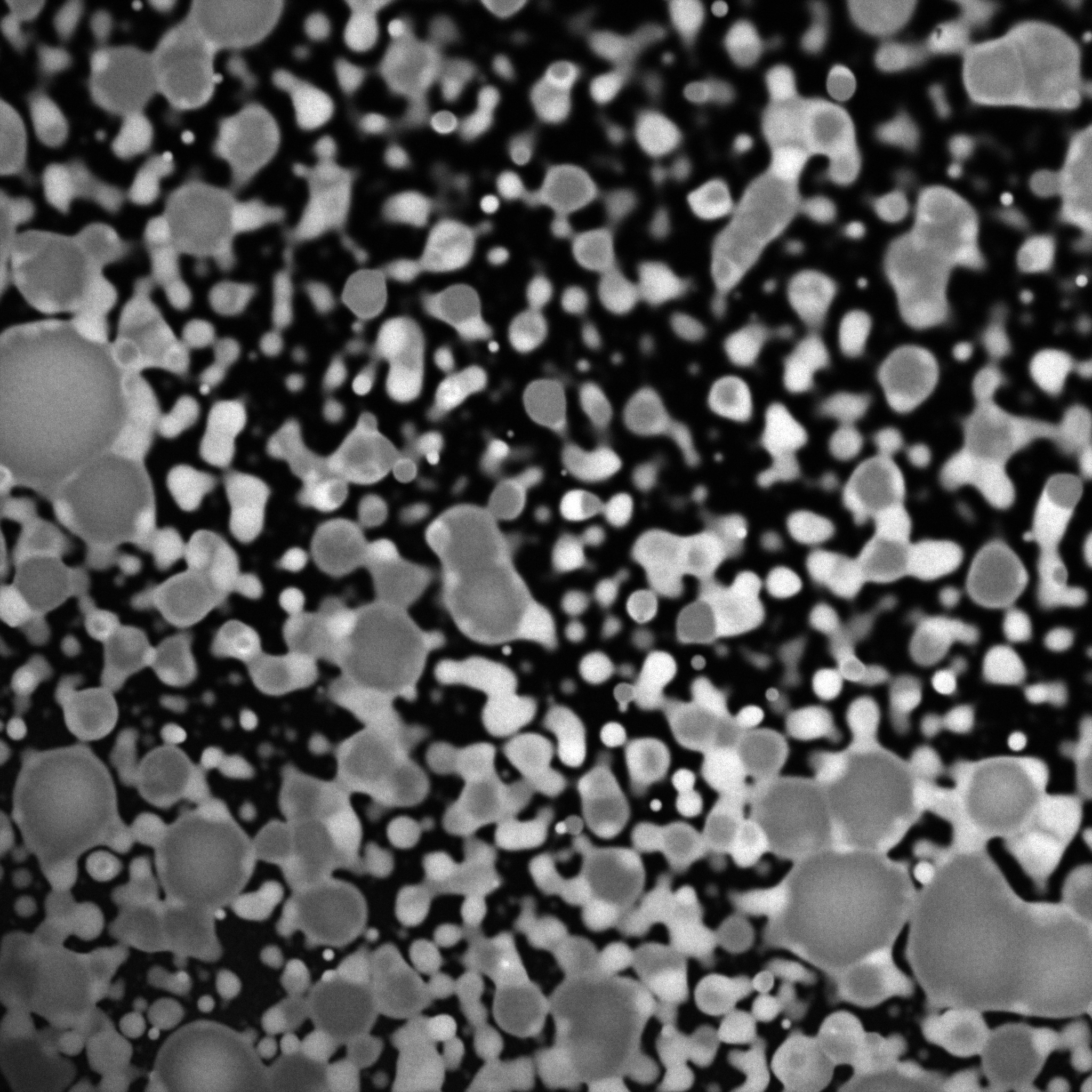

Supplement: Supplementary file 10 — Source data Fig. 5 [file 44319_2024_285_MOESM10_ESM.zip › Fig5/Fig5F/DNA Triplex 17min.tif]

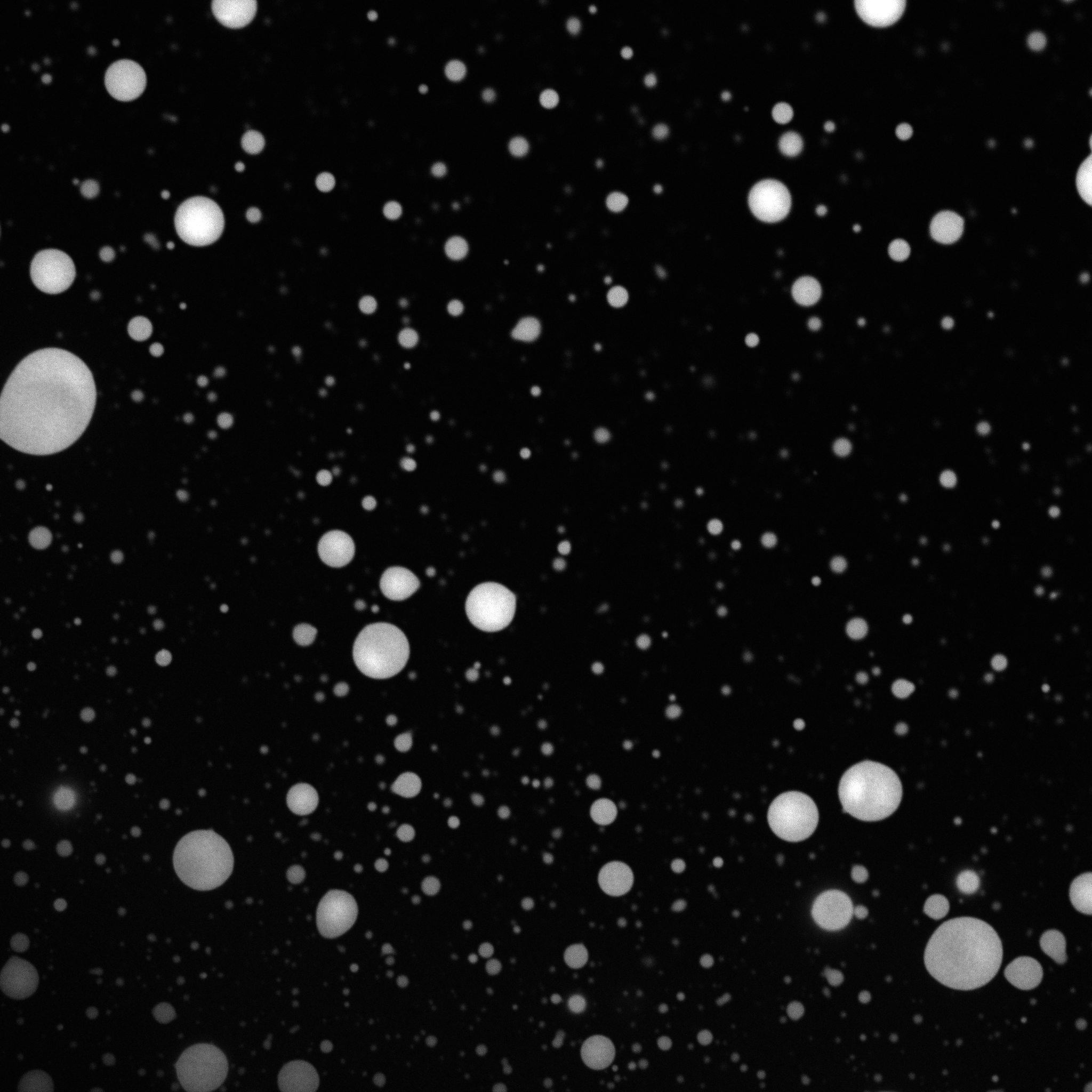

Supplement: Supplementary file 10 — Source data Fig. 5 [file 44319_2024_285_MOESM10_ESM.zip › Fig5/Fig5F/DNA Triplex 5min.tif]

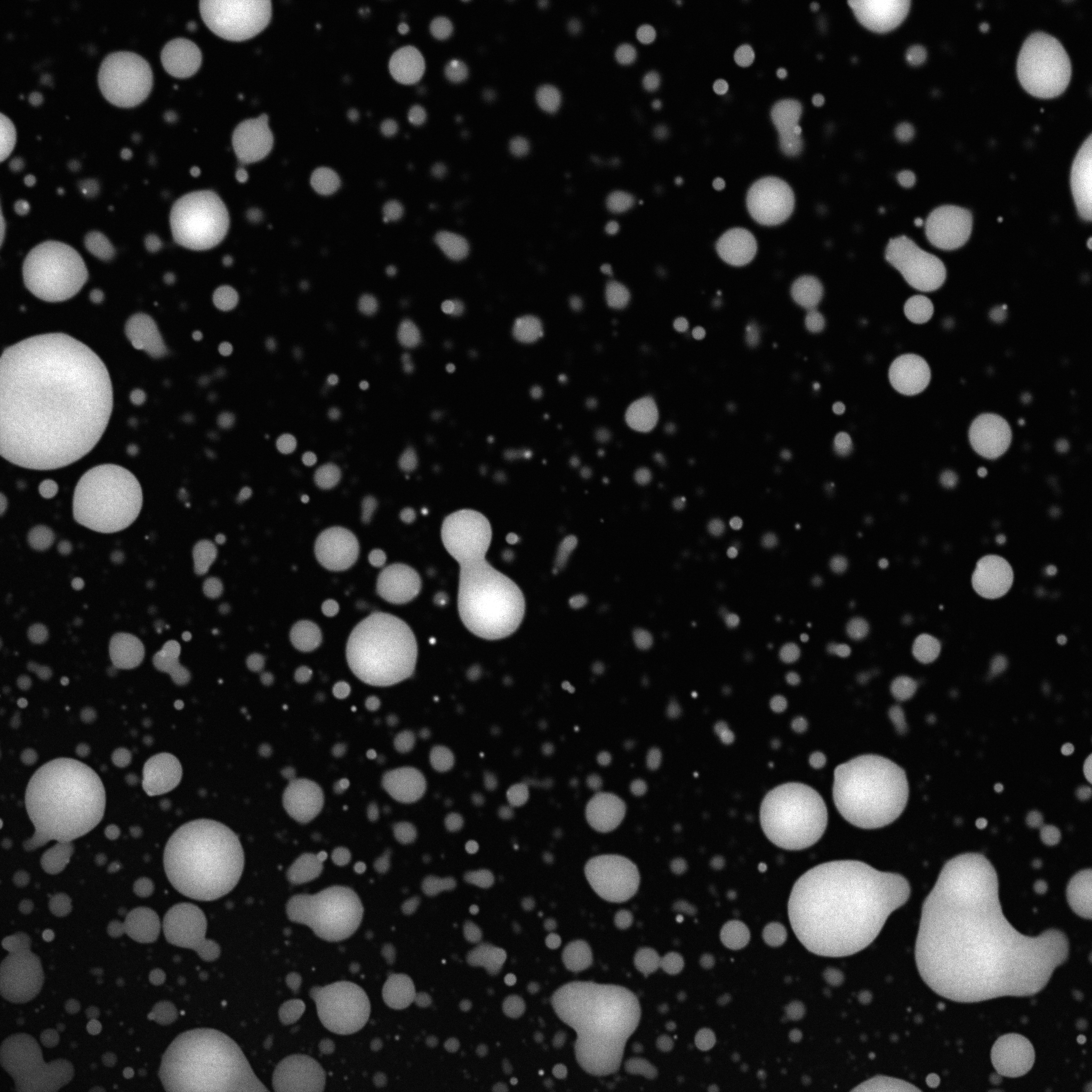

Supplement: Supplementary file 10 — Source data Fig. 5 [file 44319_2024_285_MOESM10_ESM.zip › Fig5/Fig5F/DNA Triplex 8min.tif]

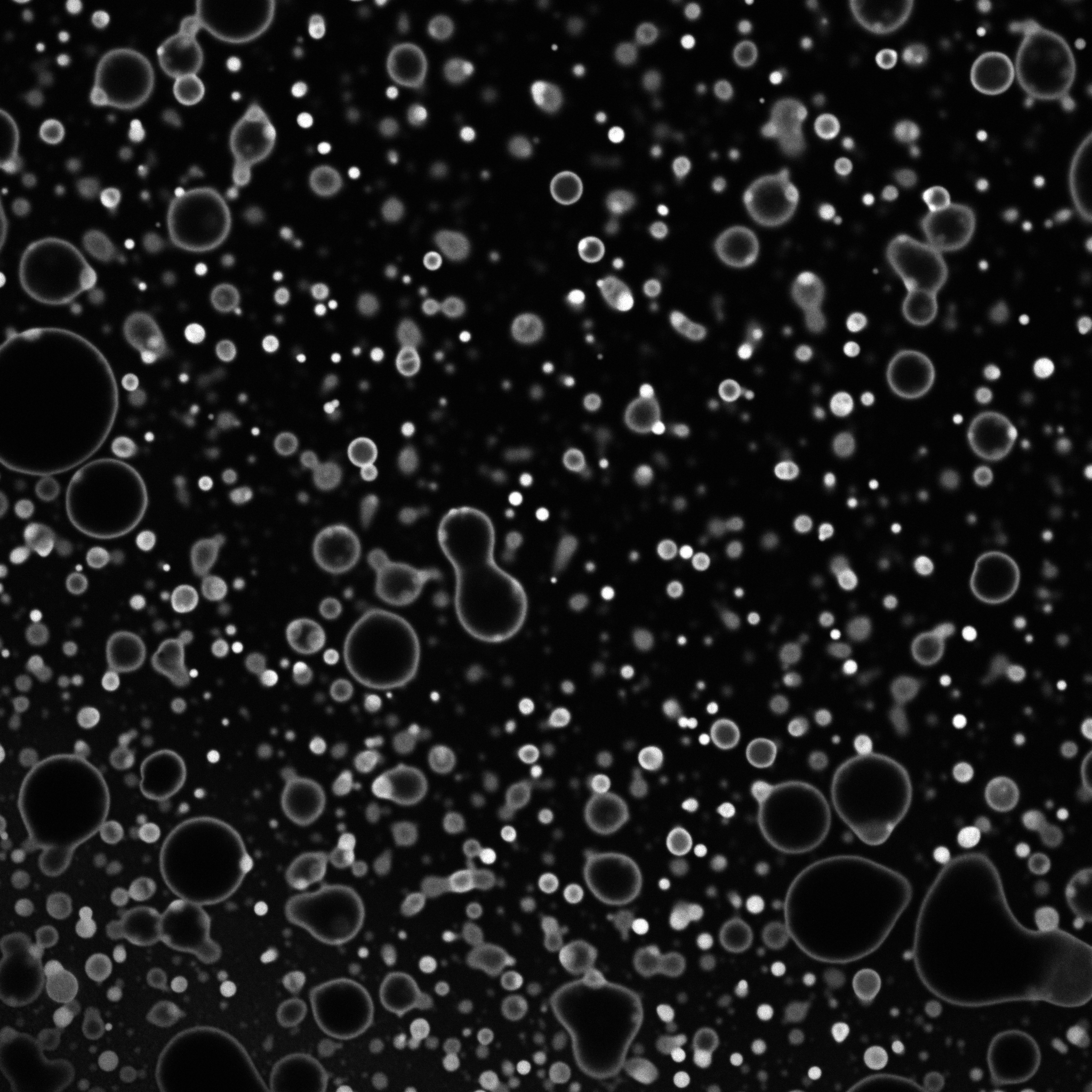

Supplement: Supplementary file 10 — Source data Fig. 5 [file 44319_2024_285_MOESM10_ESM.zip › Fig5/Fig5F/Lig3 11min.tif]

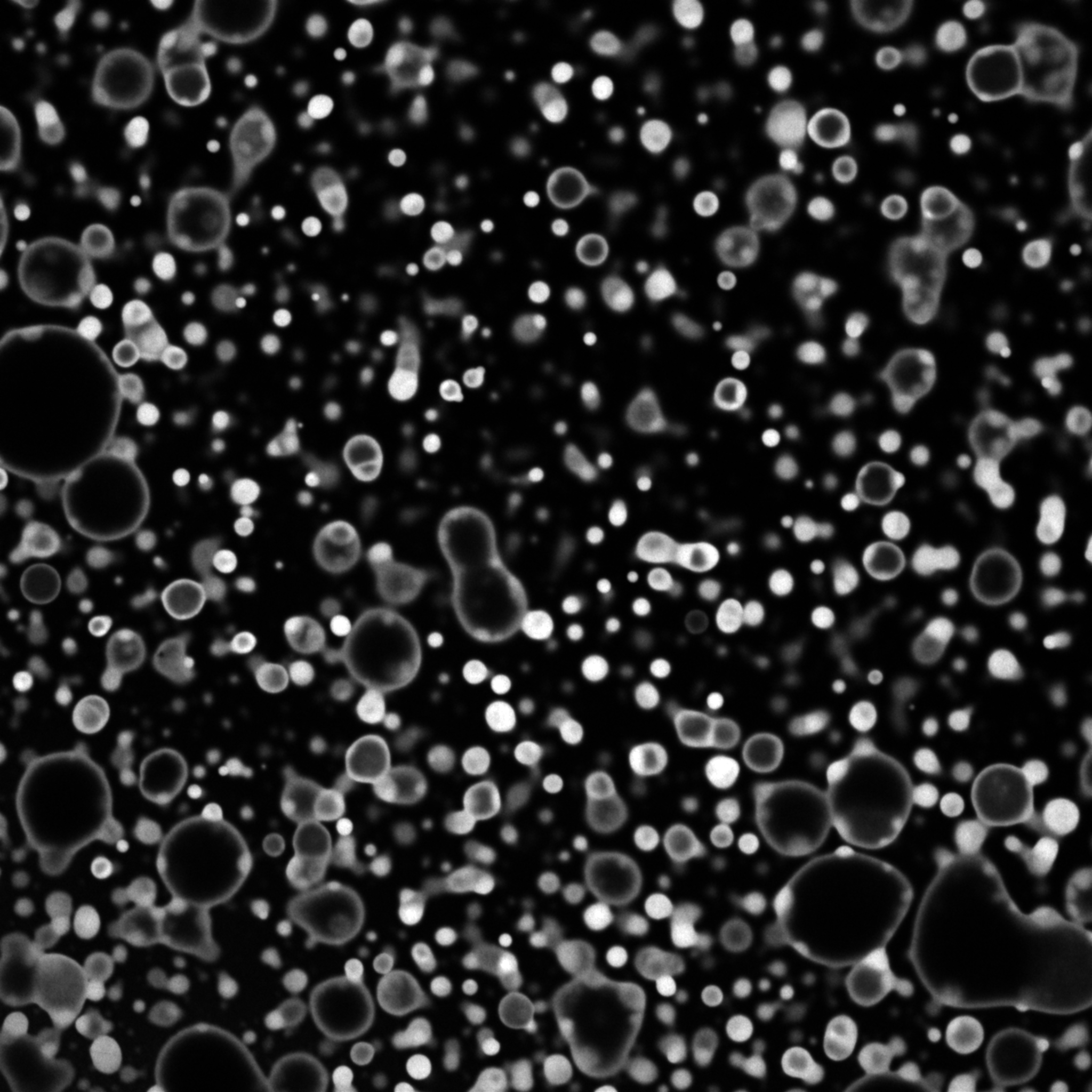

Supplement: Supplementary file 10 — Source data Fig. 5 [file 44319_2024_285_MOESM10_ESM.zip › Fig5/Fig5F/Lig3 14min.tif]

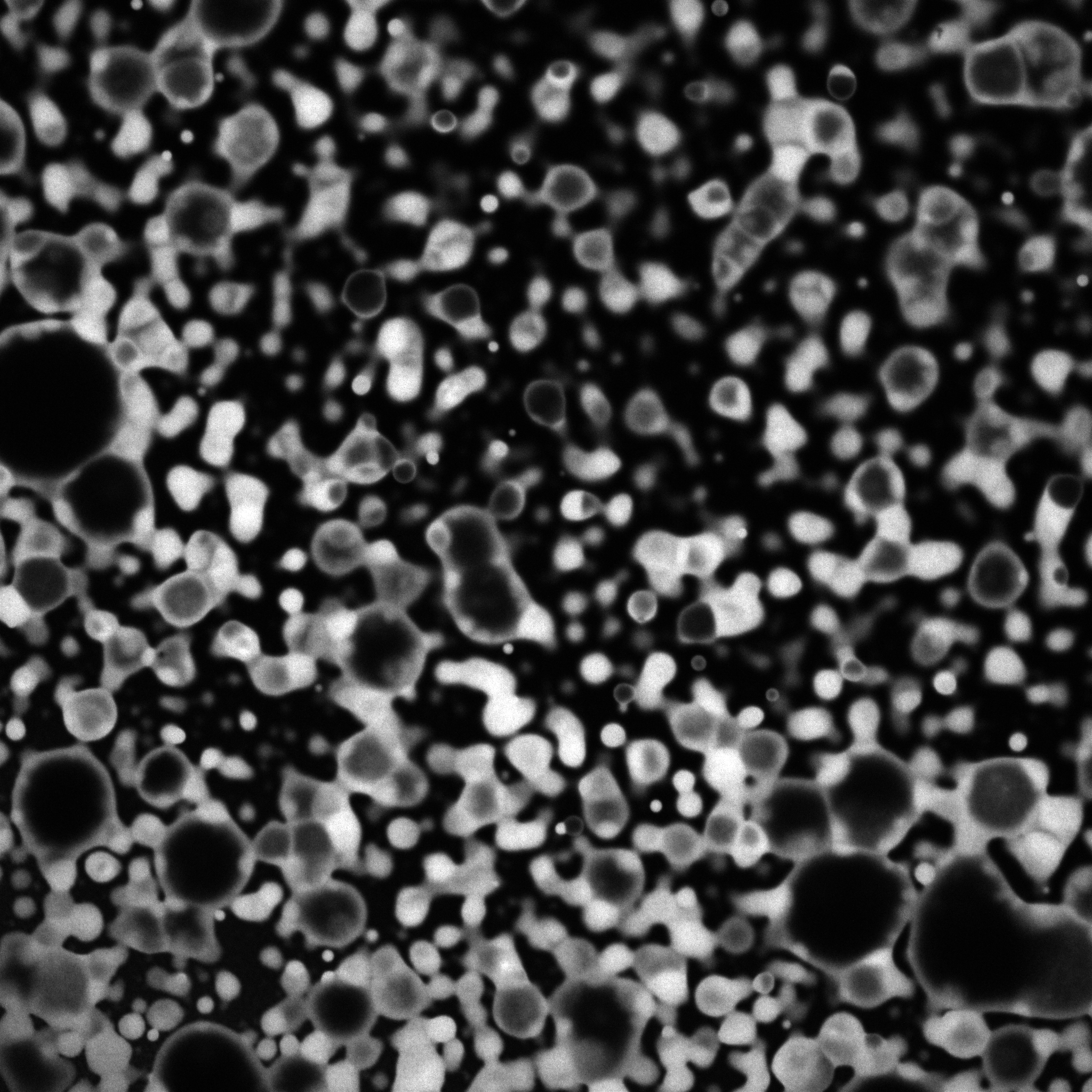

Supplement: Supplementary file 10 — Source data Fig. 5 [file 44319_2024_285_MOESM10_ESM.zip › Fig5/Fig5F/Lig3 17min.tif]

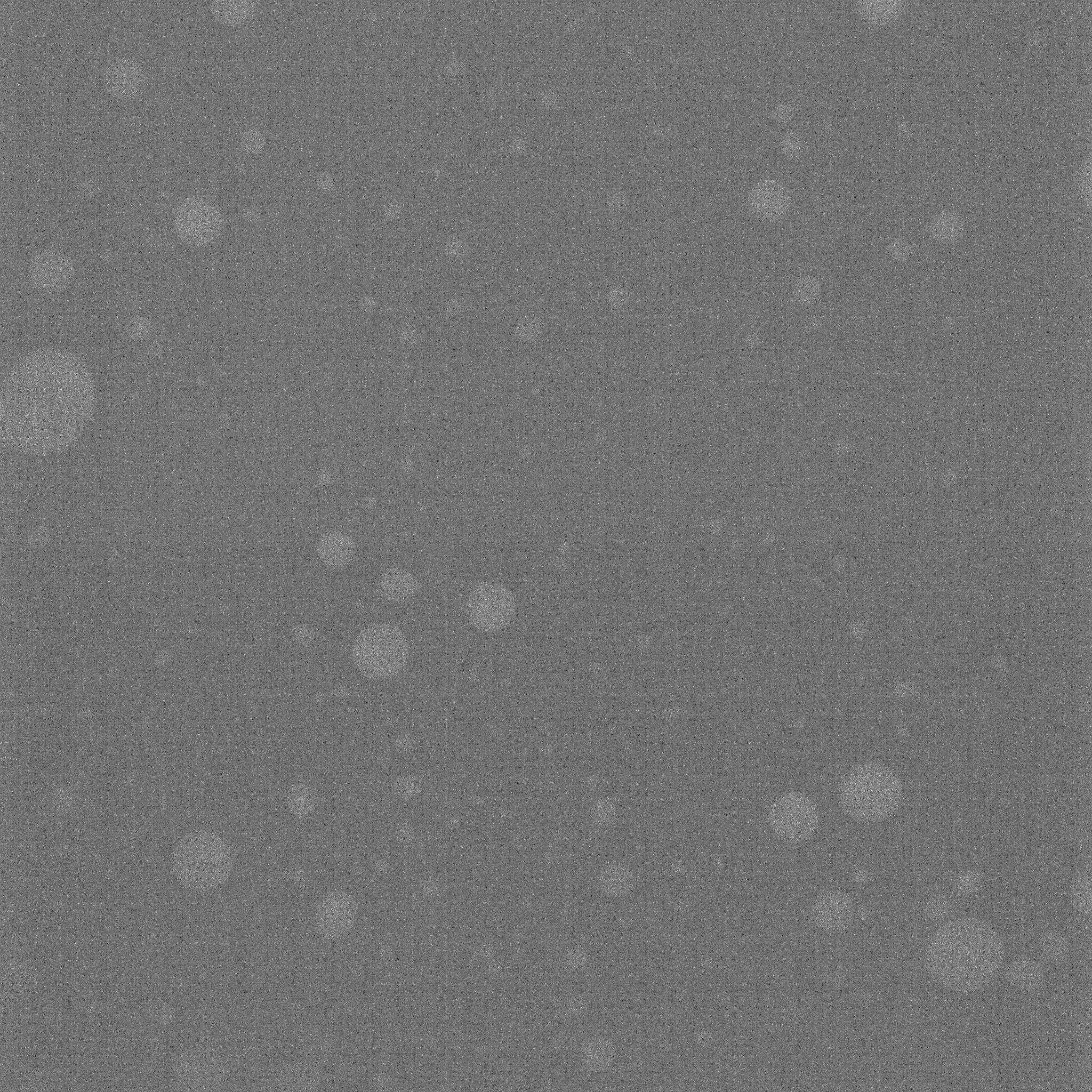

Supplement: Supplementary file 10 — Source data Fig. 5 [file 44319_2024_285_MOESM10_ESM.zip › Fig5/Fig5F/Lig3 5min.tif]

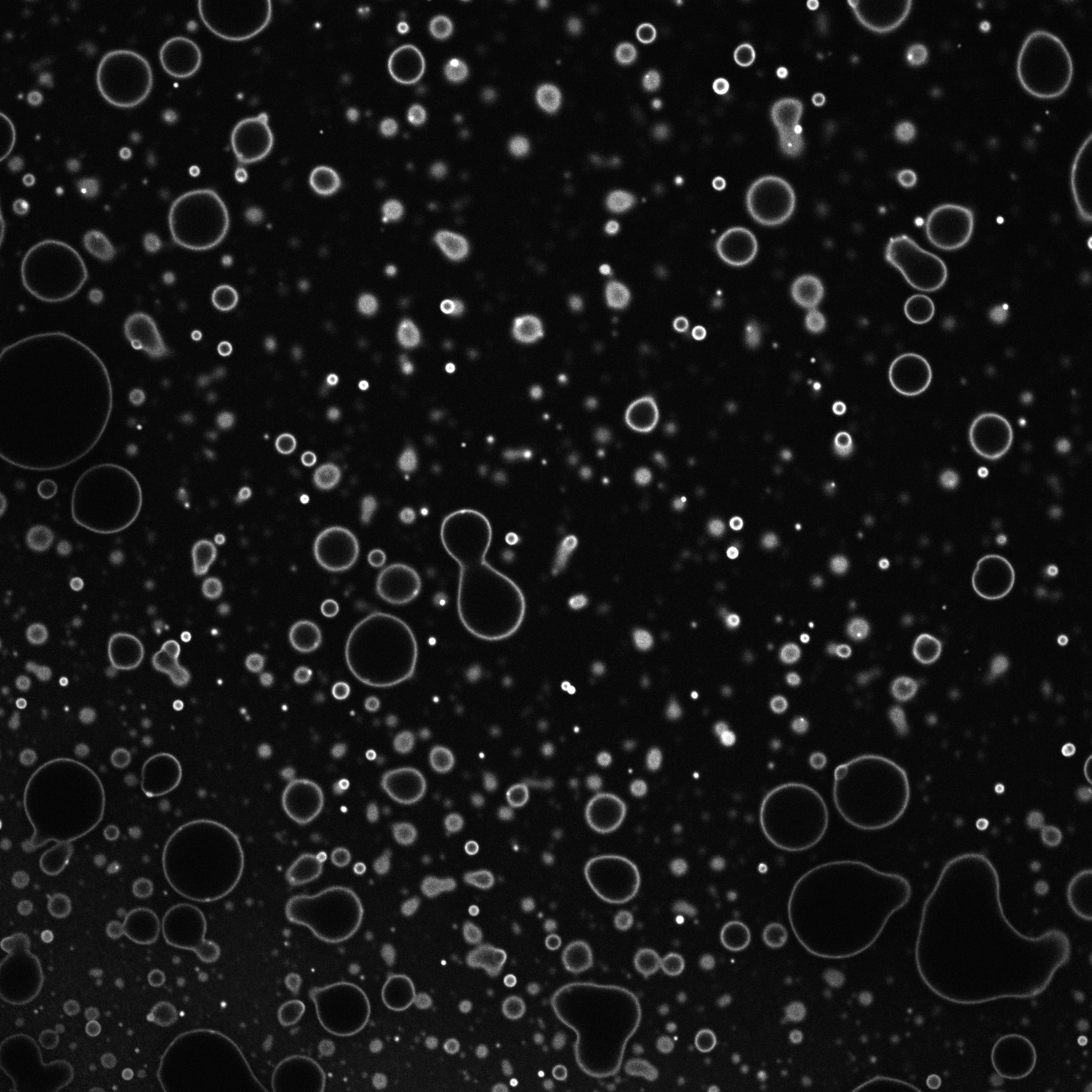

Supplement: Supplementary file 10 — Source data Fig. 5 [file 44319_2024_285_MOESM10_ESM.zip › Fig5/Fig5F/Lig3 8min.tif]

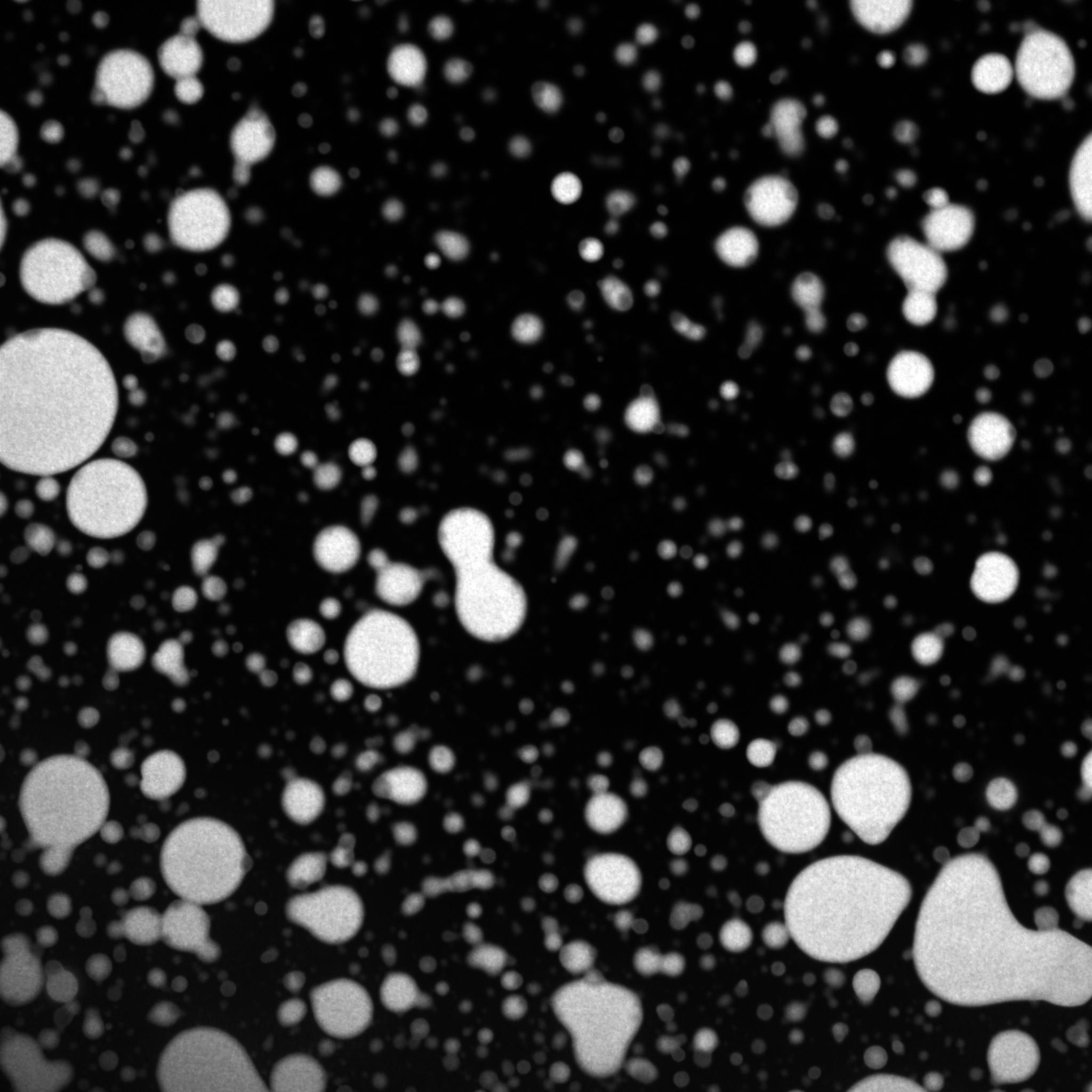

Supplement: Supplementary file 10 — Source data Fig. 5 [file 44319_2024_285_MOESM10_ESM.zip › Fig5/Fig5F/mCh-PARP1 11min.tif]

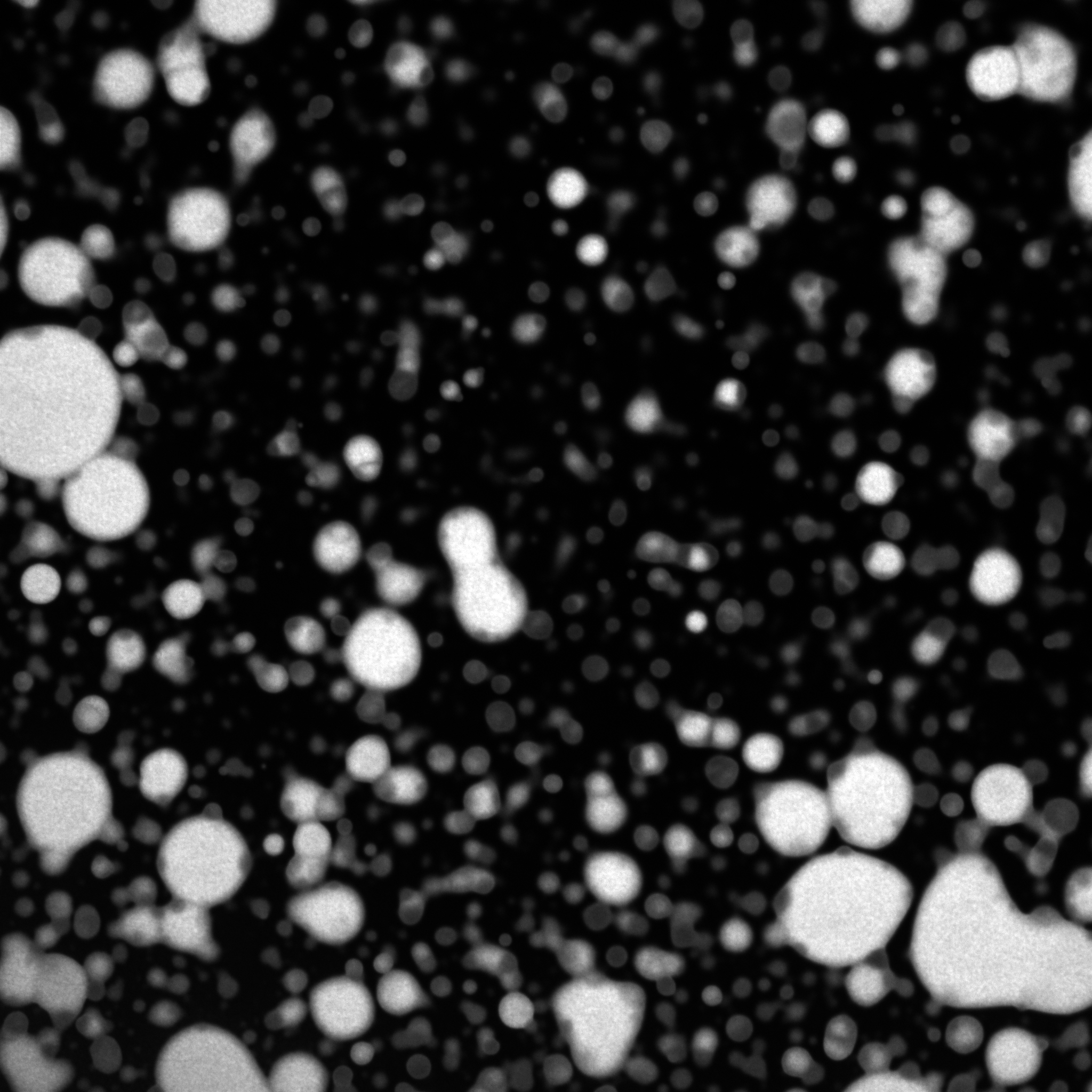

Supplement: Supplementary file 10 — Source data Fig. 5 [file 44319_2024_285_MOESM10_ESM.zip › Fig5/Fig5F/mCh-PARP1 14min.tif]

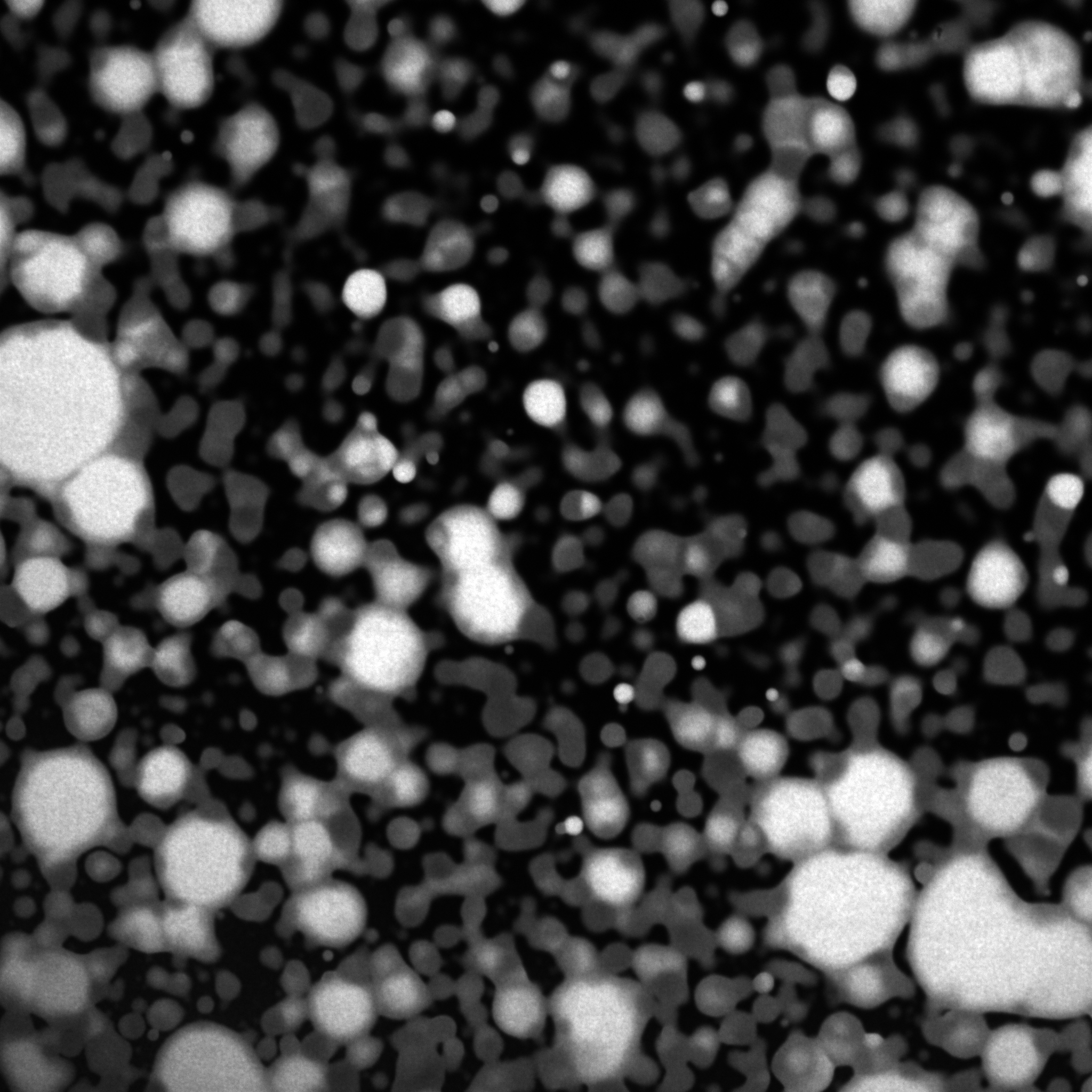

Supplement: Supplementary file 10 — Source data Fig. 5 [file 44319_2024_285_MOESM10_ESM.zip › Fig5/Fig5F/mCh-PARP1 17min.tif]

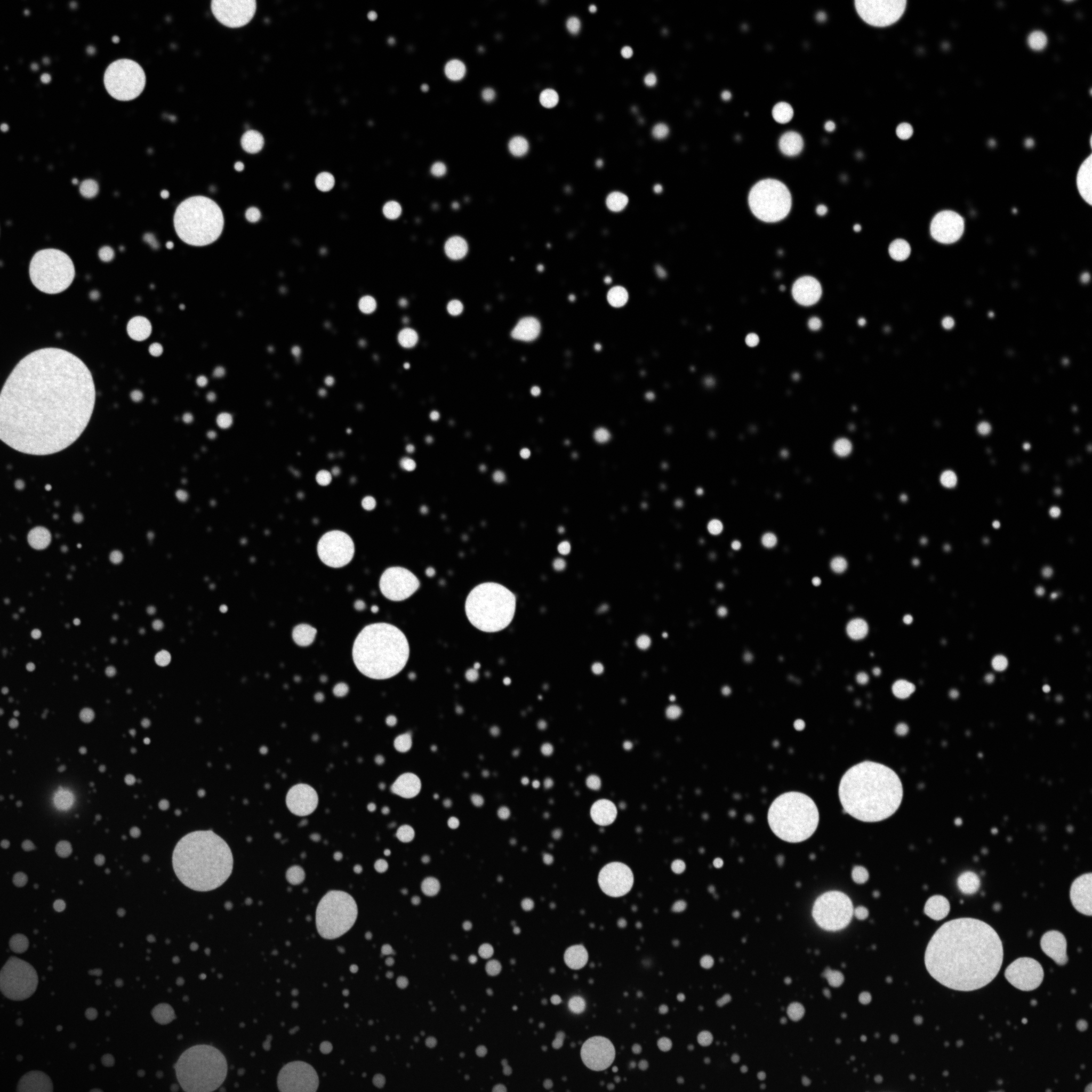

Supplement: Supplementary file 10 — Source data Fig. 5 [file 44319_2024_285_MOESM10_ESM.zip › Fig5/Fig5F/mCh-PARP1 5min.tif]

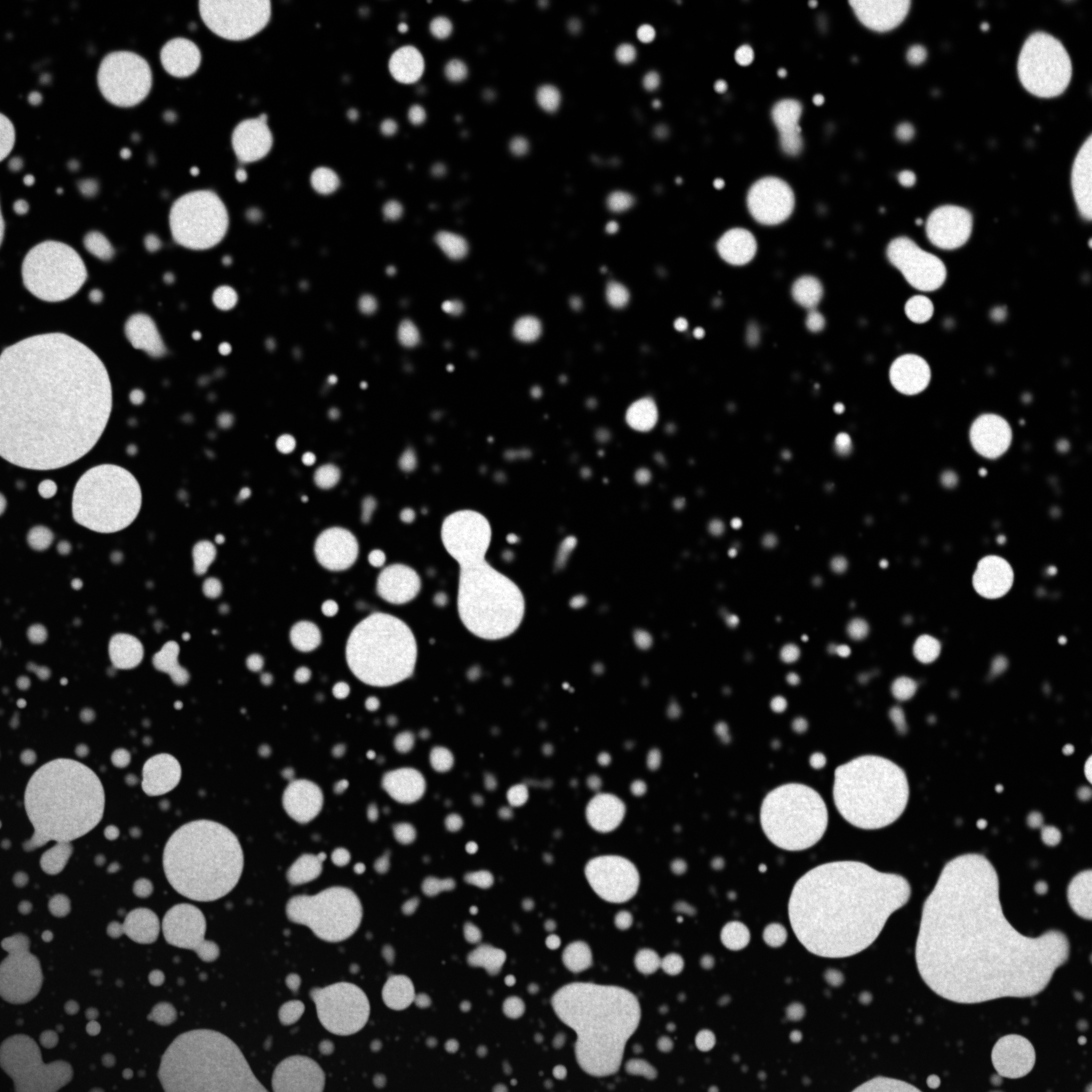

Supplement: Supplementary file 10 — Source data Fig. 5 [file 44319_2024_285_MOESM10_ESM.zip › Fig5/Fig5F/mCh-PARP1 8min.tif]
