# Supplementary material for: PARP1 condensates differentially partition DNA repair proteins and enhance DNA ligation
Source: EMBO Rep. 2024 Nov 4;25(12):5635–66. doi: 10.1038/s44319-024-00285-5 (PMC11624282; doi:10.1038/s44319-024-00285-5)
Supplement: Supplementary file 11 — Expanded View Figures [file 44319_2024_285_MOESM11_ESM.pdf]

Expanded View Figures

Figure EV1. PARP1 forms condensates in a DNA-dependent manner.

(**A**) IUPRED3 disordered region plots for PARP1 (https://iupred.elte.hu/plot). (**B**) Image of Coomassie Blue stained SDS-PAGE of purified recombinant mCherry-PARP1. (**C**) Fluorescence micrographs of 4 µM mCherry-PARP1 with or without the indicated concentration of dumbbell DNA. (**D**) Fluorescence micrographs of 4 µM mCherry-PARP1 with the indicated lengths and concentrations of double-stranded DNA (dsDNA). (**E**) Fluorescence micrographs of 4 µM mCherry-PARP1 with the indicated concentrations of pUC19 plasmid DNA with or without a single nick. (**F**) Fluorescence micrographs of 4 µM mCherry-PARP1 and 4 µM triplex DNA condensates before and after addition of 100 mM NaCl to dissolve pre-existing mCherry-PARP1 condensates. (**G**) Fluorescence micrographs of 4 µM mCherry-PARP1 with or without 4 µM triplex DNA in the presence or absence of 10% 1,6-hexanediol. (**H**) Phase diagram of condensates formed by recombinant mCherry-PARP1 at indicated concentrations with or without single-stranded DNA. (**I**) Representative fluorescence micrographs of mCherry-PARP1 condensates diagramed in (**H**). All figures represent findings from at least three biological replicates.

© The Author(s)

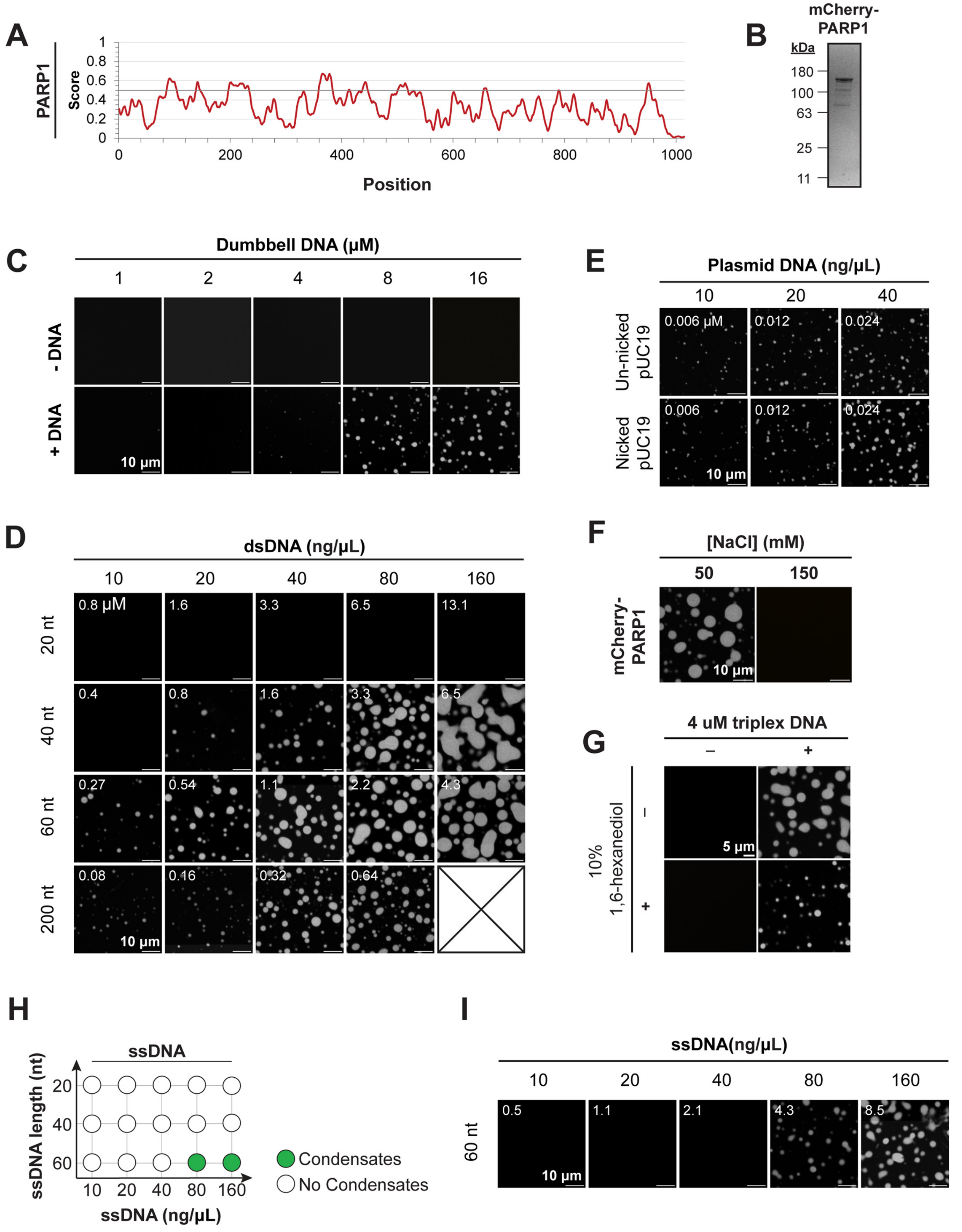
A
PARP1
Score
1
0.8
0.6
0.4
0.2
0
0
200
400
600
800
1000
Position
B
mCherry-PARP1
kDa
180
100
63
25
11
C
Dumbbell DNA (μM)
1 2 4 8 16
- DNA
+ DNA
10 μm
D
dsDNA (ng/μL)
10 20 40 80 160
20 nt
0.8 μM 1.6 3.3 6.5 13.1
40 nt
0.4 0.8 1.6 3.3 6.5
60 nt
0.27 0.54 1.1 2.2 4.3
200 nt
0.08 0.16 0.32 0.64
10 μm
E
Plasmid DNA (ng/μL)
10 20 40
Un-nicked pUC19
0.006 μM 0.012 0.024
Nicked pUC19
0.006 0.012 0.024
10 μm
F
[NaCl] (mM)
50 150
mCherry-PARP1
10 μm
G
4 uM triplex DNA
– +
10% 1,6-hexanediol
–
+
5 μm
H
ssDNA
ssDNA length (nt)
20
40
60
10 20 40 80 160
ssDNA (ng/μL)
Condensates
No Condensates
I
ssDNA(ng/μL)
10 20 40 80 160
60 nt
0.5 1.1 2.1 4.3 8.5
10 μm

 © The Author(s)

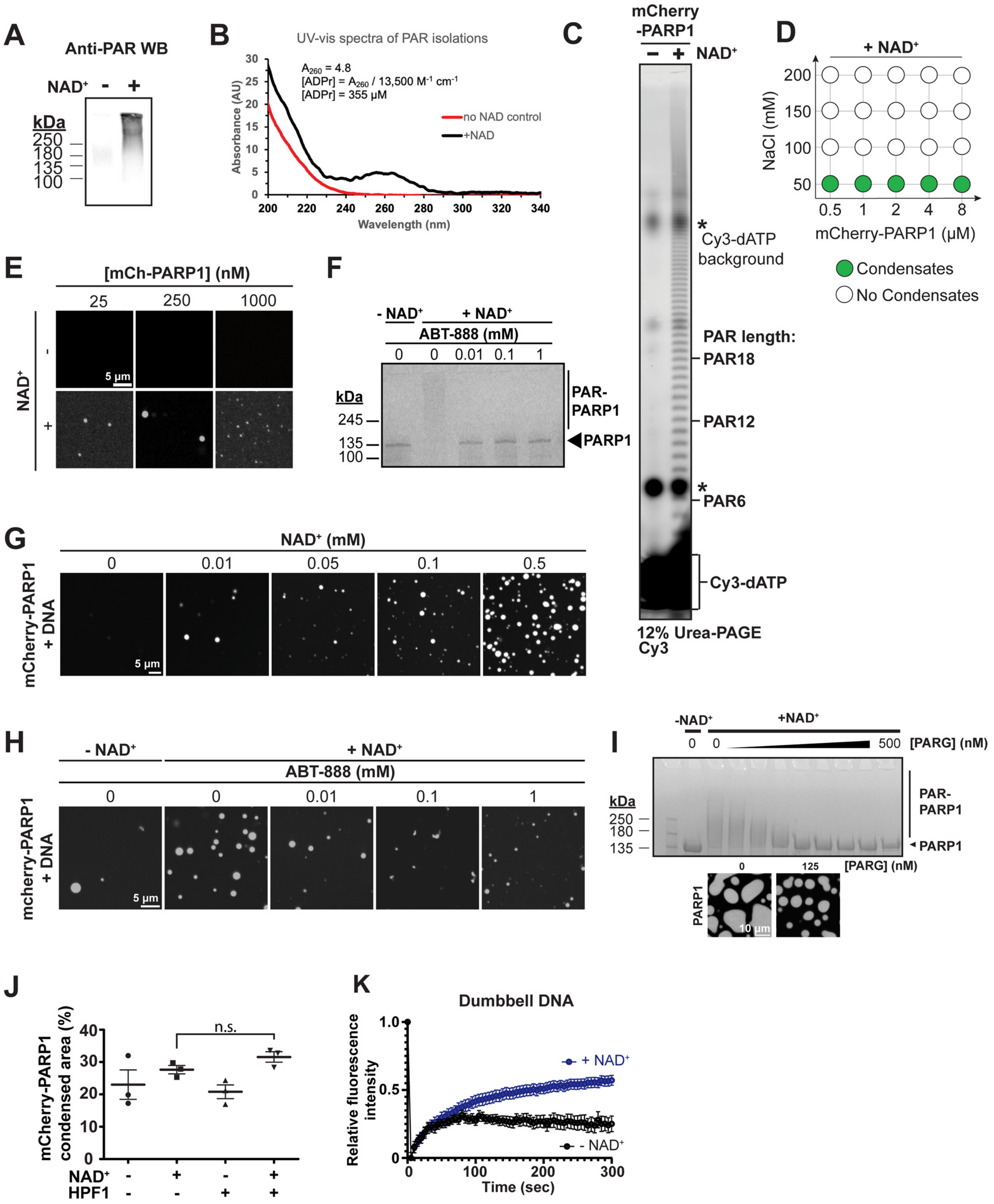
A
Anti-PAR WB
NAD+ - +
kDa
250
180
135
100
B
UV-vis spectra of PAR isolations
A260 = 4.8
[ADPr] = A260 / 13,500 M-1 cm-1
[ADPr] = 355 μM
no NAD control
+NAD
Absorbance (AU)
Wavelength (nm)
C
mCherry -PARP1
NAD+
Cy3-dATP background
PAR length:
PAR18
PAR12
PAR6
Cy3-dATP
12% Urea-PAGE Cy3
D
+ NAD+
NaCl (mM)
mCherry-PARP1 (μM)
Condensates
No Condensates
E
[mCh-PARP1] (nM)
25 250 1000
NAD+
5 μm
F
- NAD+ + NAD+
ABT-888 (mM)
0 0 0.01 0.1 1
kDa
245
135
100
PAR-PARP1
PARP1
G
NAD+ (mM)
0 0.01 0.05 0.1 0.5
mCherry-PARP1 + DNA
5 μm
H
- NAD+ + NAD+
ABT-888 (mM)
0 0 0.01 0.1 1
mcherry-PARP1 + DNA
5 μm
I
-NAD+ +NAD+
0 0 500 [PARG] (nM)
kDa
250
180
135
PAR-PARP1
PARP1
0 125 [PARG] (nM)
PARP1
10 μm
J
mCherry-PARP1 condensed area (%)
n.s.
NAD+ - + - +
HPF1 - - + +
K
Dumbbell DNA
Relative fluorescence intensity
+ NAD+
- NAD+
Time (sec)

Figure EV2. AutoPARylation enhances the formation and internal dynamics of PARP1 condensates.

(**A**) Anti-PAR Western blot depicting autoPARylation of PARP1 from reactions of 1 μM untagged PARP1 incubated with 0.3 μM dumbbell DNA for 15 min with and without 0.5 mM NAD^+. (**B**) UV-Vis spectra of mCherry-PARP1 with 0.3 μM dumbbell DNA in PARylation Buffer for 15 min with and without 0.5 mM NAD^+. ADP-ribose concentration is estimated to be around 355 μM by the equation depicted. (**C**) Urea-PAGE of PAR extracted from mCherry-PARP1 in PARylation reactions with and without 0.5 mM NAD^+, labeled with Cy3-dATP. (**D**) Phase diagram of mCherry-PARP1 condensates formed at the indicated protein and salt concentrations in an autoPARylation reaction with 0.5 mM NAD^+. (**E**) Fluorescence micrograph of mCherry-PARP1 at indicated concentrations with 50 nM triplex DNA and with or without 0.5 mM NAD^+. (**F**) Gel-based autoPARylation assay performed with 1 μM mCherry-PARP1 with 0.3 μM triplex DNA, with or without 0.5 mM NAD^+, and increasing concentrations of ABT-888 (0, 0.01, 0.1, and 1 mM). (**G**) Fluorescence micrographs of 1 μM mCherry-PARP1 with 0.3 μM triplex DNA at indicated concentrations of NAD^+. (**H**) Fluorescence micrographs of 1 μM mCherry-PARP1 with 0.3 μM triplex DNA, with or without 0.5 mM NAD^+, and indicated concentrations of ABT-888. (**I**) Top: Gel-based autoPARylation assay performed with 4 μM mCherry-PARP1 with 4 μM triplex DNA, with or without 0.5 mM NAD^+, and increasing concentrations of PARG. Bottom: Fluorescence micrographs of 4 μM mCherry-PARP1 condensates formed in the presence of 4 μM triplex DNA, 0.5 mM NAD^+, and 125 nM PARG. $N = 1$. (**J**) Quantifications of the percent of the surface area covered by 4 μM mCherry-PARP1 with 4 μM triplex DNA with or without NAD^+ and HPF1. (**K**) Fluorescence recovery after photobleaching (FRAP) quantifications of 4 μM mCherry-PARP1 with 8 μM dumbbell DNA in PARylation Buffer with and without NAD^+. All figures represent findings from at least 3 biological replicates in 20 mM Tris pH 7.5, 50 mM NaCl, 7.5 mM $MgCl_2$, and 1 mM DTT unless specified otherwise. Error bars indicate the standard error of the mean. *p* values are obtained from a student's *t*-test: n.s. non-significant.

 © The Author(s)

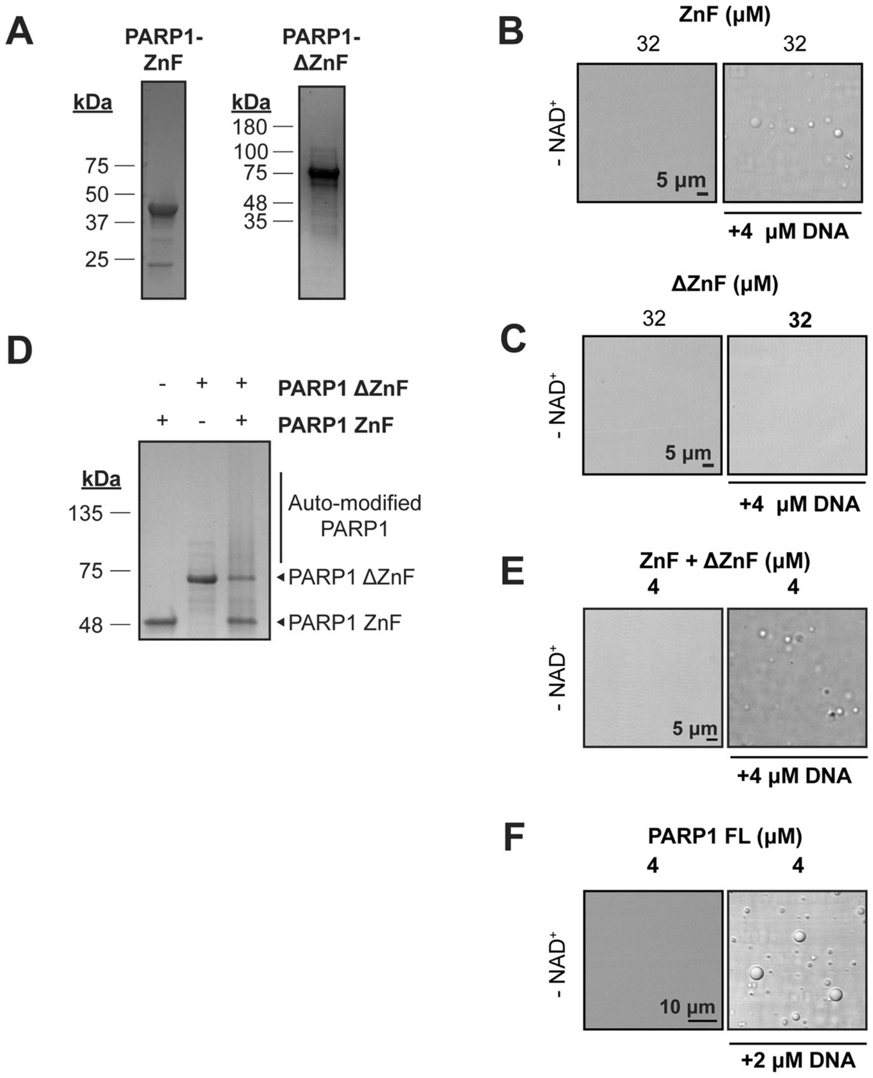

Figure EV3. The ZnF region is important for PARP1 condensation.

(**A**) Coomassie-stained SDS-PAGE gels of purified recombinant PARP1 truncation constructs. (**B**) DIC micrographs of ZnF PARP1 protein with and without triplex DNA. (**C**) DIC micrographs of ΔZnF PARP1 protein without triplex DNA. (**D**) Gel-based autoPARylation assay of 1 μM truncated PARP1 proteins in the presence or absence of 0.5 mM NAD^+, analyzed 10 min after NAD^+ addition. The absence of a protein band or smearing in assays containing NAD^+ indicates modification with PAR. (**E**) DIC micrographs of ZnF and ΔZnF PARP1 protein fragments mixed together with 4 μM triplex DNA. (**F**) DIC micrographs of full-length PARP1 protein with and without triplex DNA. All figures represent findings from at least three biological replicates in 20 mM Tris pH 7.5, 50 mM NaCl, 7.5 mM $MgCl_2$, and 1 mM DTT unless specified otherwise.

© The Author(s)

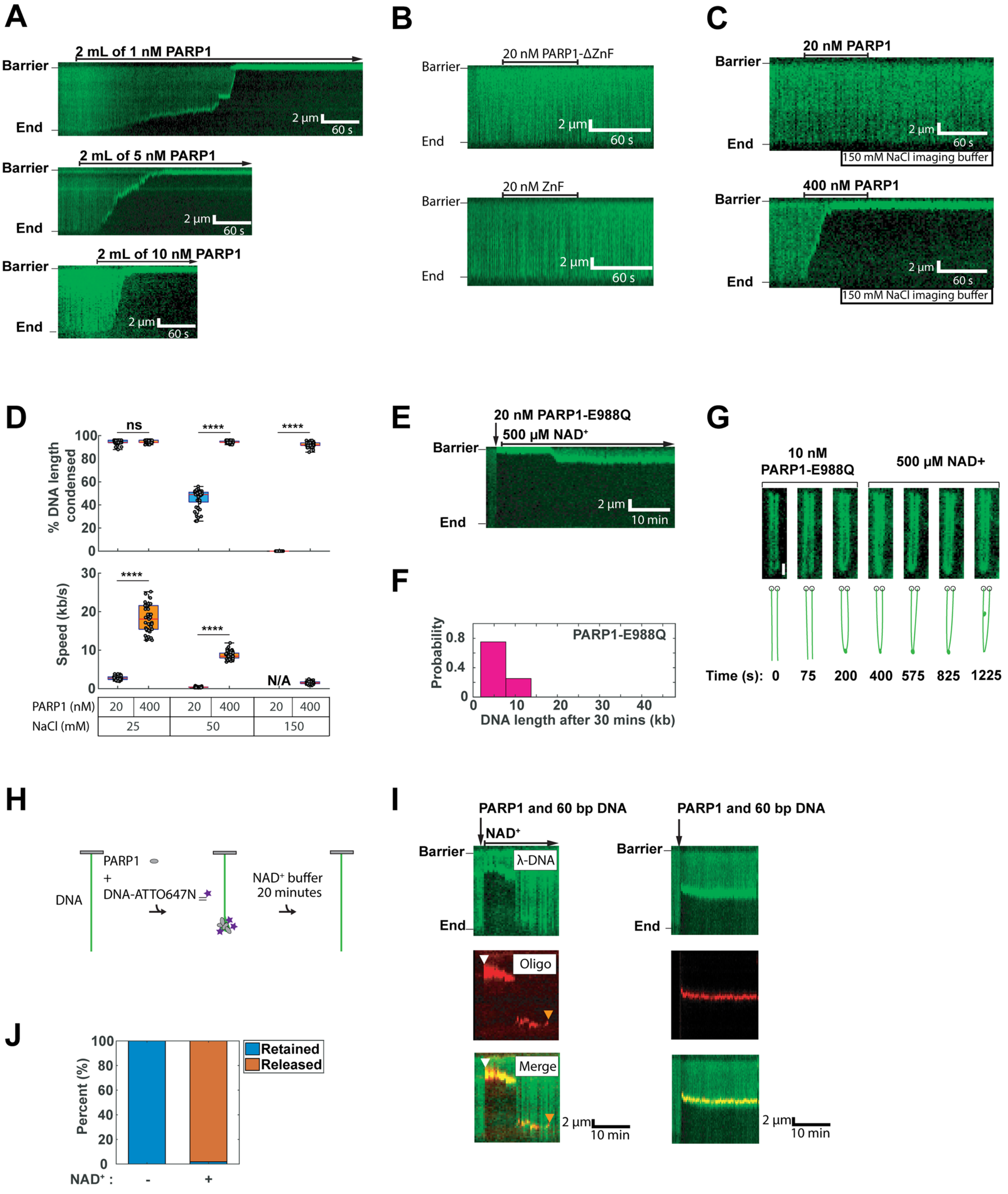
A
2 mL of 1 nM PARP1
Barrier
End
2 μm
60 s
2 mL of 5 nM PARP1
2 mL of 10 nM PARP1
B
20 nM PARP1-ΔZnF
20 nM ZnF
C
20 nM PARP1
400 nM PARP1
150 mM NaCl imaging buffer
D
% DNA length condensed
Speed (kb/s)
ns
****
N/A
PARP1 (nM)
NaCl (mM)
E
20 nM PARP1-E988Q
500 μM NAD+
10 min
F
PARP1-E988Q
Probability
DNA length after 30 mins (kb)
G
10 nM PARP1-E988Q
500 μM NAD+
Time (s): 0 75 200 400 575 825 1225
H
DNA
PARP1
+
DNA-ATTO647N
NAD+ buffer
20 minutes
I
PARP1 and 60 bp DNA
NAD+
λ-DNA
Oligo
Merge
J
Percent (%)
Retained
Released
NAD+ :

Figure EV4. PARP1 condensates bridge broken DNA ends.

(**A**) Kymographs showing the compaction of DNA (green) induced by the injection of 1, 5, and 10 nM PARP1, respectively. (**B**) DNA curtains assay with 20 nM of ΔZnF and ZnF mutants of PARP1. Neither mutant can compact DNA at this concentration. (**C**) Kymographs illustrating DNA compaction by 100 µL of 20 nM PARP1 (top) and 400 nM PARP1 (bottom) with 150 mM NaCl. (**D**) Quantification of DNA compaction by 100 µL of 20 nM PARP1 or 400 nM PARP1 with 25 mM NaCl, 50 mM NaCl, or 150 mM NaCl. Boxplots denote the first and third quartiles of the data. The center lines in the box indicate the median. The upper whisker is the maximum value and the lower whisker is the minimum value. At least 45 DNA molecules were analyzed for each condition. *p* values are obtained from a two-tailed *t*-test: ****$p < 0.0001$, ns: not significant. The *p* value for the condensed DNA length between 20 nM PARP1 and 400 nM PARP1 in 50 mM NaCl buffer condition is 9.1795e-18 and in 150 mM NaCl buffer condition is 4.4296e-19. The *p* value for the speed between 20 nM PARP1 and 400 nM PARP1 in 25 mM NaCl buffer condition is 5.1175e-17 and in 50 mM NaCl buffer condition is 1.1155e-17. (**E**) Kymograph showing DNA compaction after injection of 100 µL of 20 nM PARP1-E988Q, followed by DNA extension with buffer containing 500 µM NAD^+ for 30 min. (**F**) Histogram of DNA resolving experiments from E). $N = 51$. (**G**) Frames from a movie showing the end-to-end bridging of two DNA molecules following the injection of 100 µL of 10 nM PARP1-E988Q. This bridge is not reversed by injecting 500 µM NAD^+. Scale bar: 2 µm. (**H**) Schematic of the 60 bp dsDNA oligo capture and dissociation experiment. (**I**) Kymographs showing the capture of 60 bp DNA-ATTO647N induced by PARP1. A mixture of 100 µL, containing 5 nM PARP1 and 20 nM DNA-ATTO647N, was injected into the flowcell and incubated with tethered λ-DNA (green) with flow turned off. After a 5-min incubation, imaging acquisition resumed, revealing the capture of DNA-ATTO647N (red) on the long DNA. Subsequently, the imaging buffer was supplemented with (left) or without (right) 500 µM NAD^+ buffer. On the left panel, the white arrow marks the time when the dsDNA oligo were captured, while the orange arrow indicates when the dsDNA oligo dissociated following NAD^+. (**J**) Bar graphs depicting the percentage of 60 bp DNA retained and released from λ-DNA following a 20-min chase period with imaging buffer with or without the addition of 500 µM NAD^+. Nearly all oligos are released when NAD^+ is in the flowcell. At least 57 DNA molecules were analyzed for each condition.

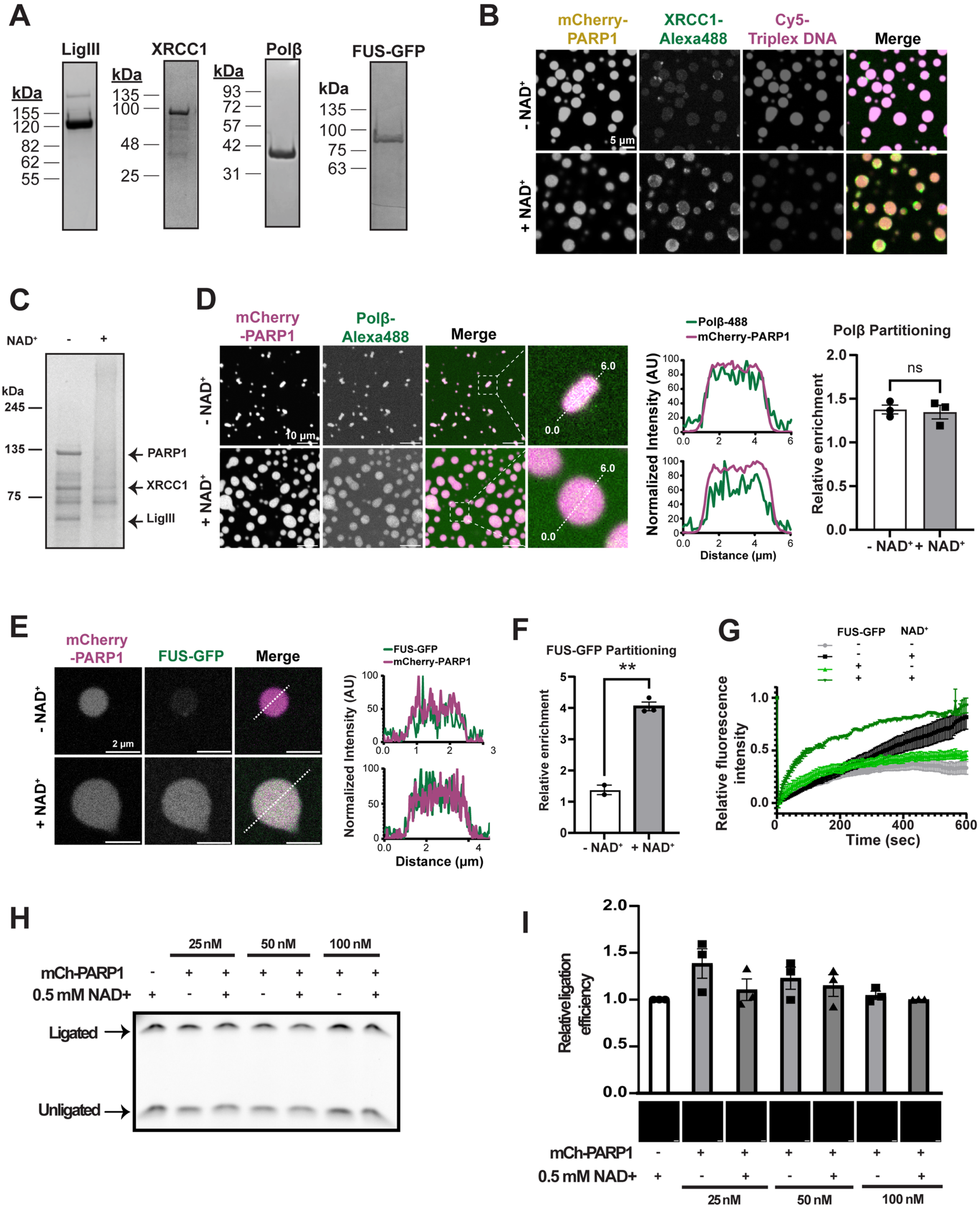
A
LigIII
XRCC1
Polβ
FUS-GFP
kDa
155
120
82
62
55
135
100
48
25
93
72
57
42
31
135
100
75
63
B
mCherry-PARP1
XRCC1-Alexa488
Cy5-Triplex DNA
Merge
- NAD+
+ NAD+
5 µm
C
NAD+
245
135
75
PARP1
XRCC1
LigIII
D
mCherry-PARP1
Polβ-Alexa488
Merge
- NAD+
+ NAD+
10 µm
6.0
0.0
Polβ-488
mCherry-PARP1
Normalized Intensity (AU)
Distance (µm)
Polβ Partitioning
ns
Relative enrichment
- NAD+ + NAD+
E
mCherry-PARP1
FUS-GFP
Merge
- NAD+
+ NAD+
2 µm
FUS-GFP
mCherry-PARP1
Normalized Intensity (AU)
Distance (µm)
F
FUS-GFP Partitioning
**
Relative enrichment
- NAD+ + NAD+
G
FUS-GFP
NAD+
Relative fluorescence intensity
Time (sec)
H
25 nM
50 nM
100 nM
mCh-PARP1
0.5 mM NAD+
Ligated
Unligated
I
Relative ligation efficiency
mCh-PARP1
0.5 mM NAD+
25 nM
50 nM
100 nM

 © The Author(s)

Figure EV5. SSBR proteins partition together in condensates that enrich DNA.

(**A**) Images of Coomassie blue stained SDS-PAGE gels of purified recombinant LigIII, XRCC1, Polβ, and FUS-GFP. (**B**) Fluorescence micrographs of 4 μM mCherry-PARP1, 4 μM triplex DNA (10% Cy5-labeled), and 1 μM XRCC1 (10% AlexaFluor488-labeled) with and without NAD^+. Quantifications of the XRCC1 partitioning are shown in Fig. 5B. (**C**) Coomassie blue stained SDS-PAGE gel of PARP1, XRCC1, LigIII, triplex DNA, with and without NAD^+. Note the disappearance of a strong band at the molecular weight of LigIII, XRCC1, and PARP1 with NAD^+, indicating their PARylation. $N = 1$. (**D**) Right: Fluorescence micrographs of 4 μM mCherry-PARP1, 8 μM dumbbell DNA, and 1 μM Polβ (10% AlexaFluor488-labeled) with and without NAD^+. Middle: The fluorescence intensity measured across the diameter of a representative condensate (dashed white line) is plotted on the right. Left: Quantification of Polβ enrichment within condensates, as calculated by the mean fluorescence intensity in the condensed phase relative to that of the dilute phase. (**E**) Fluorescence micrographs of 4 μM mCherry-PARP1, 1 μM triplex DNA, and 1 μM FUS-GFP with and without NAD^+. The fluorescence intensity measured across the white dashed lines is plotted. (**F**) Quantifications of the FUS-GFP enrichment within PARP1 condensates shown in (**E**). p value = 0.0024. (**G**) Fluorescence recovery after photobleaching (FRAP) quantifications of 4 μM mCherry-PARP1 with 1 μM triplex DNA with and without 1 μM FUS-GFP in PARylation Buffer with and without NAD^+. (**H**) Gel-based ligation assay was performed with 10 nM LigIII, 10 nM XRCC1, 50 nM Cy5-triplex DNA, and the indicated concentration of mCherry-PARP1 and 0.5 mM NAD^+, analyzed 15 min after ATP addition. The presence of a DNA band at a higher molecular weight indicates ligation. (**I**) Top: Quantifications of ligation efficiency calculated as the fluorescence intensity of Cy5-triplex DNA in the ligated relative to unligated in a gel-based assay shown in (**H**); Bottom: Representative fluorescence micrographs of mCherry-PARP1 in the ligation reactions after ATP addition. All figures represent findings from at least three biological replicates in 20 mM Tris pH 7.5, 50 mM NaCl, 7.5 mM $MgCl_2$, and 1 mM DTT unless specified otherwise. Error bars indicate the standard error of the mean. p values are obtained from a student's t-test: $^{**}p < 0.01$, n.s. non-significant.

© The Author(s)

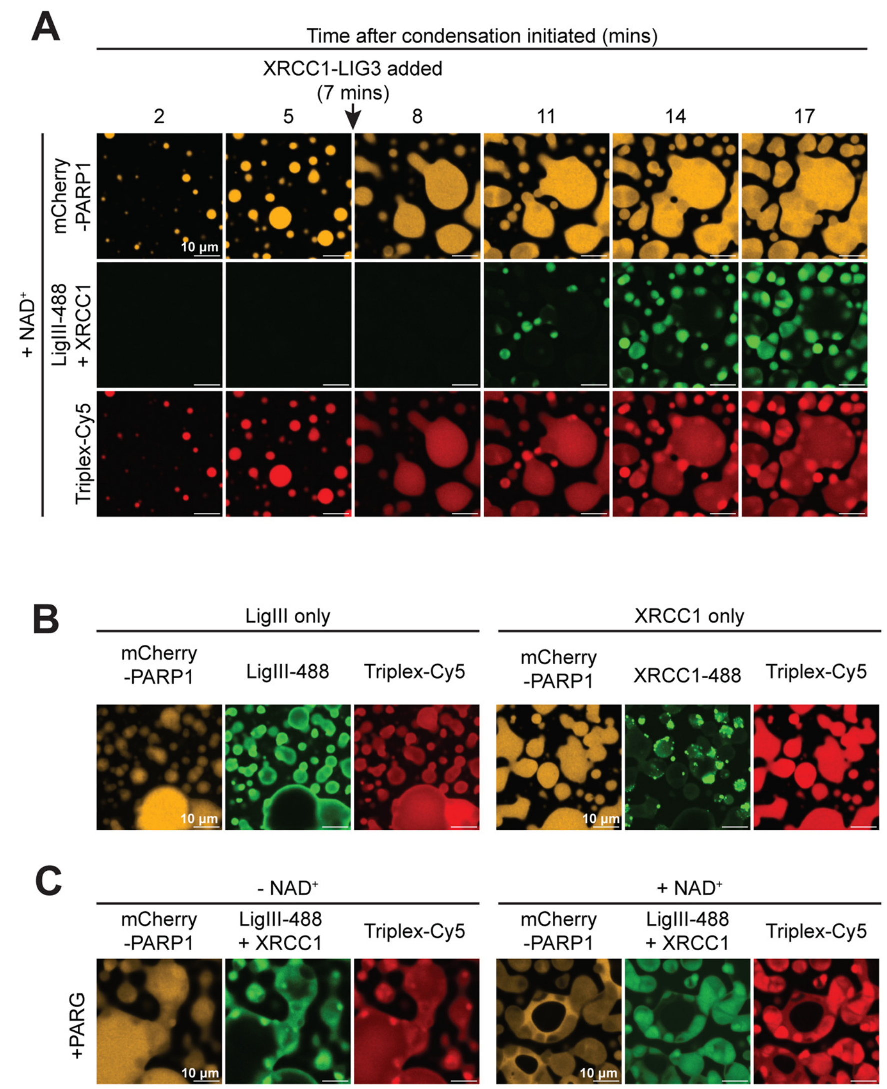

Figure EV6. SSBR proteins partition together in condensates that enrich DNA.

(**A**) Fluorescence micrograph of 4 µM mCherry-PARP1 with 4 µM Triplex DNA and 1 µM XRCC1 and LigIII complex with 0.5 mM NAD+. Imaging started 2 min after PARP1 and DNA were mixed, XRCC1 and LigIII mixture was added 5 min after. (**B**) Fluorescence micrograph as in A) with LigIII and XRCC1 separately at ~20 min after imaging. (**C**) Fluorescence micrograph as in (**A**) with 0.5 µM PARG. PARG was added 5 min after the addition of XRCC1 and LigIII.

© The Author(s)
